# Stepwise emergence of recombination suppression precedes fissiparous asexuality in the planarian *Schmidtea mediterranea*

**DOI:** 10.1038/s41467-026-74605-9

**Published:** 2026-06-26

**Authors:** Jeremias N. Brand, Ajinkya Bharatraj Patil, Luca Pandolfini, Kira Zadesenets, Nikolay Rubtsov, Laura Robledillo, Meng Zhang, André Marques, Jochen C. Rink

**Affiliations:** 1 Department of Tissue Dynamics and Regeneration, Max Planck Institute for Multidisciplinary Sciences, Göttingen, Germany; 2 Zoological Institute, Department of Environmental Sciences, University of Basel, Basel, Switzerland; 3 Center for Human Technologies, Non-coding RNA and RNA-based therapeutics, Istituto Italiano di Tecnologia, Genova, Italy; 4 The Federal Research Center Institute of Cytology and Genetics SB RAS, Novosibirsk, Russia; 5 Novosibirsk State University, Novosibirsk, Russia; 6 Department of Chromosome Biology, Max Planck Institute for Plant Breeding Research, Cologne, Germany; 7 Faculty of Biology und Psychology, Georg-August-University Göttingen, Göttingen, Germany

**Keywords:** Molecular evolution, Population genetics, Haplotypes, Sexual selection

## Abstract

A central paradox in evolutionary biology is the rarity of asexual reproduction, often attributed to developmental constraints and long-term costs. Yet, fissiparous asexuality—where animals split and regenerate—is widespread among planarians, hinting at genomic features predisposing them to asexuality. We investigate the genomic underpinnings and evolutionary consequences of asexuality in the planarian *Schmidtea mediterranea*, which exists as both obligately fissiparous and sexual strains. We generate a haplotype-phased genome assembly of the asexual strain and collect population genomic data to uncover extensive heterozygous chromosomal rearrangements affecting all chromosomes. We show that these rearrangements arose in a sexually reproducing ancestor without directly disrupting reproductive genes but instead progressively suppressing recombination across the genome. The asexual genome exhibits minimal deleterious mutation accumulation, indicating a low cost of asexuality. Population-genomic data indicate that persistent asexuality originated recently (0.17–0.4 Ma), however the young age is insufficient to explain the low mutational burden. Instead, planarians may exploit the lack of a single-cell bottleneck in fissiparous reproduction to mitigate the costs of asexuality. Altogether, our results are consistent with a model in which stepwise recombination suppression due to structural rearrangements eroded the benefits of sex and enabled the emergence of fissiparous asexuality in *S. mediterranea*.

## Abstract (web summary)

Asexuality has evolved many times in planarians, but its genomic basis is unknown. This study shows that in the species *S. mediterranea*, a series of chromosomal rearrangements in a sexual ancestor suppressed recombination genome-wide, followed by the emergence of asexual reproduction with surprisingly little mutational cost so far.

## Introduction

Sexual reproduction is predicted to be more costly than asexual reproduction^1,2^ due to three main factors: the two-fold cost of males^2^, the cost of recombination breaking favorable combinations of alleles^3^, and the costs associated with mating^4^. Consequently, asexual mutants should spread throughout a population and drive sexual lineages to extinction^1^. The explanation for why sexual reproduction remains the rule rather than the exception despite these costs is thought to be the evolutionary advantages sex confers through efficient selection^1,5^, especially in heterogeneous environments^6,7^ or during coevolution with parasites^8,9^. Sexual recombination can reduce negative epistasis^10^, avoid Hill-Robertson interference^10^ by allowing beneficial mutations to spread independently of harmful ones^11,12^, and prevent the accumulation of deleterious mutations in small populations (Muller’s ratchet)^11^, by restoring the least-loaded genotype. Consequently, asexual lineages deprived of the advantages of sexual reproduction should accumulate deleterious mutations over time and ultimately go extinct—a process known as mutational meltdown^5,11^. However, while numerous studies find increased accumulation of deleterious mutations in parthenogenetic asexuals^13–19^, some ancient asexual lineages are associated with equal^20^ or even more efficient purifying selection compared to their sexual relatives^21,22^. This suggests that some asexual lineages have evolved mechanisms to mitigate mutational meltdown or generate new genetic diversity^23^. In addition, alternative asexual reproduction strategies, e.g., via budding or fission of the parental organism into two new entities, a form of asexuality occurring in the majority of animal phyla^24^, have been poorly studied so far. Budding and fission are particularly interesting because they lack the single-cell bottleneck between generations that is thought to be essential for ensuring the clonality of the soma^24,25^. The increased intra-individual genetic diversity in such systems^26^ raises the potential for evolutionary conflicts, but also provides a conceptual possibility for reducing the effective mutation rate^27^, which could prevent mutational meltdown^24^.

**Fig. 1:**
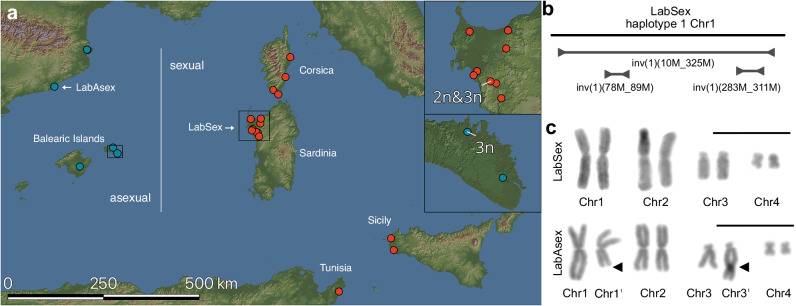
Geographic distribution and chromosomal characteristics of *Schmidtea mediterranea* sexual and asexual populations. **a** Map showing the localities where *S. mediterranea* has been found. Asexual populations (blue) have been found on the Spanish mainland and the Balearic Islands. Sexual populations (red) have been found on Corsica, Sardinia, Sicily, and Tunisia. Insets show details of collection sites on Sardinia (top) and Menorca (bottom). *S. mediterranea* is usually diploid (2*n* = 8) but, as indicated on the insets, one asexual and one sexual triploid (3*n* = 12) population have been reported^37,38,40,148^. **b** Schematic representation of Chromosome 1 (Chr1) of the sexual laboratory strain (LabSex), indicating three chromosome inversions, one of which spans almost the entire length of Chr1^48^. Population heterozygosity data show that the large inversion is present in sexual populations from Corsica and Sardinia, but absent in Sicily and Tunisia^52^. **c** Chromosome spread of LabSex and the asexual laboratory strain (LabAsex). Arrows indicate the reciprocal translocation between Chr1 and Chr3, characteristic of LabAsex, resulting in a short, derived Chr1′ and a long, derived Chr3′. Representative images from 50 metaphase plates across 10 individuals in both the sexual and asexual strains. Scale bars represent 10 μm. For details on the abbreviations used in this study, see Table 1.

Planarian flatworms are an excellent model to understand the emergence and consequences of fissiparous asexuality, because the hundreds of planarian species worldwide display a fascinating spectrum of reproductive strategies, ranging from hermaphroditic sexual reproduction to parthenogenesis, and asexual fissiparous reproduction^28^. The molecularly tractable laboratory model species *Schmidtea mediterranea* is of particular interest. Usually studied for its whole-body regeneration ability and abundant adult pluripotent stem cells^29,30^, this species exists in both asexual and sexual strains. The asexual strains reproduce exclusively by fission and subsequent whole-body regeneration and never develop sexual organs. The sexual strains develop hermaphroditic reproductive organs and lay egg capsules containing multiple fertilized zygotes. The planarian research community primarily studies a clonal strain (CIW4) originating from a single animal collected from a fountain in Montjuïc Park, Barcelona, Spain, in 1998^31–33^. The CIW4 strain is exclusively fissiparous and never develops reproductive organs. The somewhat less-studied exclusively sexual laboratory strains were established from specimens collected in Sardinia and do not undergo fission^34,35^. *S. mediterranea* has a restricted distribution in the western Mediterranean, with asexual populations on the Catalan coast and the Balearic Islands and sexual populations in Corsica, Sardinia, Sicily, and the Tunisian coast^36–44^. No co-occurrence between the two strains has been reported^36–44^ (Fig. 1a). The current distribution has been hypothesized to reflect the fragmentation of an ancestral population due to microplate tectonics^38,39^, which accounts for the low dispersal ability of planarians due to their high desiccation susceptibility^28,45^. Consistently, molecular dating analyses suggest a tentative split between the sexual and asexual populations ~8.36 million years ago (Ma), but such analyses remain challenging in planarians due to the lack of accurate calibration data^46^. Overall, *S. mediterranea* offers both the comparative analysis of molecularly tractable sexual and asexual laboratory strains, as well as a biogeographical distribution range that may inform on the origins of asexuality.

The genomic causes and consequences of asexuality in planarians remain understudied due to a lack of suitable genomic resources. Planarian genomes are difficult to assemble due to extreme compositional bias (70% A/T), the abundance of unusually large repeats, and inbreeding-resistant heterozygosity^43,47–51^. Chromosome-scale assemblies of sexually reproducing strains have only recently become available, collectively revealing rapid structural divergence in planarian genomes^48,52^. The genome assembly of the sexual strain of *S. mediterranea* reveals three large inversions on chromosome 1^48,52^, which repress recombination on that chromosome^43^ (Fig. 1b), and have been interpreted as a first step in the evolution of a sex chromosome^52^. Similarly, the asexual strain of *S. mediterranea* is known to carry a cytologically visible reciprocal translocation between chromosomes 1 and 3^36^ (Chr1/3 translocation, Fig. 1c), which has been proposed as a driver of asexuality, either by disrupting reproductive system development^37^ or by inducing fission behavior^38^. However, without a genome assembly for the asexual strain, both the genomic causes and the consequences of asexuality in the system cannot be elucidated.

Here, we present the chromosome-scale diploid genome assembly of the asexual *S. mediterranea* laboratory strain, alongside extensive population genomic analyses of wild and laboratory populations, to probe its evolutionary history. We demonstrate that three successive inversions and a reciprocal translocation, originating in a sexual ancestor, progressively led to the genome-wide suppression of recombination without directly disrupting reproductive genes. Surprisingly, both laboratory and wild asexual populations exhibit minimal accumulation of deleterious mutations or other expected genomic costs. While our data rule out cryptic sex, multiple lines of evidence converge on a much younger origin of asexuality than previously thought (0.17–0.4 Ma versus 8.4 Ma). However, this recent origin remains insufficient to account for the low mutational load. This raises the possibility that selection processes among adult pluripotent stem cells may purge deleterious variants. Based on our findings, we propose a stepwise model for the evolution of asexuality in *S. mediterranea*, whereby recombination suppression through structural rearrangements gradually undermines the benefits of sex and facilitates the emergence of fissiparous asexuality to mitigate its costs.

## Results

### Genome assembly and annotation of the asexual laboratory strain

Toward our goal to examine the genomic causes and consequences of asexuality in *S. mediterranea*, we used our previous assembly strategy with 44× coverage PacBio HiFi and 137× coverage Hi-C sequences to generate a high-quality reference assembly of the asexual laboratory strain (referred to as LabAsex; see Table 1 for details on all strains used in this study). As shown in Table 2, the new LabAsex assembly (schMedA2) shows a high contiguity with ~90% of the sequence occurring on the four chromosome scaffolds. A merqury evaluation of the k-mer content with three independent short-read datasets reveals a high base-pair accuracy of the entire assembly (haplotype 1: 46.1 QV, haplotype 2: 46.9 QV) and even higher accuracy when only including the chromosome scaffolds (haplotype 1: 47.9 QV, haplotype 2: 48.2 QV). Furthermore, the k-mer analysis shows that heterozygosity is well reflected and efficiently phased between the two haplotypes (Supplementary Note 1), and the assembly is of similar completeness as our previous planarian genome assemblies^48^ as assessed using Benchmarking Universal Single-Copy Orthologs (BUSCO, Table 2). However, while the assembly is chromosome-scale, it is not telomere to telomere. 88.4 Mb and 90.7 Mb could not be scaffolded in haplotype 1 and haplotype 2, respectively. This level of contiguity is similar to our previous scaffolding success in *S. polychroa* but slightly lower than for the assembly of the sexual laboratory strain, schMedS3 (hereafter referred to as LabSex)^48^. Consequently, the ends of chromosome scaffolds were not capped by telomere repeats. Instead, these repeats were found on unincorporated scaffolds. To investigate the repetitive content, we annotated transposable elements and other highly repetitive regions using EDTA and RepeatExplorer. Similar to the sexual genome, the overall repeat content was high (haplotype 1: 61.7%; haplotype 2: 60.6%), and we were able to resolve large stretches of abundant tandem repeat elements on all Chromosomes. These tandem repeat clusters often spanned >1 Mb and comprised several distinct repeat sequences, of which some occurred in multiple blocks on multiple chromosomes (Table 3; Tables S1 and S2; see below). In addition, we improved upon our previous hybrid transcriptome assembly strategy by leveraging the complementary gene annotations from both alleles to annotate the gene content of the genome (see “Methods”). As is common with planarian genomes, we found that BUSCO completeness increased substantially when running in transcriptome mode (Table 2). Functional annotation based on homology searches and domain predictions enabled annotation of 70% of all coding genes (Supplementary Note 2). Overall, the new *S. mediterranea* genome assembly provides an annotated reference assembly of an asexual planarian strain and a valuable resource for the workhorse strain of the planarian research community.

**Table 1:** *S. mediterranea* strains, populations, and genome assemblies used in this study

Abbreviation	Strain	Genome	Sample type	Origin	Reproduction	Reference
LabSex	S2F18	schMedS3	laboratory	Sardinia	Sexual	^48^
LabAsex	CIW4	schMedA2	laboratory	Barcelona	Fission	This study
SarSex	-	-	wild population	Sardinia	Sexual	This study
MenAsex	-	-	wild population	Menorca	Fission	This study

The dataset includes two laboratory populations with genome assemblies and two wild populations. The asexual genome assembly (schMedA2) of strain CIW4 complements the previously published sexual assembly of strain S2F18. For simplicity, strains and populations are referred to throughout the text using the indicated abbreviations.

**Table 2:** Summary statistics for the genome assemblies and annotations of both *S. mediterranea* LabAsex haplotypes

Genome assembly statistics	Haplotype 1	Haplotype 2
Quality value	46.1	46.9
Quality value chromosomes	47.9	48.2
Base accuracy (%)	99.9975	99.9979
Base accuracy chromosomes (%)	99.9984	99.9984
Scaffolds (#)	1120	550
Contigs (#)	1258	699
Total length (bp)	853,691,854	836,358,679
Scaffolded (bp)	765,292,180	758,381,961
Scaffolded (%)	89.6	90.7
Unscaffolded (Mb)	88.4	78
GC (%)	29.66	29.49
Scaffold N50	272,298,851	224,520,763
Contig N50	8,105,788	7,438,653
BUSCO complete (%) (single copy, duplicated)	68.1 (62.6, 5.5)	68.9 (63.1, 5.8)
BUSCO fragmented (%)	9.3	9.4
BUSCO missing (%)	22.6	21.7
**Transcriptome statistics**		
Genes (#)	60,931	60,595
Transcripts (#)	104,163	103,465
Coding sequences (#)	44,796	44,533
Total gene length (bp)	355,698,801	355,185,528
Total transcript length (bp)	337,843,474	336,471,699
Median gene length (bp)	791	790
Median transcript length (bp)	1635	1633
longest gene (bp)	338,920	314,464
shortest gene (bp)	80	80
Gene coverage (%)	41.7	42.5
Coding sequence coverage (%)	3.4	3.5
BUSCO complete (%)	79.1	80
BUSCO fragmented (%)	3	3.4
BUSCO missing (%)	17.9	16.6

Quality value and base calling accuracy are the average of three independent short-read datasets of the laboratory culture.

**Table 3:** The number and size of large tandem repeat elements detected in the LabAsex and LabSex genome assemblies

Genome	Tandem repeat	*N*	*N* > 1 Mb	Min. (Mb)	Max. (Mb)	Total (Mb)
LabAsex haplotype 1	158mer	12	5	0.16	2.41	11.65
	159mer	3	1	0.31	1.45	2.11
	pericentromeric	6	2	0.45	1.9	6.29
LabAsex haplotype 2	158mer	9	3	0.26	2.54	8.56
	159mer	4	0	0.12	0.33	1.02
	pericentromeric	5	0	0.11	0.67	2.18
LabSex haplotype 1	158mer	14	4	0.12	3.34	13.5
	159mer	5	3	0.72	5.31	10.67
	pericentromeric	6	3	0.19	1.87	5.39
LabSex haplotype 2	158mer	11	6	0.23	2.26	12.84
	159mer	4	3	0.44	5.1	9.48
	pericentromeric	4	2	0.21	1.38	3.04

The largest and most abundant repeat consisted of a 158 bp k-mer (158mer), and the second largest repeat consisted of a 159 bp long k-mer (159mer). In addition, we identified four tandem repeats as putative centromeric because they were chromosome-specific and located as expected from karyology (CL66: 424 bp; CL73: 544 bp; CL75: 693 bp; CL79: 766 bp).

### Structural analysis of the asexual genome assembly

**Fig. 2:**
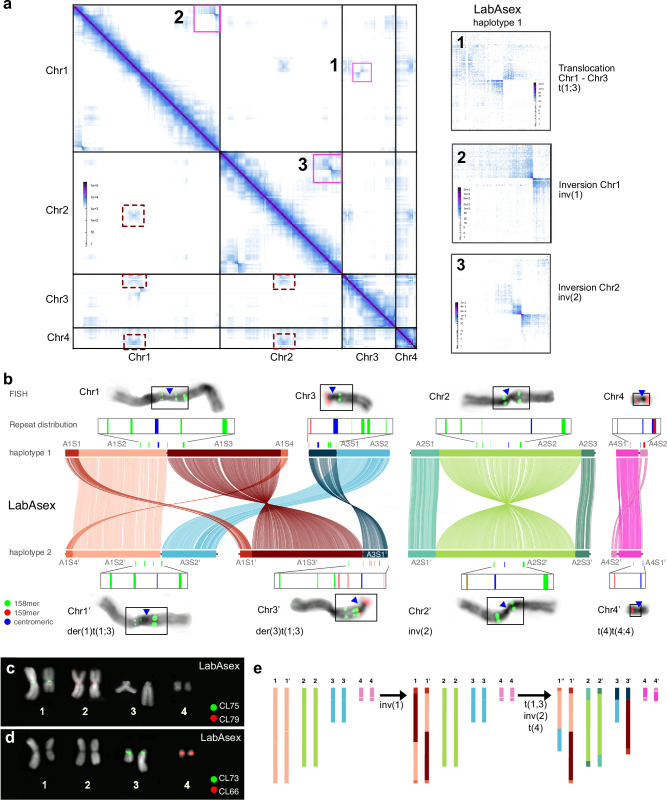
Haplotype-phased genome assembly of the asexual *Schmidtea mediterranea* laboratory strain (LabAsex). **a** Hi-C chromatin interaction heatmap of LabAsex haplotype 1 (137× Hi-C coverage). The symmetric pattern indicates high contiguity within chromosome scaffolds. Boxes in the lower triangle highlight increased inter-chromosomal contacts at centromeres. Numbered boxes in the upper triangle highlight long-distance contacts characteristic of structural variation between haplotypes, shown at higher resolution in the corresponding panels: (1) Chr1/Chr3 contact from the Chr1/3 translocation; (2) end-to-end Chr1 contact from a large inversion; (3) end-to-end Chr2 contact from a large inversion. Structural variants are described in ISCN notation (*inv* inversion, *t* translocation, *der* derived chromosome). **b** Synteny between LabAsex haplotype 1 and haplotype 2. The central diagram shows whole-genome alignment with genomic strata (colored bars and connecting lines), tandem repeat distribution (158mer, 159mer), and putative pericentromeric repeats. Flanking chromosome images show fluorescent in situ hybridization (FISH) with probes targeting the 158mer and 159mer repeats; blue triangles mark centromere positions. See Supplementary Note 5 for original images. Black lines below syntenic blocks represent the genome sequence; the bottom row gives ISCN descriptions of derived chromosomes in haplotype 2. Chr2′ of haplotype 2 is shown inverted to aid visualization of the large inversion. **c**, **d** FISH using directly labeled probes targeting putative pericentromeric repeat regions. **e** Most parsimonious sequence of chromosomal rearrangements. The Chr1/3 reciprocal translocation most likely occurred on an already-inverted Chr1. Because the Chr1 inversion is also present in the LabSex strain—which lacks all other rearrangements—it is inferred to have occurred first, with subsequent rearrangements accumulating in the asexual lineage (Supplementary Note 3). Source data are provided as a Source data file.

Planarian genomes are generally known to undergo rapid structural evolution^48^, and the asexual strain of *S. mediterranea* has previously been shown to carry a reciprocal Chr1/3 translocation^36^. With the LabAsex assembly in hand, we first analyzed its structure in comparison to the sexual laboratory strain (LabSex) reference genome assembly^48^ via Hi-C chromosome conformation capture (Supplementary Note 3). The mapping of the LabAsex Hi-C data on LabAsex haplotype 1 revealed multiple clear off-diagonal signals as typical signatures of structural rearrangements (Fig. 2a). The off-axis signal between Chr1 and Chr3 (Fig. 2a, solid box 1) was consistent with the known translocation, indicating its successful recovery in the LabAsex assembly. Two additional off-axis signals (Fig. 2a, solid box 1) suggested large inversions on Chr1 and Chr2 (Fig. 2a, solid boxes 2, 3). Interestingly, we further noticed a pronounced increase in contact signal between the centromeres (Fig. 2a, dashed boxes), suggesting centromere clustering typical of a Type I architecture, which has also been observed in the parasitic flatworm *Clonorchis sinensis*^53^. To complement the Hi-C maps, we directly compared the two LabAsex haplotypes by whole-genome alignment. As expected, the whole-genome alignments reflected the structural differences suggested by Hi-C (Fig. 2b). Specifically, the size and position of the chromosome segments affected by the Chr1/3 translocation matched well with predictions from the literature (Supplementary Note 4), and large inversions on Chr1, Chr2, and possibly Chr4 were also apparent. However, the haplotype differences on Chr4 were not analyzed further due to its particularly high repeat content and correspondingly lower confidence in its long-range contiguity (Supplementary Note 3). Surprisingly, our whole-genome alignments revealed that the large inversion on Chr1 of the LabAsex assembly was identical with the LabSex assembly, indicating that the inversion was already present in their common ancestor (Fig. 2a, Supplementary Note 3; see below). However, the two additional inversions on Chr1 in the LabSex assembly (Fig. 1b) were not detected in the LabAsex assembly, suggesting continued genome rearrangements in both strains after their divergence (Supplementary Note 3). Overall, our new asexual strain reference genome assembly accurately reflects the known Chr1/3 heterozygous translocation and additional genome rearrangements, thereby generalizing our previous findings of extensive structural genome divergence between *Schmidtea* species^48^ to different strains of *S. mediterranea*.

### Assembly verification by in situ hybridization

To independently confirm the various indications of chromosomal rearrangements in the LabAsex assembly, we thought to exploit the distinct repeat sequences of the large tandem repeat clusters and their predicted spacing in individual chromosomes. In situ hybridization (FISH) probes designed against the consensus sequence of two abundant tandem repeats (referred to as 158mer and 159mer) yielded bright and distinct banding patterns on metaphase chromosomes of *S. mediterranea* (Fig. 2b, see “Methods”). The quantitative analysis of observed versus predicted patterns as a structural bar code in 50 metaphase plates from 10 sexual and asexual specimens each largely confirmed the expected patterns shown in Fig. 2b, despite some variation in signal intensity due to variable chromosome compaction. Specifically, the weaker 158mer signal on Chr1 compared to Chr1′ (Supplementary Note 5) and higher signals for both the 158mer and 159mer on Chr3′ confirmed the mapping of the Chr1/3 translocation. In addition, the FISH patterns confirmed the predicted loss of 159mer signal on Chr4′, which together confirms the structural accuracy of the LabAsex assembly.

During the course of these analyses, we noticed large tandem repeat clusters at the approximate position of the centromeres of each of the four chromosomes, but interestingly, the repeat sequence was different for each chromosome (Fig. 2b; Table 3). FISH against each of the four repeats indeed resulted in bright labeling in the vicinity of the centromere, yet with remarkable specificity for individual chromosomes (Fig. 2c, d). While further studies will be necessary for the functional definition of centromere sequences in *S. mediterranea*, the chromosome-specific pericentromeric repeats enabled us to link genome sequences to corresponding chromosomes and provided additional experimental confirmation of the long-range contiguity of the LabAsex assembly.

Accordingly, LabAsex haplotype 1 contains chromosomes similar to chromosomes of both haplotypes of the LabSex assembly (Chr1-4), while LabAsex haplotype 2 contains rearranged chromosomes with the reciprocal balanced Chr1/3 translocation resulting in a shorter derived Chr1′ and a longer derived Chr3′. On the basis of sequence homology alone, the rearranged Chr3′ would align with Chr1 due to the translocated long arm of Chr1, which illustrates the utility of the unique pericentromeric regions in understanding the structural genome organization. Furthermore, haplotype 2 contains an inverted Chr2′ and a derived Chr4′ due to a transposition of a distal region and loss of a 159mer repeat region (Fig. 2b). As previously noted, Chr4′ is misscaffolded in the current assembly, and further efforts, such as ultra-long sequencing, will be needed to resolve the correct structure of Chr4′. Despite these substantial large-scale rearrangements, synteny between LabAsex and LabSex is highly conserved and can be used to reconstruct the evolutionary history of structural changes (Supplementary Note 6). Our data suggest the succession of chromosomal translocations and inversions depicted in Fig. 2e as the most parsimonious evolutionary explanation for the current asexual genome structure. Importantly, if the Chr1 inversion preceded the Chr1/3 translocation, this would explain the preservation of homology at chromosome ends through only two structural rearrangement events, whereas alternative scenarios would require at least three inter-chromosomal translocations or even more complex sequences of fissions, fusions, and inversions. This finding is conceptually important because it links the evolution of the LabAsex strain to the gradual accumulation of genomic rearrangements (see “Discussion”).

### Translocation breakpoint analysis

**Fig. 3:**
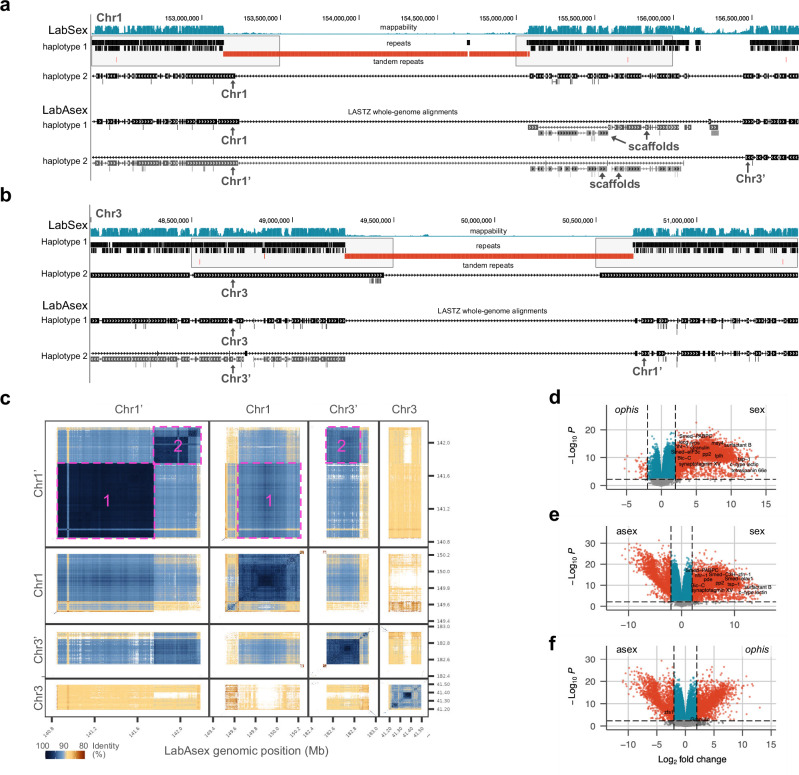
Genomic context of the Chr1/3 translocation in the sexual (LabSex) and asexual (LabAsex) laboratory strains. **a**, **b** UCSC genome browser views with haplotype 1 of the LabSex assembly as reference. Tracks show genome mappability, repeat content, and whole genome alignment tracks of the region identified as homologous to the breakpoint in the LabAsex assembly on **a** Chr1 and **b** Chr3. Note that the apparent extension of the Chr1′ alignment across the breakpoint in (**a**) reflects netting of repetitive elements (2x BURRO, 1x RND-4-family) rather than true synteny. Gray boxes outline the regions evaluated for gene loss and/or changes in gene regulation in Supplementary Note 8. **c** Similarity heatmap of 158mer repeats in the LabAsex assembly showing the high similarity within and between the breakpoint regions. Each box shows similarity in 2 kb windows of regions to themselves and between them. Numbered boxes highlight increased similarity of the Chr1′ repeats with (1) Chr1 and (2) Chr3′, indicating that the translocation breakpoint lies between them. **d**–**f** Volcano plots showing differential expression analysis (limma moderated t-test, two-sided, Benjamini-Hochberg correction for multiple comparisons; significance thresholds: |log2FC| ≥ 2, adjusted *p* < 0.01) used to identify reproduction-related genes by comparing **d** sexual control (wild-type and eGFP RNAi) vs. *ophis* RNAi, **e** sexual control vs. asexuals wild-type, and **f** *ophis* RNAi vs asexual wild-type. The 2136 high-confidence reproduction-related genes are defined as those that are significantly upregulated in sexual control in both (**d**) and (**e**). Source data are provided as a Source data file.

Given previous speculations that the reciprocal translocation between Chr1 and Chr3 might be causally linked to the loss of reproductive system development in the asexual strain^37,38^, we next examined the breakpoints in more detail. Using Hi-C mapping and genome alignment, we identified the breakpoints in the LabAsex assembly and located the homologous regions in the LabSex assembly. Interestingly, in both assemblies, the breakpoints clearly map to tandem repeat clusters. In the LabSex assembly, the breakpoint region spans a 1.9 Mb region consisting of 158mer repeats on Chr1 and a 1.4 Mb region of 158mer repeats on Chr3 (Fig. 3a, b; Supplementary Note 6). After identifying the breakpoint, we selected haplotype 1 of LabSex as our reference, allowing for direct comparison of its synteny with haplotype 2 and the LabAsex assembly via whole-genome alignment. As expected, LabSex haplotype 2 aligned in inverted orientation across the repeat stretch on Chr1 (Fig. 3a, first row). Similarly, LabAsex haplotype 1 aligned across the breakpoint, although downstream sequences were on unincorporated scaffolds, likely due to a flanking ~250 kb low-mappability region. The alignment of LabAsex haplotype 2 revealed the Chr1/3 translocation with a transition from Chr1′ to unincorporated scaffolds and then to Chr3′. On Chr3, alignments around the breakpoint were simpler, with clean connections across the breakpoint for LabSex haplotype 2 and LabAsex haplotype 1, and a switch from Chr3′ to Chr1′ in LabAsex haplotype 2 due to the Chr1/3 translocation (Fig. 3b). Having narrowed the Chr1/3 translocation breakpoint to the 158mer repeat region, we examined the repeat variation between haplotypes by similarity heatmaps with 2 kb resolution to further home in on the precise location of the breakpoint. As shown in Fig. 3c, we detected strong self-similarity between the repeat regions on Chr1 and Chr3. On the rearranged Chr1′, two blocks were apparent: the upstream block was more similar to Chr1 and the downstream block more similar to Chr3, indicating that the Chr1/3 translocation occurred at the border between these regions (Fig. 3c) and hence deeply within the Mb-scale gene deserts of the 158mer tandem repeat clusters on Chr1 and Chr3.

To validate the lack of gene loss, we examined genes near the breakpoints using the annotation tool TOGA^54^ (Supplementary Data 3). We also identified high-confidence reproduction-related genes near the breakpoints by generating gene expression profiles from sexual and asexual wild-type animals, as well as from RNAi-treated sexual animals (eGFP control and *ophis* RNAi), the latter of which blocks early reproductive system development^55,56^ (Fig. 3d–f, Supplementary Note 7; Supplementary Data 4–8). We found no clear coding gene losses within 1 Mb of the Chr1/3 breakpoints, nor were reproduction-related genes located within 250 kb of them (Supplementary Data 9; Supplementary Note 8). Likewise, no coding gene losses or nearby reproduction-related genes were detected around the large inversions on Chr1, and only a single exon loss was observed in one gene in LabAsex haplotype 2 due to the Chr2 inversion (Supplementary Data 9). These results suggest that the large-scale structural rearrangements themselves did not disrupt coding genes. In addition, a comparison of Hi-C signals within 10 Mb flanking the breakpoint did not reveal any overt changes in chromatin conformation, consistent with a lack of regulatory effects (Supplementary Note 3). Thus, the genomic basis of asexuality in *S. mediterranea* likely lies among the many other genetic and epigenetic differences between the strains, offering a promising direction for future research.

### Genomic costs of asexuality

**Fig. 4:**
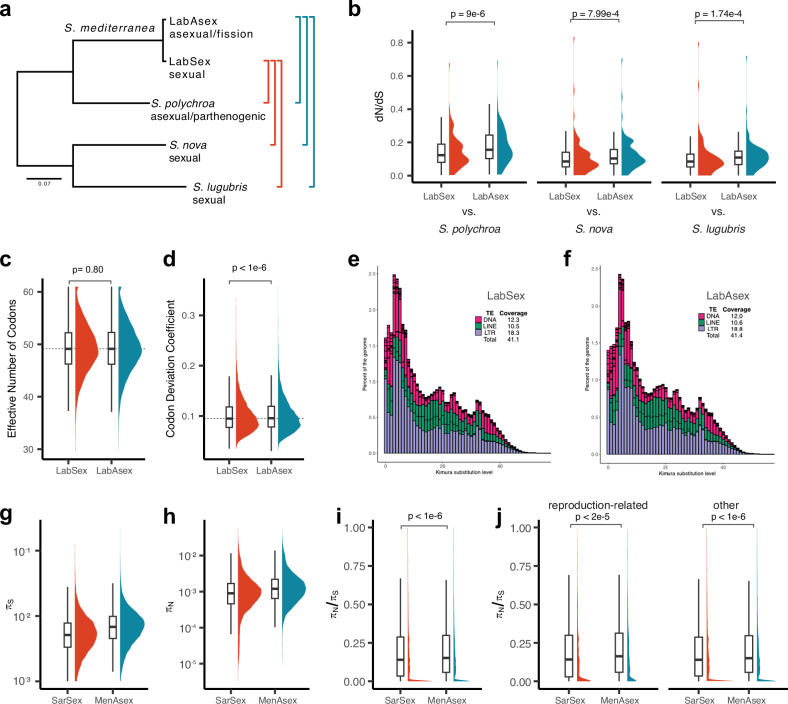
Detection of costs of asexuality in *S. mediterranea using* the sexual (LabSex) and asexual (LabAsex) laboratory strains, an asexual population from Menorca (MenAsex), and a sexual population from Sardinia (SarSex). **a** Maximum-likelihood phylogeny of the genus *Schmidtea* for *d*_*N*_/*d*_*S*_ analysis, based on codon alignments of 10,556 single-copy genes (16,684,518 positions; no missing data) inferred in IQ-TREE with gene-wise partitions and ModelFinder best-fit models. All nodes show maximal support (500 bootstraps). Colored brackets indicate pairwise comparisons in (**b**). **b** Pairwise *d*_*N*_/*d*_*S*_ comparisons for 89 genes with sufficient divergence (*d*_*S*_ > 0.1), between LabSex and LabAsex. *d*_*N*_/*d*_*S*_ was slightly elevated in LabAsex across all comparisons (*N* = 89). **c** Effective number of codons (ENC), a measure of codon usage bias (range: 20 = maximum bias to 61 = no bias), did not differ significantly between strains (*N* = 10483). **d** Codon deviation coefficient (CDC), quantifying bias relative to background composition, was significantly higher in LabSex, suggesting slightly stronger selection in sexuals (*N* = 10482). Kimura distance landscapes of repeats in the LabSex assembly (**e**) and the LabAsex assembly (**f**), showing repeat copy number (*y*-axis) vs. divergence from consensus (*x*-axis). Lower distances indicate recent TE activity; higher values reflect older elements. **g**–**j** Tests for costs of asexuality using whole-genome sequencing at 30× coverage of 20 individuals from MenAsex and 29 individuals from SarSex. **g** Nucleotide diversity (*π*) at synonymous codon positions. **h** *π* at non-synonymous codon positions. **i** Ratio of *π* at non-synonymous and synonymous codon positions (*π*_*N*_/*π*_*S*_) of 12,470 genes compared between SarSex and MenAsex. *π*_*N*_/*π*_*S*_ was significantly larger in MenAsex, indicating reduced purifying selection in asexuals. **j** *π*_*N*_/*π*_*S*_ split into 1507 high-confidence reproduction-related genes and 10,963 remaining genes. Boxplots show median and interquartile range (IQR). Whiskers extend to 1.5 × IQR. For *π*_*N*_/*π*_*S*_ analyses, only conserved genes (*π*_*N*_/*π*_*S*_ < 1) were included in the analysis. Statistical results are in Supplementary Data 12. Statistical tests in (**b**–**d**, **i**, **j**) are paired two-sided permutation tests. Source data are provided as a Source data file.

Next, we explored the possible genomic consequences of asexuality in the LabAsex assembly. Reduced purifying selection is a genomic signature frequently associated with the loss of recombination^13–19^, which can be assayed by measuring the ratio of non-synonymous (*d*_*N*_; amino-acid changing) to synonymous (*d*_*S*_; non-amino acid changing) substitutions in genes. We inferred pairwise *d*_*N*_/*d*_*S*_ for both the asexual and sexual strains of *Schmidtea mediterranea* in comparison with each of the three other *Schmidtea* species, based on 10,479 single-copy genes (see “Methods”; Fig. 4a; Supplementary Data 10). Specifically, for each sister species, we compared *d*_*N*_/*d*_*S*_ of the asexual strain to that of the sexual strain to assess whether the two strains show differences in the strength of purifying selection.

The pairwise *d*_*N*_/*d*_*S*_ values of both sexual and asexual *S. mediterranea* compared to each of the three other *Schmidtea* species were almost all «1, consistent with the general conservation of protein sequence by selection (Supplementary Data 11). Interestingly, *d*_*N*_/*d*_*S*_ in the asexual strain was consistently higher than in the sexual strain, indicating less efficient selection and thus a potential cost of asexuality. However, the effect size was extremely small (Cohen’s *d* = 0.01, Supplementary Data 12), suggesting that while statistically significant, the difference is biologically negligible. Furthermore, we noted that synonymous divergence (*d*_*S*_) between the sexual and asexual strains was generally low, which results in low power to detect differences in *d*_*N*_/*d*_*S*_ (Supplementary Data 11). When applying a common filter to retain genes with *d*_*S*_ > 0.1 between the sexual and asexual strains, only 89 genes were retained. These genes also had high *d*_*S*_ across the genus, all being among the top 15% of *d*_*S*_. Although we found no significant GO-term enrichment of these genes, they were significantly enriched for genes on our previously defined reproduction-related gene list (Fig. 3d–f; Supplementary Note 7; odds ratio = 2.22, 95% CI: 1.3–3.7, *p* = 0.003, two-sided Fisher’s exact test), which is expected since reproduction-related genes are known to evolve at an accelerated rate^57,58^. When repeating the *d*_*N*_/*d*_*S*_ analysis with this subset, we found that the increase in *d*_*N*_/*d*_*S*_ in asexuals was slightly larger (Cohen’s *d* = −0.18), again hinting at less efficient purifying selection in the asexual strain (Fig. 4b; Supplementary Data 12).

Further genomic signatures of asexuality can include changes in codon usage bias due to reduced purifying selection^59^ or the proliferation of repetitive elements due to the lack of recombination^60^. Interestingly, neither metric was significantly altered in the asexual genome assembly: Two independent measures of codon usage bias detected no or negligible differences between the two genomes (Fig. 4c, d; Supplementary Data 12 and 13). In addition, the global transposon landscapes of the two strains were highly similar, suggesting that the emergence of asexuality was not associated with overt transposon proliferation (Fig. 4e, f). In conclusion, our genome-wide comparisons revealed surprisingly few costs of asexuality in the CIW4 laboratory strain, which is all the more surprising in the face of the current estimate of the split between those two lines of ~8.4 Ma^39^.

### Population genomics of natural *S. mediterranea* populations

Since the generally low divergence between the sexual and asexual laboratory strains restricted the use of *d*_*N*_/*d*_*S*_ to only a few genes, we instead chose to elucidate the strength of purifying selection using the distribution of segregating sites in natural populations. Because the source population of the asexual laboratory strain in Barcelona has likely become extinct (pers. observations), we sampled the asexual populations on Menorca, which were described to comprise distinct diploid and triploid populations^37,38,40^. We successfully collected the diploid population (MenAsex) in a concrete canal downstream of the original type locality. The original locality had dried up due to agricultural use, suggesting that this population may be threatened in the wild. In addition, we collected wild sexual *S. mediterranea* from Sardinia (SarSex) and performed short-read whole-genome sequencing at 30× coverage of 49 individuals across both populations (20 x MenAsex, 29 x SarSex). We supplemented these data with equivalent sequencing samples from the two laboratory populations (3 x LabAsex, 3 x LabSex). Principal component analysis (PCA) and k-means clustering of genotype likelihoods of the sequencing data revealed that MenAsex was highly similar to LabAsex and SarSex clustered with LabSex, consistent with the origins of the founder animals^35^ (Supplementary Note 9). To further corroborate these results, we genotyped SarSex, MenAsex, and laboratory individuals at the COI barcoding locus and compared the sequencing reads with publicly available data^39^. A phylogenetic analysis showed MenAsex specimens clustering with LabAsex while SarSex specimens grouped with COI isolates from Corsica/Sardinia, but also showed some similarity to isolates from Sicily and Tunisia, albeit with low node support (Supplementary Note 9). This again indicates that MenAsex and LabAsex are very similar. Therefore, the MenAsex and SarSex population genomics datasets are well-suited for complementing our previous results with the laboratory strains.

To investigate the costs of asexuality in the natural populations, we first inferred nucleotide diversity (*π*) within our MenAsex and SarSex population genomics data at each nucleotide position and identified 12,470 protein-coding genes that had sufficient diversity in both populations for the following analysis (Supplementary Data 14; Note, that these do not completely overlap with the single-copy genes, See Supplementary Note 10). We then calculated the ratio of the diversity at non-synonymous (*π*_*N*_) and synonymous (*π*_*S*_) positions. The interpretation of the *π*_*N*_/*π*_*S*_ ratio is similar to *d*_*N*_/*d*_*S*_, with an expectation for the ratio to be below 1 for most proteins in the presence of efficient purifying selection. Indeed, we found that the SarSex population had significantly lower *π*_*N*_ and *π*_*S*_ compared to the MenAsex population (Fig. 4g, h), yet again with a negligible effect size across the complete gene set (Cohen *d*: −0.053, Fig. 4i; Supplementary Data 12). When partitioning the dataset based on our reproduction-related gene annotation (Fig. 3d–f), we again obtained higher *π*_*N*_/*π*_*S*_ values in MenAsex as compared to SarSex, but this time with a 50% larger effect size (Cohen *d*: −0.073, Fig. 4j; Supplementary Data 12). Our results therefore confirm the expected reduced purifying selection in the wild asexual population, especially in sexual development genes that are likely experiencing relaxed selection in asexuals. However, the effect sizes were still modest, which indicates that even wild asexual *S. mediterranea* are not in the process of undergoing mutational meltdown.

### Population history of asexuality in *S. mediterranea*

**Fig. 5:**
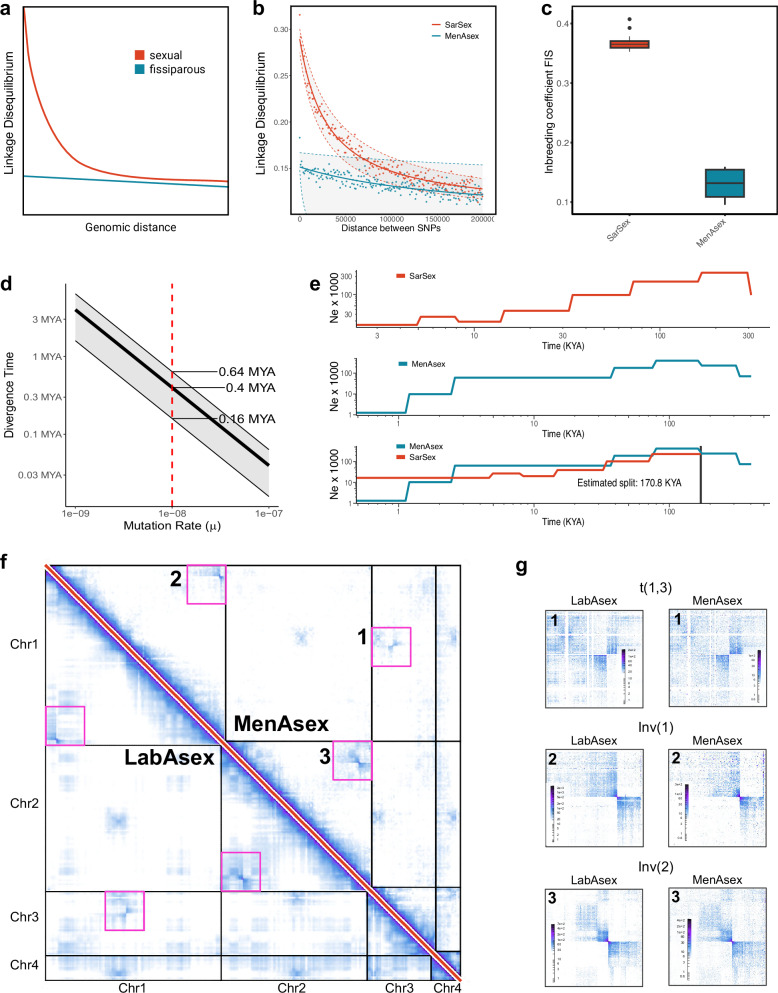
Sexual-asexual divergence estimation and evidence for persistent asexuality in wild *S. mediterranea.* **a** Expected LD decay patterns in sexual vs. asexual populations: LD drops sharply with distance in sexuals but is largely independent of genetic distance in asexuals. **b** LD decay curves inferred with ngsLD for 20 MenAsex and 29 SarSex individuals. Line and error bands represent the mean and 95% confidence intervals from 100 non-parametric bootstraps. **c** Inbreeding coefficients of SarSex and MenAsex, showing higher inbreeding in SarSex. **d** Maximum-likelihood divergence estimation based on 1,961,630 four-fold degenerate positions across 10,556 single-copy proteins. The red line indicates the empirical per-generation mutation rate in *S. mediterranea*^52^. The diagonal line and confidence envelope represent the mean and range of divergence across 1–4 generations per year. **e** Sexual-asexual divergence estimation using pairwise coalescent modeling of MenAsex and SarSex populations. **f** Hi-C matrix on haplotype 1 of the LabAsex assembly for 137× coverage Hi-C data from LabAsex (bottom triangle) and 113× coverage Hi-C data from MenAsex (top triangle), highlighting the shared structural variation. Pink boxes outline off-diagonal signals shown in detail in (**g**). **g** Details of the Hi-C signal of (1) the Chr1/3 translocation, (2) the Chr1 inversion, and (3) the Chr2 inversion. In all cases, the Hi-C signal of LabAsex and MenAsex closely match. The signal was qualitatively identical across three clonal lines derived from Menorca individuals (Supplementary Note 3). Boxplots indicate median and interquartile range (IQR). Whiskers extend to 1.5 × IQR. Source data are provided as a Source data file.

One plausible explanation for the unexpectedly low cost of asexuality in *S. mediterranea* could be rare events of sexualization and sexual reproduction within wild asexual populations^61,62^. In fact, a case of sexualization of asexual *S. mediterranea* has been reported^63^ and seasonal switching between fissiparous and sexual reproduction is common in planarian species, including the sister genus *Dugesia*^64,65^. To test for the possibility of rare sex in the wild MenAsex population, we analyzed linkage disequilibrium (LD) as a function of genomic distance. The resulting LD decay curves can reveal recombination events in the form of breakdown of the associations between distant genetic variants^66^. While the exact shape of the LD curves can be influenced by selection, demography, and drift^67,68^, asexual populations without recombination are expected to display high LD regardless of distance^66^, but rapid distance-dependent decline can be expected in recombining sexual populations (Fig. 5a). Consistent with this prediction, the LD decay pattern in MenAsex shows little distance-dependent decay, supporting strict asexuality, while LD decay in SarSex is steep as expected in a sexual population (Fig. 5b). Furthermore, the SarSex population exhibited a higher inbreeding coefficient indicative of high inbreeding (Fig. 5c). Therefore, the MenAsex population is likely truly asexual and unlikely to undergo periodic events of sexualization.

A second plausible explanation for the apparent low genomic costs of asexuality in *S. mediterranea* could be a more recent origin of the asexual strain than the previous estimate of ~8.4 Ma, which was based on a two-step dating approach, with the initial calibration point based on the biogeographical split between the African and South American continents^39^. Instead of a biogeographical calibration, we used a strict molecular clock to estimate the divergence time between sexual and asexual strains. We estimated neutral divergence rates based on 1,961,630 four-fold degenerate sites in 10,556 single-copy genes and applied the formula *t* = *k*/*μ*, where *t* is the number of generations since divergence, *k* is the number of neutral substitutions per site, and *μ* is the mutation rate per site per generation. Using a pedigree-based estimate of *μ* = 1 × 10⁻⁸ for the LabSex strain^52^ and assuming 1–4 generations per year, we estimated the sexual-asexual divergence at ~0.4 Ma (0.16–0.64 Ma). Allowing two orders of magnitude variation in μ widened the estimate to 0.01–3.4 Ma (Fig. 5d). In addition, we estimated divergence using Pairwise Sequentially Markovian Coalescent modeling, which infers historical changes in effective population size from genome-wide polymorphism patterns. Using all genomes from the MenAsex and SarSex populations and again assuming *μ* = 1 × 10⁻⁸, we estimated the split at ~0.17 Ma (Fig. 5e). Finally, we estimated the median age of the last independent long terminal repeats (LTR) transposon insertion to be 0.21 Ma in the asexual genome and 0.42 Ma in the sexual genome, adding another line of evidence to the recent split (Supplementary Note 11). Therefore, three divergence time estimates independently suggest a relatively recent split between the asexual and sexual strains, which is also consistent with the k-means clustering analysis grouping MenAsex and LabAsex together (Supplementary Note 9).

### Impact of chromosomal rearrangements on genetic diversity

A recent sexual-asexual divergence is especially surprising given the broad geographic distribution of the asexual strain across the Balearic Islands and mainland Spain, and the genome-wide structural divergence between the lineages. Therefore, we explored if the observed chromosomal rearrangements were shared between MenAsex and LabAsex and if they might be linked to the origin of asexuality. To achieve this, we generated clonal isolines from individual MenAsex animals by cutting and regeneration and performed Hi-C sequencing on them. The Hi-C signal of four biological replicates from three isolines was highly similar, indicating a shared chromosome organization. When comparing MenAsex to LabAsex, we found that they shared the breakpoints for the Chr1/3 translocation, the Chr1 inversion, and the Chr2 inversion (Fig. 5f, g). This again confirms the remarkably high similarity between the Menorca and Barcelona strains and provides important confirmation that the many chromosomal rearrangements in the LabAsex assembly are not an artifact of laboratory culture.

**Fig. 6:**
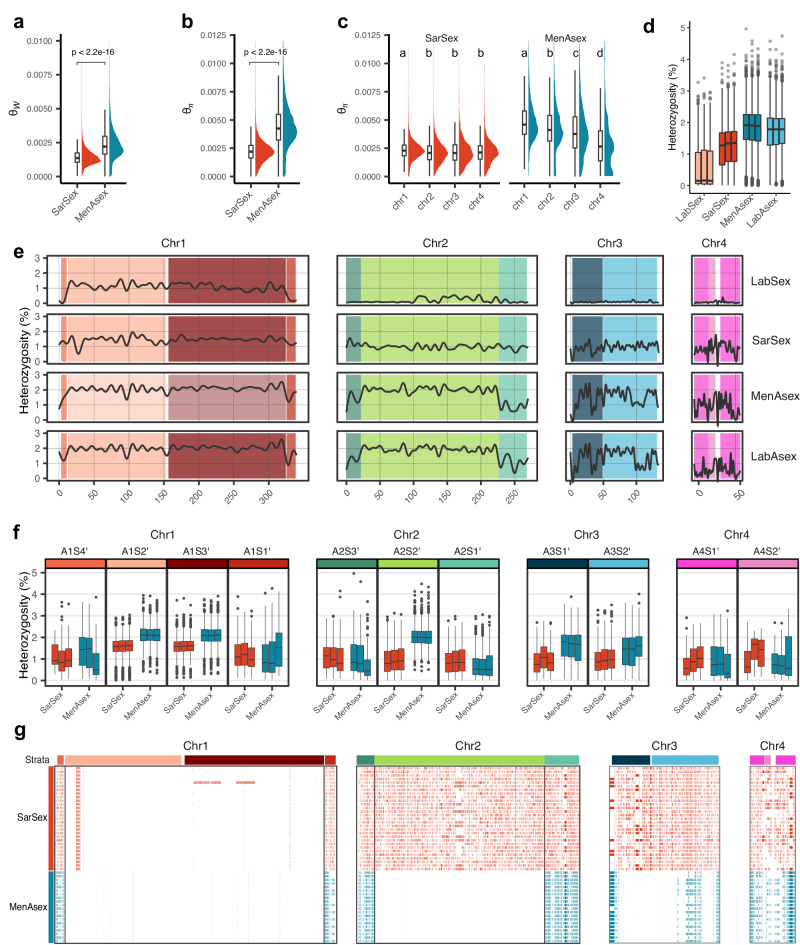
Genetic diversity and individual heterozygosity assessed using whole genome sequencing of an asexual population from Menorca (MenAsex) and a sexual population from Sardinia (SarSex). **a** Population mutation rate (*θ* = 4*N*_*e*_*μ*) estimated using Watterson’s estimator (*θ*_*W*_), which is based on the number of polymorphic sites. *θ*_*W*_ is significantly higher in MenAsex compared to SarSex (Wilcoxon rank-sum test). **b** Population mutation rate estimated using nucleotide diversity (*θ*_*π*_), which is based on the average number of pairwise differences between individuals. *θ*_*π*_ is significantly higher in MenAsex compared to SarSex (Wilcoxon rank-sum test). **c** Chromosome-specific *θ*_*π*_ estimates for SarSex and MenAsex populations. Different letters indicate significant differences between groups (pairwise Wilcoxon rank-sum tests with Bonferroni correction for multiple testing). **d** Percentage of heterozygous sites among all callable sites, calculated in 50 kb windows across three representative samples from each population (SarSex and MenAsex). Boxplots show the distribution of heterozygosity levels across the genome. **e** Average heterozygosity patterns from (**d**) displayed by chromosome (Chr1-4). **f** Heterozygosity levels across genomic strata in each chromosome. Boxplots show the distribution of heterozygosity within each defined genomic region. Colored bars above indicate genomic strata inferred via alignment to haplotype 2 of the LabAsex assembly (see Fig. 1). **g** Runs of homozygosity (ROH) in SarSex and MenAsex inferred on the sexual reference genome. Each row represents one individual. ROH is shown as colored segments. Colored bars above indicate genomic strata inferred via alignment to haplotype 2 of the LabAsex assembly (see Fig. 1). All analyses were performed using the sexual laboratory strain (LabSex) reference genome and were based on 29 SarSex and 20 MenAsex individuals in (**a**–**c**, **g**), and based on three individuals from each population in (**d**–**f**). Boxplots indicate the median and the interquartile range (IQR). Whiskers extend to 1.5 × IQR. All statistical tests were two-sided. Source data are provided as a Source data file.

To ask how the chromosomal rearrangements impact genetic diversity in the wild populations and to obtain hints at the mechanisms by which asexuality might have evolved, we inferred the population mutation rate *θ* for MenAsex and SarSex using the diversity estimator *θ*_*π*_ and the Watterson estimator *θ*_*W*_ across the genome in 50 kb windows. We found that for both estimators, genome-wide genetic diversity was significantly higher in MenAsex than in SarSex (Fig. 6a, b, both *p* values < 2.2e-16, Supplementary Note 9), consistent with the expected independent divergence of the two haplotypes in an asexual population. Interestingly, we found a difference in diversity estimates between chromosomes. In SarSex *θ*_*π*,_ diversity was significantly higher in Chr1 but not different between the other chromosomes, as expected if the Chr1 inversion would repress recombination (Fig. 6c). An even more striking pattern was observed in MenAsex: The highest diversity was observed on Chr1, followed by Chr2 and Chr3, which had similarly high diversity, and Chr4 showed substantially lower diversity, approaching the range observed in SarSex (Fig. 6c, similar results obtained using the *θ*_*W*_ estimator, Supplementary Note 9). Finally, we found that observed heterozygosity was high in MenAsex and LabAsex compared to SarSex, and the lowest in the inbred LabSex strain (Fig. 6d). This observation is consistent with the substantial structural variation on Chr1, Chr2, and Chr3. Taken together, these findings suggest a link between structural rearrangements and elevated genetic diversity, potentially driven by recombination suppression.

To investigate how individual heterozygosity was related to structural rearrangements, we examined heterozygosity along the chromosomes. We found that, across all strains, Chr1 consistently showed higher heterozygosity, whereas the other chromosomes were more homozygous in the highly inbred LabSex. Mapping the genomic strata from LabAsex revealed that regions of elevated heterozygosity in MenAsex and LabAsex aligned with the identified inversions and translocations (Fig. 6e). Specifically, MenAsex showed higher heterozygosity than SarSex within inversions on Chr1 (strata A1S2′ and A2S3′), the inversion in Chr2 (strata A2S2′), and Chr3 (A3S1′ and A3S2′) which was affected by the Chr1/3 translocation (Fig. 6f, Supplementary Note 9). These elevated levels of heterozygosity suggest suppression of recombination specifically in those regions, but recombination in other parts of the genome (i.e., signatures of sexuality). To understand this further, we inferred runs of homozygosity (ROH), which are a tell-tale sign of inbreeding, for SarSex and MenAsex. SarSex had almost no ROH in the Chr1 inversion but many ROH across all other parts of the genome, suggesting heavy inbreeding. In contrast, ROH in the MenAsex population were uniform between individuals and restricted to genomic regions not implicated in structural rearrangements (Fig. 6g), suggesting these ROH tracks were inherited from a sexual ancestor rather than the result of recent accumulation under asexuality (Fig. 6g). Overall, our results show that the population history of asexual *S. mediterranea* around the Mediterranean is intimately linked to the accumulation of chromosomal rearrangements and the associated suppression of recombination.

## Discussion

Here, we investigate the emergence of asexual reproduction in the planarian model species *S. mediterranea* from a comparative genomics perspective. The high-quality, haplotype-phased schMedA2 genome assembly of the LabAsex laboratory strain represents the first genome of an asexually reproducing planarian, enabling analysis of the genomic causes and consequences of asexuality. Surprisingly, our results reveal only weak traces of the predicted genomic consequences of asexuality, e.g., reduced purifying selection, changes in codon bias or transposon accumulation (Fig. 4b–j), despite obligate asexuality even in wild populations (Fig. 5b). Our results suggest asexual and sexual lineages split much more recently than the previously suggested ~8.36 Ma^39^ and reveal a history of chromosomal inversions and translocations that indicates a role of chromosomal dynamics in sexual system evolution.

The previous age estimate for the divergence between the asexual and sexual strains was based on a strict molecular clock applied to a 309 bp fragment of the COI gene, calibrated using the Gondwanaland split (~100 Ma) as a biogeographic event. Although fossil-based calibrations are generally more reliable, the absence of a useful fossil record in planarians makes this unfeasible^46^. With the availability of the LabAsex assembly and a pedigree-based estimation of the germline mutation rate in *S. mediterranea*^52^, we could reexamine this question using genome-wide data. A phylogenetic approach based on ~2 million four-fold degenerate sites yielded an age estimate of 0.4 Ma (Fig. 5d). Coalescence modeling, which infers divergence from patterns of genetic variation across both coding and non-coding regions, produced a slightly younger estimate of 0.17 Ma (Fig. 5e). A third approach, based on the neutral accumulation of transposon insertions, estimated divergence between 0.21 and 0.42 Ma (Supplementary Note 11). Finally, Guo et al. estimated the origin of the large inversion on chromosome 1 to be ~0.32 Ma^52^. We find this inversion to be shared between the sexual and asexual strains (Fig. 5f, g; Supplementary Note 3), further constraining the divergence time and contradicting earlier marker-based phylogenies that placed the asexual lineages as a sister group to all sexual lineages^39^. Together, these four orthogonal approaches converge on a much younger divergence between the asexual and sexual strains than previously thought, challenging the prevailing microplate tectonics model for the evolution and dispersal of *S. mediterranea* around the Mediterranean basin^38,39^.

**Fig. 7:**
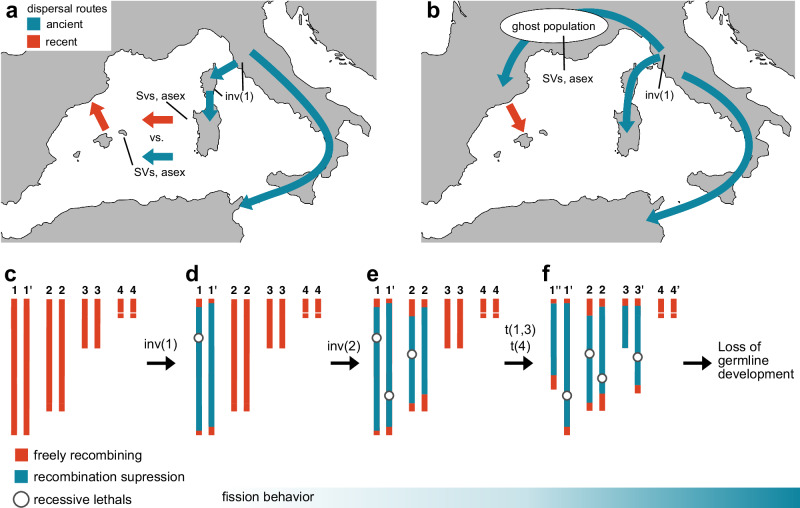
Biogeographical scenarios and evolutionary model for the origin and spread of asexuality in *S. mediterranea.* **a** Island scenario: The Chr1 inversion, inv(1), occurred on Corsica or the mainland, then other structural variants (SVs) occurred, followed by the origin of asexuality (asex). If dispersal to Menorca was recent (red arrow), then SVs and asexuality likely originated on Sardinia, but if dispersal was ancient (blue arrow), it is more likely that they originated on Menorca. **b** Ghost population scenario: inv(1) originated in a now-extinct ghost population on the Italian mainland. This population subsequently migrated along the Mediterranean coast and dispersed the Balearic Islands, carrying both the other SVs and asexual reproduction. **c**–**f** Proposed model of evolution of asexuality in *S. mediterranea*. **c** Ancestral genome without heteromorph structural variations and no recombination suppression. **d** Inversion inv(1) on Chr1 causing recombination suppression and the establishment of a lethal or balanced lethal system through the capture of deleterious alleles or de novo mutations. **e** Secondary inversion inv(2) on Chr2, extending recombination suppression to >60% of the genome. **f** Reciprocal translocation *t*(1,3) and additional rearrangements affect nearly the entire genome through heteromorphic changes. Homologous chromosome pairing becomes increasingly difficult and may require multivalent complex formation (Chr3′-Chr1′-Chr1”-Chr3′). During this evolutionary process, fission behavior evolved, enabling asexual reproduction through fission. This reproductive mode can co-occur with sexual reproduction, as observed in many *Dugesia* species, but eventually a mutation block to germline development arose, leading to obligate asexuality.

While an examination of regional paleo-climate records may provide further context for this revised timeline, our data are currently consistent with two alternative population history scenarios. In the “island scenario”, sexual strains bearing the Chr1 inversion first colonized Corsica and Sardinia, then dispersed to the Balearic Islands. There, factors such as small effective population sizes and inbreeding may have favored additional chromosomal rearrangements and the evolutionary emergence of asexuality, followed by a recent migration to the mainland (Fig. 7a). The “ghost population scenario,” in contrast, posits a now-extinct coastal ancestor that harbored the original inversion. Its descendants would have split into the Corsican/Sardinian and Spanish coastal populations, with the latter acquiring further rearrangements and asexuality before reaching the Balearic Islands (Fig. 7b). We favor the island scenario since the formerly known mainland asexual populations were found in artificial habitats (e.g., a fountain in Barcelona and irrigation canals in Girona), whereas the populations on Menorca were in more natural streams and wetlands. This makes anthropogenic dispersal, possibly via ornamental aquatic plants, from Menorca to the mainland more plausible than the reverse and thus favors the island scenario. Furthermore, the presence of both diploid and triploid populations on Menorca^37,40^, contrasting with exclusively diploid mainland populations, also suggests the origin of mainland populations from Menorca. Taken together, our results indicate that *S. mediterranea* has dispersed from Sardinia to the Balearic Islands and subsequently to the Spanish mainland, explaining the low genetic distance between asexual strains.

Irrespective of which of the dispersion scenarios applies, our results link the evolution of the asexual strain to structural chromosome rearrangements. It has long been known that the asexual strain carries a reciprocal translocation^36–38^. Our haplotype-phased genome assembly and FISH verifications demonstrate that this translocation is balanced with breakpoints located within two of the large tandem-repeat clusters on the *S. mediterranea* chromosomes (Figs. 2a, b and 3c). This is a significant finding, as the Chr1/3 translocation has previously been speculated to be causative of the lack of sexual development in the asexual strain. While both strains harbor *piwi*^*+*^*/nanos*^*+*^ primordial germ cells^56,69–71^, their development into gonads and accessory reproductive organs is blocked in the asexual strain, resulting in a complete absence of the reproductive system^56^. Our mapping of the Chr1/3 translocation breakpoints to gene deserts (Fig. 3a–c), along with the absence of gene loss or disruption in their vicinity and the lack of reproduction-related genes within 250 kb of the breakpoints (Supplementary Note 8), suggests that neither the Chr1/3 translocation nor the other structural alterations we could characterize are likely to be the primary cause of asexuality. Thus, the ultimate cause of the developmental reproductive system formation arrest is likely caused by changes elsewhere in the genome and thus unrelated to the pervasive chromosomal rearrangements.

Instead, our results suggest that these chromosomal rearrangements emerged in an ancestral sexual population and might have facilitated the eventual transition to asexuality. The Chr1 inversion that has been previously characterized in the sexual laboratory strain and that we find to be shared by the asexual strain is instructive here^43,52^. Inbreeding experiments show that the Chr1 inversion represses recombination, and therefore, we expect the inverted region to be shielded from heterozygosity loss due to inbreeding. We confirm this prediction in the wild sexual SarSex population, which exhibits reduced genetic diversity and frequent runs of homozygosity (ROH) in all regions of the genome not shielded by the Chr1 inversion (Fig. 6e–g), consistent with its high inbreeding coefficient (Fig. 5c). We observed a strikingly similar pattern in the wild asexual MenAsex population, where again we see high genetic diversity and minimal ROH specifically in rearranged regions but no differences outside them suggesting they were also shielded from inbreeding (Figs. 5c and 6g, a reduction of ROH on Chr1 was also previously observed in the LabAsex strain^72^). Crucially, alternative sources of high heterozygosity are insufficient to explain the observed pattern. If the pattern stemmed from the independent accumulation of mutations in both haplotypes—a phenomenon known as the Meselson effect^73,74^, which has been described in asexual rotifers and found in a few other asexual species^20,26,73,75,76^—we would expect to see relatively uniform divergence along the chromosomes. Similarly, hybridization of two populations with highly divergent chromosome structures, a common origin for many asexual lineages^77^, would also be inconsistent with the rearrangement-dependent pattern we observe. Instead, the pattern indicates the initial accumulation of heterozygosity in a sexually reproducing ancestor of the asexual MenAsex population and the asexual laboratory strain, with a subsequent loss of the germ line and reproductive organs.

Alternatively, the structural rearrangements observed in LabAsex and MenAsex, as well as the disruption of germline development, could have arisen via hybridization between highly diverged sexual populations. The reported presence of a triploid asexual population sharing the Chr1/3 translocation in Menorca^37,38,40^ could have arisen from meiotic disruption in hybrids between highly diverged diploid populations, leading to the production of unreduced (diploid) gametes that, upon crossing with a diploid sexual partner, would produce triploid offspring^78^. Under this model, the structural differences could have emerged rapidly during the hybridization event. However, as mentioned above, it remains unclear how the rearrangement-dependent pattern of heterozygosity would have arisen. While phylogenetic analyses including the triploid strains from both Sardinia and Menorca will be required to evaluate this scenario, our current results suggest the following model for the evolution of asexuality in *S. mediterranea*: The first step was the inversion on Chr1, which suppressed recombination across ~35% of the genome (Fig. 7c, d). In addition to shielding recessive deleterious alleles from inbreeding effects^78^, reduced recombination may have facilitated the maintenance of locally adapted haplotypes by limiting their breakdown through recombination with maladapted migrant alleles^78,79^. This effect may have been particularly relevant in geographically isolated island populations, where restricted gene flow and heterogeneous environmental conditions could favor the accumulation and persistence of locally beneficial allele combinations within rearranged chromosomal regions.

The subsequent inversion on Chr2 extended recombination suppression across most of that chromosome, thereby affecting ~60% of the genome (Fig. 7e). Finally, the Chr1/3 translocation extended recombination suppression to almost the entire genome (Fig. 7f). Although these rearrangements drastically limited recombination, the heterozygosity pattern shaped by inbreeding (see above) and the continued genetic propagation of the rearrangements suggest that sexual reproduction via egg-laying likely persisted through the later stages of this process. Indeed, several sexually reproducing populations of *Dugesia subtentaculata* in the sister genus carry distinct reciprocal translocations similar to the Chr1/3 translocation in *S. mediterranea*^26,80,81^, and flatworms are generally known to reproduce sexually despite varying ploidy^61^, mixoploidy^82,83^, and karyotype instability^84–86^. The last stage in the evolution of asexuality in *S. mediterranea* was the mutation(s) that abolished reproductive system development and enabled fissiparous reproduction. While the identification of the causative mutation(s) is an important objective for further studies, the drivers for the transition to behavioral asexuality likely included substantial fertility reductions caused by the preceding inversions and translocations. Specifically, the chromosomal rearrangements may have given rise to balanced-lethality systems^87^, as is likely present on Chr1, since homozygous Chr1/Chr1 or Chr1′/Chr1′ embryos in *S. mediterranea* are rarely viable^43^. The Chr1/3 translocation may also have necessitated a modified meiotic chromosome pairing mechanism, such as the multivalent complexes in primroses, which reduce gamete viability^88^. In addition, the block of reproductive system development likely removed important costs of sexual reproduction (e.g., metabolic costs of yolk/gamete formation; behavioral costs of mating), while also providing a dispersal advantage by offering reproductive assurance^89^. Taken together, our model represents one interpretation consistent with the data, in which adaptive chromosomal rearrangements progressively undermined the efficiency and genomic benefits of sex, thereby facilitating the ultimate transition to fissiparous asexuality to remove its associated costs.

Our data also provide a new perspective on the interpretation of the large inversion on Chr1 in sexual *S. mediterranea* from Corsica and Sardinia. Previous research described this inversion—with its recombination suppression and haplotype-biased expression of some reproduction-related genes—as a “sex-primed autosome” potentially evolving toward a sex chromosome^52^. While this remains a plausible hypothesis, it is important to note that separate sexes are extremely rare among planarians, possibly occurring in only two of many hundred species worldwide (*Sabussowia dioica* and *Cercyra teisseri*^90^). Our finding that the asexual strain also carries the Chr1 inversion, in addition to independently acquired similar recombination-impairing structural changes, suggests an alternative interpretation: rather than constituting a sex chromosome precursor, the Chr1 inversion may represent an early step in the evolutionary trajectory toward asexuality, as outlined in our model (Fig. 7c–f). Consequently, features resembling a nascent sex chromosome—specifically, recombination suppression preceding a sex-determining locus—might instead reflect a genomic predisposition toward asexuality.

The final question remains: How can *S. mediterranea* tolerate the costs of asexuality and reduced recombination, and more broadly, why are asexual reproductive strategies so common among planarians? On the one hand, the lack of strong signatures of reduced selection, even among fast-evolving reproduction-related genes^57,91,92^, could simply reflect the relatively recent origin of asexuality in these lineages. Under this scenario, the asexual *S. mediterranea* strain and other fissiparous planarian strains represent evolutionary dead ends that arise sporadically within parental sexual populations, compete and often outcompete their parental strains due to their reproductive advantages, but ultimately succumb to the cumulative effects of mutation load and other long-term disadvantages inherent to asexuality. On the other hand, the lack of mutational meltdown in our data and the abundance of asexually reproducing strains and species might indicate the existence of particularly effective means of purging deleterious mutations in flatworms. Somatic selection amongst the abundant pluripotent adult stem cells (neoblasts) as the only division-competent somatic cell type in planarians^29^ could provide one such mechanism. Early theoretical work on mutational load under vegetative reproduction predicted that the load is always at least as high as under sexual reproduction and often substantially higher^93^. Subsequent models, however, have shown that incorporating somatic selection can lead to efficient purging of deleterious mutations^25,27^. Tentative evidence for gene conversion in *S. mediterranea* supports the possibility that somatic purging mechanisms may operate in planarians^94^; however, such mechanisms would likely require either strong intra-individual selection or a small cell-population bottleneck to be effective^95,96^. Clonal dynamics amongst planarian neoblasts and the dual nature of fissiparous reproduction—as both a mechanism for transmitting somatic mutations to the next generation and a bottleneck that sub-samples the stem cell pool during each fission—create intriguing opportunities for both the purging of deleterious mutations and ongoing genomic evolution, even in obligately fissiparous planarians^24,25,27,97,98^. Therefore, key questions for future research include the extent of intra-animal mosaicism, intra-animal clonal dynamics, and bottlenecking effects of fission/regeneration^49^. In addition, population genomics of fissiparous planarian strains and species will be informative for comparisons with pioneering work on clonal evolution in seagrass and corals^95–97^. The genomic resources that we provide lay the foundation for addressing the mechanistic and evolutionary correlates of fissiparous asexuality in planarians.

## Methods

### Biological samples

Asexual *Schmidtea mediterranea* were collected from a stream along Rafal Colom Road, Menorca, Spain (39.9032, 4.2330) in May 2022. Sexual *S. mediterranea* were collected from the Temo River, Sardinia, Italy (40.3987, 8.5595) in April 2024. The asexual strain used for genome sequencing, genome assembly, and RNA-seq experiments was the long-term laboratory strain commonly used by most research groups, referred to as CIW4 (internal ID: GOE00071), which we here call LabAsex. For shotgun whole-genome sequencing and RNA-seq experiments using sexual worms from the lab, we used the previously established genome strain S2F18^47,48^ (internal ID: GOE00500), which we here refer to as LabSex. S2F18 derives from the S2F2 line originally collected in Sardinia and subsequently inbred through 18 rounds of self-fertilization^43,47^.

### HiFi sequencing

High molecular weight DNA for HiFi sequencing was extracted as previously described^48^. In brief, planarian mucus was stripped using a buffered 0.5% (w/v) N-acetyl-L-cysteine (NAC) solution for 10 min, followed by 30 min of lysis in ice-cold guanidinium thiocyanate buffer (GTC). Lysates underwent phase separation via phenol/chloroform/isoamyl alcohol extraction (25:24:1 ratio), followed by NaCl-induced clarification and isopropanol precipitation. DNA was washed with 70% ethanol and dissolved in TE buffer overnight at 4 °C. Post-extraction, DNA was further purified using a CTAB-based protocol and treated with RNase A. To remove residual contaminants, streptomycin precipitation was performed, followed by PEG/NaCl washing. Finally, DNA was resuspended in pre-dialysis buffer and dialyzed for 4–6 h using 0.1 μm pore-size membranes. The integrity and yield of the extracted DNA were assessed by pulse field gel electrophoresis (Pippin Pulse, Sage Science) and fluorometric quantification (Qubit, Thermo Fisher).

High-molecular-weight genomic DNA was processed for HiFi sequencing using the low-input SMRTbell Express Template Prep Kit 2.0 (PacBio), as previously described^48^. Briefly, DNA was sheared to 14–22 kb using the MegaRuptor (Diagenode), and 12–18 μg of sheared DNA was used for library construction. Size selection was performed using BluePippin™. Library preparation and sequencing followed PacBio’s standard protocols using the Sequel® II Binding Kit 2.2 and Sequencing Kit 2.0, with sequencing carried out on SMRT® Cell 8M chips on a Sequel® II system.

### Hi-C sequencing

To obtain the 1 million crosslinked nuclei used for Hi-C library preparation, we prepared 2 × 100 animals from our LabAsex laboratory population and 3 × 100 animals from three separate clonal lines we generated from single specimens collected from Menorca (GOE00553 56, GOE00553 9a, GOE00553 9f). Hi-C sequencing was performed as previously described^48^. Briefly, nuclei were isolated using a Dounce tissue grinder followed by chromatin conformation capture using the ARIMA-Hi-C High Coverage Kit (Article Nr. A101030-ARI), and Illumina library preparation using the Kapa Hyper Prep kit. The libraries were sequenced using 2 × 150 bp cycles on an Illumina NovaSeq 6000 at the DRESDEN Concept Genome Center, Dresden.

### Hi-C processing

We mapped all Hi-C sequencing reads to haplotype 1 of the LabSex (schMedS3) and LabAsex (schMedA2) assembly using the Arima SV pipeline (v: 1.3, https://github.com/ArimaGenomics/Arima-SV-Pipeline). For each genome, the necessary digested genome files were produced using Juicer (v: 1.6^99^, “generate_site_positions.py Arima”) and HiCUP (v: 0.8.0^100^, “hicup_digester –arima”). Since automatic detection of structural variations with the state-of-the-art tools relies on the availability of a normalized map of background interactions, which are not available for *S. mediterranea*, we ran the pipeline without the Hi-C Breakfinder module (“Arima-SV-Pipeline-v1.3.sh -W 1 -B 0 -J 1 -H 0”). Next, we applied the Knight-Ruiz matrix balancing to the interaction map of all datasets (LabAsex, LabSex, and MenAsex) using HiGlass^101^ and inspected them by locking resolution and zoom level between datasets and manually searching for off-diagonal signals that were exclusive to the sexual or asexual strains.

### Phased de novo assembly

PacBio HiFi and Hi-C reads were used to assemble phased contigs with hifiasm v0.7 with default settings (v0.7^102^). Next, Hi-C reads were mapped to the contigs with bwa mem (v0.7.17) with parameter “-5SP -T0.” Reads with mapping quality no less than 10 (-q 10) were further utilized to scaffold the contigs from each haplotype by SALSA (v2^103^) following the hic-pipeline (https://github.com/esrice/hic-pipeline). Scaffolding errors were then manually curated based on the interaction frequency indicated by the intensity of Hi-C signals.

### Identification of genomic strata and breakpoints

We used Minimap2 (v2.28^104^) to align both haplotypes of the LabSex and LabAsex assemblies in all pairwise combinations (“-x asm5 -c –eqx --secondary=no”). Then we first identified the genomic strata, defined as sections of the genome with a shared rearrangement history, via the alignment of haplotype 1 and 2 of the LabAsex genome. For this, we parsed the whole-genome alignments using SyRI (v.1.6^105^) run with default parameters and visualized and manually inspected all indicated duplications, translocations, and syntenic alignments. Since we saw that the large parent elements of the alignments explained the observed karyological differences and agreed with the Hi-C signal (see above), we labeled them as genomic strata, naming them based on their location on haplotype 1 (which is more similar to the sexual genome) (AXSY, where A = Asexual, followed by chromosome number S=strata, followed by strata number). We named the strata on haplotype 2 based on their homology with haplotype 1 and applied a to indicate that it is derived (e.g., A1S1 and A1S1′). The same process was used to define strata in the sexual genome. Then we used the other alignments to lift over the strata in the asexual haplotype 2, which represents the rearrangement from one haplotype to both haplotypes of the sexual assembly. Again, care was taken to manually inspect and select the appropriate liftovers to avoid greedy assignment of regions due to short alignments that could represent duplications due to transposable elements or translocations. Breakpoints between strata were double-checked with HiC, dotplots of the alignments, minimap2 alignments generated using R with functions adapted from dotPlotly (https://github.com/tpoorten/dotPlotly), and inspection of LASTZ syntentic chains in our local instance of the UCSC genome browser. Finally, for the Chr1/3 translocation breakpoint, we compared the similarity of the repetitive region using StainedGlass with a segment size of 2000bp.

### Genome QC

k-mer spectra of short-read or HiFi reads were constructed using meryl (v1.4.1) with k-mer length of 20 and 21, respectively. We assessed the genome phasing and quality using merqury (v1.3^106^) in diploid mode, and performed reference-free quality control and heterozygosity estimation of the HiFi reads using GenomeScope2.0^107^. We assessed genome and transcriptome completeness using BUSCO (v5.3.2^108^) with the metazoan_odb10 reference set, which contains 954 genes. Transcriptome completeness was quantified using the predicted coding sequences.

### Genome annotation

We improved upon our previously published gene annotation approach, combining Nanopore direct RNA and cDNA long-reads, Illumina cDNA short-reads, and 3P-seq of transcription termination sites^109^. For this purpose, we employed long-read RNA-sequencing runs from Ivanković et al.^48^ (SRX23002382-SRX23002387), as well as newly sequenced data (see Supplementary Data 15). RNA extraction, Oxford Nanopore cDNA library preparation, and hybrid genome annotation followed established protocols^48^. We then extended the strategy to include annotation liftover between haplotypes to avoid inferring haplotype-specific gene loss due to annotation errors. Additionally, we used homology information via alignment of the dd_smed_v6 reference transcriptome to add coding DNA sequence models (CDS) for short proteins and to detect potential chimeric gene models, as we had previously observed a small number of chimeras in the LabSex annotation. To facilitate reference-based inference of gene loss, we also applied these improvements to our previous annotation of the sexual assembly. For details, see Supplementary Note 2.

### Functional annotation

To annotate the transcriptome for potential function, we used eggNOG-mapper (v2.1.10^110^) with the options “--sensmode ultra-sensitive --report_orthologs -m diamond --dmnd_iterate yes --pfam_ralign realign”, and the eggNOG 5.0 database. We also ran InterProScan (v5.54_87.0) with the options “-goterms --pathways” using all available databases. Additionally, we used DIAMOND with the options “--very-sensitive --evalue 1e-5” to search against published protein sequences for *Schmidtea mediterranea* from NCBI, retrieved via the efetch utility using the query “Schmidtea mediterranea[Organism] AND cds[Feature key]”.

### De novo repeat discovery and annotation

Tandem repeats and transposable elements were annotated using the Extensive de novo TE Annotator (EDTA) workflow (v2.1.0^111^) with settings “--species others --step all --sensitive 0 -anno 1” in combination with the repeat library we previously generated for the LabSex strain^48^. To identify repetitive sequences that were not annotated using the other approaches, we estimated the genome mappability using genmap (v1.3.0-2, ref. ^112^). We used a kmer length of 150 and an error rate of 2. The mappability score is calculated as 1/*N*, where *N* represents how often a kmer can be mapped to the genome.

### Chromosome fluorescence in situ hybridization (FISH)

We selected two highly abundant tandem repeats based on their genomic distribution—one of which is located at the Chr1/3 translocation breakpoint—and because in silico predictions suggested that the observed structural variation would lead to differences in hybridization signal. Specifically, we selected a 158 bp (158mer) and a 159 bp (159mer) repeat (Supplementary Data 1) and designed directly labeled probes for oligo-FISH. The 158mer showed partial similarity to a sequence identified by Guo et al.^52^, and both the 158mer and the sequence from Guo et al. match a short probe identified as located close to the centromere of Chr2 by Chretien^113^ (Supplementary Note 5). We also selected putative centromeric repeats based on their position in the genome assembly, identifying them as the only highly abundant tandem repeats in the expected centromeric region, and designed PCR primers for their amplification. Oligo-FISH using the 158mer and 159mer was performed on both sexual (LabSex) and asexual (LabAsex) strains, while standard FISH was conducted only on LabAsex. Chromosome spreads were prepared from regenerating tail fragments, and FISH was carried out using established protocols^85^. Details on probe generation, chromosome preparation, and hybridization conditions are provided in Supplementary Information: Chromosome FISH, and all primers and oligo DNA probes used in this study are listed in Supplementary Data 2.

Chromosome microimages were captured using a CCD camera mounted on an Axioplan 2 compound microscope (Zeiss, Germany) equipped with filter cubes #49, #10, and #15, and controlled via ISIS4 software (METASystems GmbH, Germany) at the Center for Microscopic Analysis of Biological Objects, SB RAS (Novosibirsk, Russia).

### RNA interference experiment

To identify reproduction-related genes, we compared gene expression profiles between sexual (LabSex) and asexual (LabAsex) wild-type animals, and between *eGFP* and *ophis* RNAi treatments. Sexual wild-type and RNAi animals underwent a standard regeneration assay. Animals were fed twice with liver paste, which was supplemented with 2 µg/µl of dsRNA in the RNAi treatments. Then heads were amputated anterior to the ovaries but posterior to the eyes, to ensure removal of the reproductive system. After complete regeneration of the head fragments, the animals were fed dsRNA-supplemented liver paste twice a week until control animals had laid cocoons and exhibited visible gonopores. Each treatment was replicated in five 9 cm petri dishes with ten animals each, ensuring balanced incubator distribution and monitoring of food intake. Animals were maintained in Montjuïc water supplemented with 5 μg/mL ciprofloxacin. LabAsex animals were maintained identically, and size-matched individuals were collected. Three animals per group were stained with DAPI to confirm the presence or ablation of the reproductive system.

### Whole-mount DAPI staining

Worms were processed similarly to the standard whole-mount in situ protocol^114^, omitting the staining with riboprobes. Briefly, worms were killed in 7.5% N-Acetyl-L-cysteine in 1X PBS for 5 min with agitation, rinsed with 4% formaldehyde in 0.5X PBSTx0.3, then fixed in fresh fixative for 45 min. After three washes in 1X PBSTx0.3, specimens were incubated in reduction solution at 37 °C for 10 min. Following three additional washes, samples were dehydrated through a methanol series and stored at −20 °C. For staining, specimens were rehydrated, bleached with 6% H_2_O_2_, PBSTx0.3 for 3 h on a light table, and treated with Proteinase K (2 μg/ml) for 10-25 min. After post-fixation, samples were prepared for hybridization, incubated in hybridization buffer at 56 °C overnight, and subsequently washed. Specimens were then stained with DAPI (1:1000) overnight at 4 °C or for 2.5 h at room temperature, washed extensively, and mounted for imaging. Whole-mounts were imaged using an Olympus IX83 microscope equipped with a Yokogawa CSUW1-T2S Spinning Disk system and a Hamamatsu Orca Flash4.0 V3 camera. Images were acquired with an Olympus UPLXAPO 20× air objective (NA = 0.8), with the emission 447/50 filter and excitation at 405 nm.

### Double-stranded RNA production

For RNAi-mediated knock-downs, dsRNA for *ophis* (NCBI accession: KX018822.1, forward primer: ATTGTTAGGATATATTTTGAAACAATTGATG, reverse primer: TGCAGTATTCCATGCATGGC) and *eGFP* was synthesized in vitro and mixed with liver paste as described in Rhouhana et al.^115^. Briefly, linear DNA templates were produced by PCR with T7-AA18 and PR244 primers with pPRT4P-Smed-ophis or pPRT4P-eGFP plasmids as templates. DNA templates were purified and used in in vitro transcription reactions with the T7 RNA Polymerase (Thermo Fisher, EP0111). The dsRNA was then purified by NaCl / PEG-8000 precipitation, diluted to 8 µg/µl, mixed with liver paste (final concentration of 2 µg/µl), and stored at −80 °C until feeding.

### RNA extraction and sequencing

For each treatment, six samples were collected, each consisting of a single worm. Animals were lysed in TRIzol reagent (Invitrogen), and total RNA was extracted using the Direct-zol RNA MiniPrep Plus Kit (Zymo Research, Cat. No. R20700), following the protocol described in ref. ^116^. The libraries were sequenced to an approximate depth of 55 million reads per sample using 2 × 150 bp cycles on an Illumina NovaSeq 6000 at the DRESDEN Concept Genome Center, Dresden.

### Differential expression analysis

Raw reads were screened for contamination with Kraken2 and trimmed using Trimmomatic (v0.39, ref. ^117^) with parameters ILLUMINACLIP:[…]:2:30:10 LEADING:20 TRAILING:20 SLIDINGWINDOW:4:20 MINLEN:35. Trimmed reads were aligned to the sexual reference genome (GenBank accession: GCA_045838265.1) using STAR (v2.7.9a, ref. ^118^) in quantification mode to generate gene-level count matrices. Genes with low raw counts were filtered using the *filterByExpr* function in edgeR (v4.4.0), retaining genes with 5 counts in at least 6 samples and a minimum of 20 counts over all. Library size normalization was performed using the TMM method, and the *voom* transformation was applied to estimate mean-variance trends and generate precision weights. Differential expression analysis was conducted using limma (v3.62.1, ref. ^119^) with a linear model accounting for the three experimental conditions. The following contrasts were tested: *ophis* RNAi vs. asexual wild-type (O_vs_A), sexual control vs. asexual wild-type (S_vs_A), and sexual control vs. *ophis* RNAi (S_vs_O). Genes were considered differentially expressed if they had an absolute log2 fold change ≥2 and an adjusted *p* value < 0.01 (Benjamini-Hochberg correction). Volcano plots were generated using EnhancedVolcano (v1.24.0), with reproduction-related genes highlighted based on curated sexual marker annotations. All downstream analyses were performed in R (v4.4.2) using the tidyverse (v2.0.0) packages.

### Divergence time estimation using four-fold degenerate sites

Our aim was to estimate the divergence time between LabSex and LabAsex in the absence of fossil or geographic calibration points by approximating neutral divergence using four-fold degenerate sites from single-copy genes. These sites, while more conserved than truly neutral positions, offer the advantage of reliable homologous alignment. To infer orthologous genes, we leveraged the genome assemblies of the three species most closely related to *S. mediterranea*, which we recently published^48^. We used GENESPACE (v1.0.8^120^), which takes advantage of protein similarity via OrthoFinder(v2.5.4^121^) and DIAMOND (v2.0.14^122^) in combination with synteny information inferred using MCSanX (v1.0.0^123^). Then we extracted syntenic single-copy genes using a custom script.

For each single-copy gene, we extracted four-fold degenerate sites using degenotate.py (v1.3^124^) and then concatenated all sites across the single-copy genes into a supermatrix using AMAS^125^. Substitution rates were estimated using IQ-TREE (v2.3.6^126^) on the unpartitioned supermatrix using the GTR+F+I+G model. The cophenetic distance between LabSex and LabAsex was extracted and halved to obtain the maximum likelihood estimate of substitutions per site since LabSex and LabAsex diverged. Divergence time (*t*, in generations) was calculated using the equation *t* = *k*/*μ*, where *k* is the estimated neutral divergence and *μ* is the mutation rate. We used a pedigree-based estimate of *μ* = 1 × 10⁻⁸ mutations per site per generation from *S. mediterranea* as a baseline^52^, and varied this rate by one order of magnitude in either direction to explore the resulting range of divergence time estimates.

### Estimation of LTR insertion age

*Schmidtea* species have abundant and active LTR transposons^47,48^. We estimated the age of LTR insertions restricted to either the sexual or asexual genome to place an upper bound on their divergence. We used SubPhaser (v1.2.6^127^), which identifies LTR insertions and uses phylogenetics to estimate the divergence between them. We ran SubPhaser with default parameters but set the mutation rate to 1 × 10⁻⁸ (see above).

### non-synonymous/synonymous substitutions and codon usage

Since the available gene annotations for *Schmidtea mediterranea* include genes with low expression and/or repetitive content, we restricted our analyses to genes that passed the minimal expression filter described in the differential expression analysis section. Of the 10,560 single-copy genes inferred using GENESPACE (see above), 10,470 passed this filter and were used for analyses of non-synonymous to synonymous substitution rates (*d*_*N*_/*d*_*S*_) and codon usage. For each orthogroup, protein sequences were aligned using MAFFT (v 7.480^128^, “--globalpair --maxiterate 1000”), and corresponding gap-free codon alignment was generated using PAL2NAL (v14.1^129^, “-nogap”). We then used CODEML from the PAML package (v4.9^130^) to estimate all pairwise *d*_*N*_/*d*_*S*_ ratios (“runmode = −2, model = 0, fix_kappa = 0, fix_omega = 0”). To assess differences in purifying selection between the sexual and asexual strains, we focused on conserved genes (*d*_*N*_/*d*_*S*_ < 1) and compared *d*_*N*_/*d*_*S*_ values between LabSex and LabAsex relative to other *Schmidtea* species (e.g., LabSex vs. *S. polychroa* and LabAsex vs. *S. polychroa*). We also evaluated *d*_*S*_ between LabSex and LabAsex to ensure sufficient neutral divergence and applied a filter of *d*_*S*_ ≥ 0.1 for a subset of genes.

Codon usage was characterized by estimating the effective number of codons using codonw (v1.4.4^131^, “-all_indices”) and the codon deviation coefficient using CAT (v1.3^132^, “-b 10000 -c 1”).

Paired, two-sided permutation tests were performed for all parameters to assess differences between LabSex and LabAsex using the *oneway_test* function from the coin package (v1.4-3) with rank transformation. The test statistic and *p* value were calculated based on 1,000,000 resampling iterations. Cohen’s *d* effect size was calculated using the effsize package (v0.8.1), and mean differences with 95% confidence intervals were estimated via bootstrap resampling using the boot package (v1.3-31). In the *d*_*N*_/*d*_*S*_ analysis, we corrected for multiple testing using the Benjamini-Hochberg procedure, setting the false discovery rate to 0.05. All statistical analyses were conducted in R (v4.4.2) using tidyverse packages (v2.0.0).

### Gene loss analysis

The TOGA^54^ gene annotation pipeline was used to identify genes lost in the asexual LabAsex assembly. TOGA was run with default parameters and the improved LabSex assembly annotation (see above) as reference. We identified genes that were flagged as lost or potentially lost in both haplotypes of LabAsex. Additionally, we annotated a gene as conserved if it was present in at least 2/3 of the other Schmidtea assemblies.

### COI barcoding and short-read sequencing

DNA for barcoding and whole-genome sequencing was extracted as described for HiFi sequencing, but without the post-extraction cleanup steps. To confirm species identity, we PCR-amplified an ~880 bp fragment of the mitochondrial cytochrome c oxidase subunit I (COI) using the MVCOI900 degenerate primer pair^133^. Each 50 µl reaction contained 2 µl genomic DNA (1–20 ng), 5 µl Taq Standard Buffer, 2 µl MgCl₂ (25 mM), 1 µl dNTPs (10 mM), 1.5 µl of each primer (10 µM), 0.25 µl Taq polymerase (NEB M0273S), and 36.75 µl nuclease-free water. Cycling conditions were: 95 °C for 5 min; 30 cycles of 95 °C for 30 s, 50 °C for 30 s, and 68 °C for 1 min; followed by a final extension at 68 °C for 3 min. Products were purified with the QIAquick PCR Purification Kit (Cat. No. 28104) and sequenced in both directions; complementary reads were assembled in Geneious and translated to confirm reading frame integrity. COI sequences were compared against the transcriptomes of LabSex and LabAsex, and the COI haplotype diversity of ref. ^39^. We then removed redundant identical sequences, aligned them using MAFFT (v7.480^128^, “--globalpair --maxiterate 1000”), identified HKY+F+I as the best fitting substitution model using ModelFinder^134^, and then inferred a maximum likelihood phylogeny using PHYML (v2.2.4^135^) with 100 non-parametric bootstraps to determine branch support.

Based on these barcoding results, we selected samples for whole-genome sequencing (WGS). For the Menorca sample, DNA from 20 individuals was purified using the Zymo Research Genomic DNA Clean kit. Libraries were prepared with the Illumina Nextera DNA Flex Library Prep Kit and sequenced using 2 × 150 bp cycles on an Illumina NovaSeq 6000 at the Leibniz Institute of Plant Genetics and Crop Plant Research, Gatersleben. For the Sardinia samples, DNA extracts from 29 individuals were prepared with the Illumina Nextera DNA Flex Library Prep Kit and sequenced using 2 × 150 bp cycles on an Illumina NovaSeq 6000 at Genewiz Europe, Leipzig. The three LabAsex and three LabSex samples were prepared with the KAPA HyperPlus Library Prep Kit and sequenced using 2 × 100 bp cycles on an Illumina NovaSeq 6000 at the DRESDEN Concept Genome Center, Dresden.

### Variant calling

Raw sequencing reads were pre-processed as described for the RNA-seq, aligned to the reference genome using BWA-MEM, and processed following the GATK Best Practices workflow^136,137^. We employed joint genotyping for the *π*_*N*_/*π*_*S*_ analysis and per-sample genotyping to assess observed heterozygosity. For joint genotyping, per-sample GVCFs were generated, consolidated by scaffold using GenomicsDBImport, and jointly genotyped with GenotypeGVCFs. For per-sample genotyping, per-sample GVCF files were called using GenotypeGVCFs “--all-sites” option, resulting in detailed calls for each genomic position.

### Heterozygosity

We filtered the per-sample GVCF file using BCFtools by excluding sites with missing genotypes (GT = “mis”), high strand bias (FS > 30), low depth (FMT/DP ≤ 10), or indels (TYPE = “indel”). A custom script based on the R package GenomicRanges was used to calculate the number of variant sites and the total number of callable sites within 100 kb sliding windows, using a 50 kb step size. Windows with fewer than 50,000 callable positions were excluded from further analysis. Observed heterozygosity was calculated as the ratio of variant sites to total callable sites within each retained window.

### *π*_*N*_/*π*_*S*_

Nucleotide diversity parameters (*π*_*N*_ and *π*_*S*_) were calculated for both sexual (SarSex) and asexual (MenAsex) populations using SNPGenie (v1.0^138^). Biallelic SNPs found in protein-coding exons were extracted from the joint GVCF using BCFtools (–v snps –T exons.bed –AA –a) and filtered to remove sites with high strand bias (FS > 30) or low depth (FMT/DP ≤ 10). To identify differences in purifying selection, only those genes with πS > 0.001 and *π*_*N*_/*π*_S_ < 1 were retained.

### PCA and admixture

We used 55 samples for genotype calling, consisting of MenAsex (20), SarSex (29), LabSex (3), and LabAsex (3). We used ANGSD (v: 0.940^139^) to call genotype likelihoods with stringent filtering criteria (“-SNP_pval 1e-6 -minMapQ 20 -minQ 20 -uniqueOnly 1 -remove_bads 1 -only_proper_pairs 1 -trim 0 -C 50 -baq 1 -setMinDepthInd 10 -minInd 42”) to maintain the high quality of the data. The called genotype likelihoods were then used as input for PCAngsd (v: 1.10 ^140^) to perform PCA and NgsAdmix^141^ for estimating individual admixture proportions. To determine the best K (expected number of genetic clusters or ancestral populations) for admixture proportions, 10 replicates per K were run in NgsAdmix, and log-likelihood values were extracted. Best K was identified by Evanno’s method^142^ implemented in CLUMPAK^143^.

### Inbreeding coefficients

The BAM alignments of Menorca and Sardinia were used separately to call genotype likelihoods using ANGSD with additional flags (-doMajorMinor 1 -doMaf 1 -skipTriallelic 1 -doGlf 3) to generate Beagle files for both populations. ngsF-HMM^144^, a tool to estimate inbreeding coefficients (*F*_IS_) and IBD tracts efficiently from low coverage sequencing data, was used to calculate individual genome-based *F*_IS_ for both populations.

### Linkage disequilibrium

The BAM alignments of Menorca and Sardinia were used separately to call genotype likelihoods using ANGSD, similar to what was mentioned above, except they were only restricted to the sites within coding regions. The number of sites called was then prioritized for estimating pairwise LD values using ngsLD^145^ with additional flags—-probs to specify input type and—-max_kb_dist 1000 to specify the distance required to consider the sites for LD calculations. The results were then subsetted randomly to a smaller portion to plot the exponential LD curves for both populations.

### SMC++

The BCFtools version 1.19^146^ was used to call variants using all Menorca (20) and Sardinia (29) individuals with filtering (-C50 -q30 -Q30) to retain better quality called variants in the resultant vcf file. SMC++^147^ vcf2smc module was used to convert the vcf data into smc++ input data. SMC++ estimate was used to calculate effective population size (Ne) estimates for both populations separately using a mutation rate of 1e^−08^ per generation, followed by an SMC++ plot to visualize the temporal population size trends for each population. The SMC++ split module was used using both populations together to calculate the estimate of joint demography, which gives an indication of the split time between the two populations.

### Genetic diversity and differentiation

The site frequency spectrum (SFS) was estimated for Menorca and Sardinia populations by obtaining genotype likelihoods using ANGSD. To maintain high-quality variants, we used uniquely mapped and properly mate-mapped reads with mapping quality adjustment (“-C 50”), adjusting q-scores around indels (“-baq 1”), minimum mapping quality of 20 (“-minMapQ 20”), minimum base quality of 20 (“-minQ 20”), at least 75% of individuals considered (“-minInd Nx0.75”), depth of at least 10 bases at each site in each individual (“-setMinDepthInd 10”). The obtained genotype likelihoods in the form of sample allele frequency (SAF) were then used to estimate SFS using the realSFS module of ANGSD. The theta estimations were obtained using the saf2theta module of ANGSD using SAF. SFS and theta estimations were further used to get estimates of diversity over 50 kb non-overlapping windows using thetastat module of ANGSD. The mean of Watterson’s estimator (*θ*_*w*_), Pairwise nucleotide diversity (*θ*_*π*_), and Tajima’s *D* of the 50 kb windows, normalized by the number of sites in each window for each population, was calculated for all chromosomes.

### Reporting summary

Further information on research design is available in the Nature Portfolio Reporting Summary linked to this article.

## Supplementary information

Supplementary Information

Transparent Peer Review file

Reporting Summary

Supplementary Dataset 1

Supplementary Dataset 2

Supplementary Dataset 3

Supplementary Dataset 4

Supplementary Dataset 5

Supplementary Dataset 6

Supplementary Dataset 7

Supplementary Dataset 8

Supplementary Dataset 9

Supplementary Dataset 10

Supplementary Dataset 11

Supplementary Dataset 12

Supplementary Dataset 13

Supplementary Dataset 14

Supplementary Dataset 15

## Source data

Source data

## Data Availability

All sequencing data generated for this study have been deposited in the National Center for Biotechnology Information (NCBI) database. The schMedA2 LabAsex genome assembly is available under GenBank accessions JBMAJT000000000 (haplotype 1) and JBMAJU000000000 (haplotype 2). The schMedA2 genome gene and repeat annotation are available from Zenodo under accession code 18415144. Data used for genome assembly and annotation are available under BioProject accession PRJNA1289722. RNA-seq data used for the identification of reproduction-related genes are available under BioProject accession PRJNA1289391. Population genomics data for *Schmidtea mediterranea* are available under BioProject accession PRJNA1287507. Source data are provided with this paper.

## Code availability

Custom code and scripts used for data processing, analysis, and visualization are available on GitHub at https://github.com/Jeremias-Brand/SmedAsex and have been archived in Zenodo under accession code 18433485.
