# Supplementary material for: Stepwise emergence of recombination suppression precedes fissiparous asexuality in the planarian Schmidtea mediterranea
Source: Nat Commun. 2026 Jun 26;17:5588. doi: 10.1038/s41467-026-74605-9 (PMC13309565; doi:10.1038/s41467-026-74605-9)
Supplement: Supplementary file 1 — Supplementary Information [file 41467_2026_74605_MOESM1_ESM.pdf]

Stepwise emergence of recombination suppression precedes fissiparous asexuality in the planarian *Schmidtea mediterranea*

Jeremias N. Brand*, Ajinkya Bharatraj Patil, Luca Pandolfini, Kira Zadesenets, Nikolay Rubtsov, Laura Robledillo, Meng Zhang, André Marques, Jochen C. Rink*

Table of Contents

Supplementary Note 1 | Genome Quality Control 2

Supplementary Note 2 | Gene Annotation 4

Supplementary Note 3 | Hi-C Analyses 8

Hi-C Quality Control 8
Hi-C of Chromosome 4 9
Hi-C Comparison of LabSex and LabAsex 10
Hi-C Menorca Isolines 13

Supplementary Note 4 | Karyology from the Literature 15

Supplementary Note 5 | Chromosome FISH 19

Generation of DNA probes specific to the predicted centromeric satellite DNA repeats 19
Chromosome Slide Preparation 20
Fluorescent *in situ* Hybridization 21
Quantification of FISH Signal in LabAsex and LabSex 21
FISH Images 23

Supplementary Note 6 | Synteny between LabSex and LabAsex 27

Supplementary Note 7 | Reproduction-related Genes 30

Power Calculation 30
Confirmation of RNAi via Phenotyping 30
Differential Gene Expression Analysis 32

Supplementary Note 8 | Gene Loss 38

Supplementary Note 9 | Population Genomics 41

Supplementary Note 10 | Dataset Intersection 50

Supplementary Note 11 | LTR Insertion Age 53

Supplementary Note 1 | Genome Quality Control

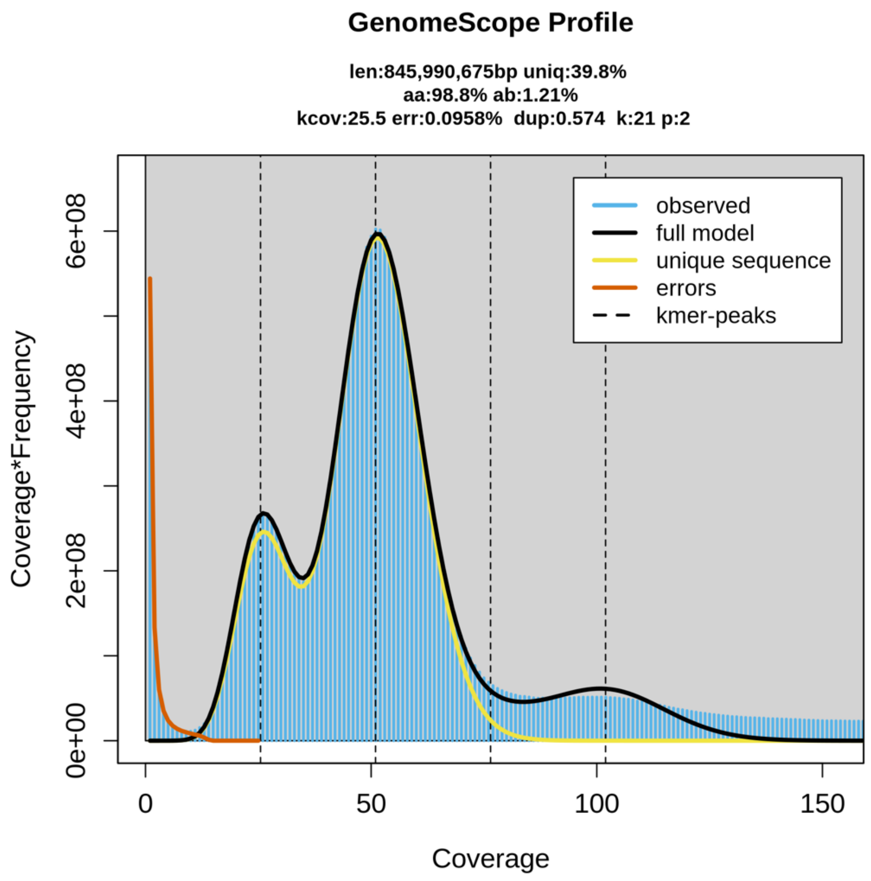

Supplementary Fig. 1 k-mer distribution in the asexual HiFi reads and the inferred genome size, and heterozygosity based on the model of GenomeScope 2.0.

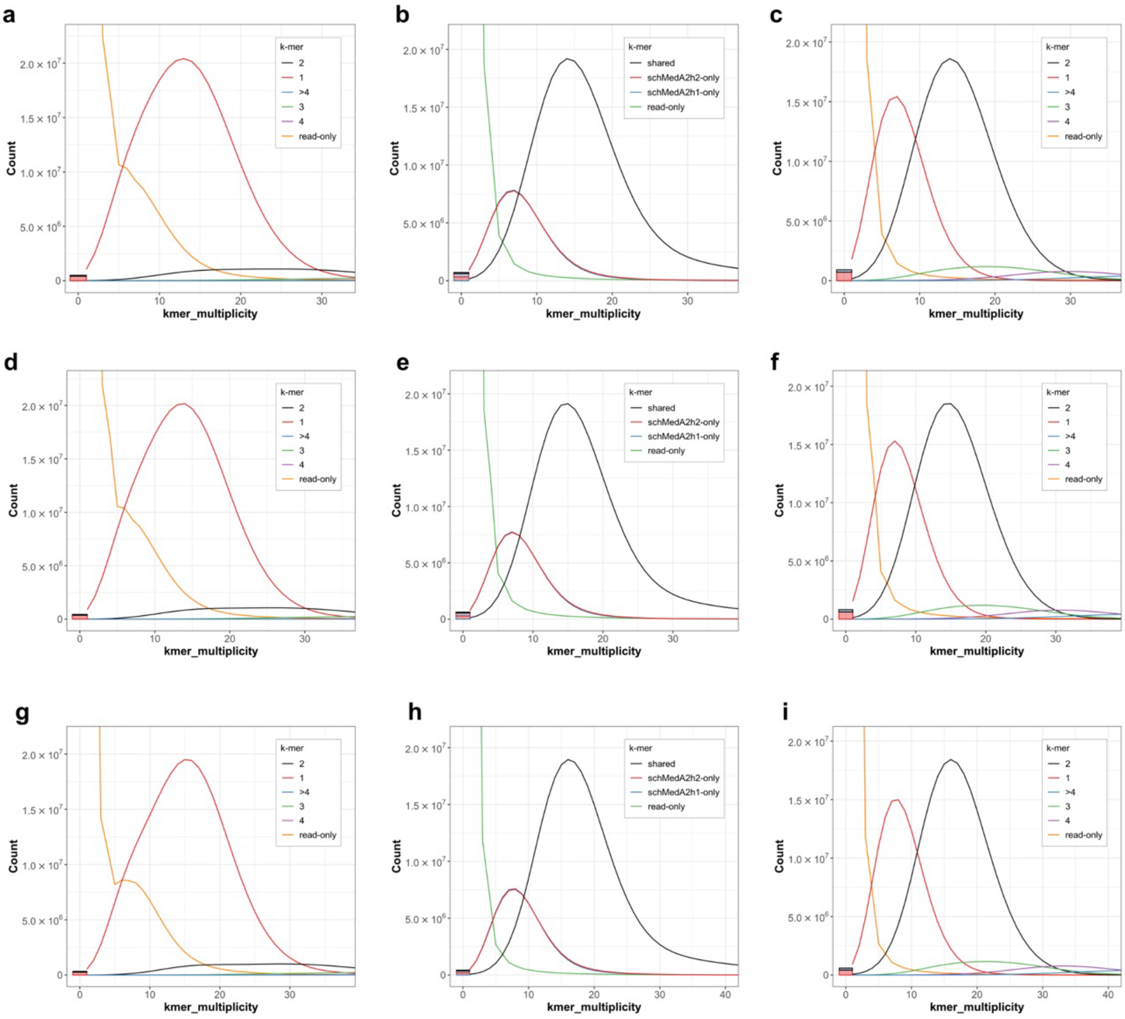

Supplementary Fig. 2 Merqury analysis of the LabAsex assembly (schMedA2) using three independent short read datasets generated from the genome strain. Rows are results from the three datasets (a-c: LabAsex_A, d-f: LabAsex_B,g-i: LabAsex_C, which are available in the NCBI repository under the following accession: PRJNA1289722). First column shows k-mer spectra for haplotype 1 clearly indicating a substantial proportion of k-mers with a high coverage occur only in the reads. Second column shows, that when considering both haplotypes these reads are divided between the assemblies for each haplotype, thus indicating good phasing efficiency. Finally, the last column shows the k-mer spectrum of the diploid assembly. We can see that the first peak (shown in red), which represents the haplotype specific k-mers is of a similar height to the second peak, which represents the shared diploid k-mers. Therefore, heterozygosity is high.

Supplementary Note 2 | Gene Annotation

For liftover, transcripts were aligned against the genome using minimap2 (v2.28-r1209[1],-cx splice:hq --cs -G 100k -L -u b -N 1), and CDS were predicted for both the existing annotation and the alignments. CDS were predicted using TransDecoder (v5.7.1) configured to detect short proteins (-m 40) and retain low-scoring models with homology to PFAM or UniRef90 databases as assessed using MMseqs2 (v16.747c6[2]). Next we collected extensive statistics on the liftovers using an all-vs-all sequence search of all inferred CDS using MMseqs2, assessed exon structure comparison using GFFcompare (v0.12.2[3]), assessed transcript coverage using minimap2, and collected statistics on splice junction coverage, expression, and overlap with Start-seq and transcription termination signals using SQANTI3 (v5.2.1[4], --CAGE_peak start peaks, --polyA_peak TTS, --coverage STAR file, --skipORF).

Transcripts were filtered using stringent quality criteria, with high-confidence annotations requiring at least 90% transcript identity and matching exon structure. CDS models with >80% identity and >80% coverage of the query were assessed as matches. When the primary CDS failed to meet thresholds, alternative CDS predictions were evaluated. Overlapping CDS were simply annotated, while non-overlapping CDS constituted potential chimeras. These were split if separated by a termination signal; otherwise, they were flagged as potentially chimeric. For liftovers without reference matches, novel coding genes were created if their predicted CDS passed filtering criteria. For mono-exon transcripts, we required a TSS ratio >5, start location within a START-Seq peak, and polyA site within 10bp of the TTS. Novel non-coding transcripts required a TSS ratio >5 and polyA motif within 10bp of TTS, with mono-exons additionally requiring either start within a START-seq peak or end within a TTS site. Following liftover completion, transcripts were re-clustered into final gene models.

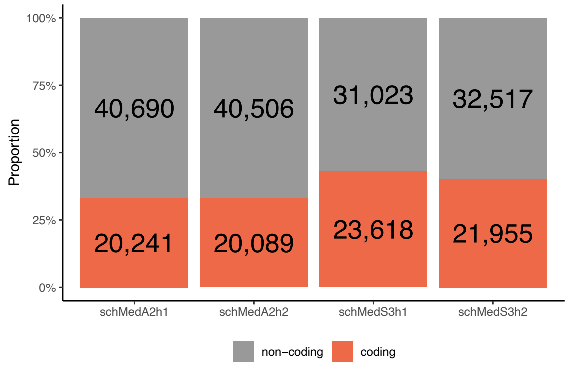

Supplementary Fig. 3 Summary of the number of genes that were inferred to be protein-coding or non-coding.

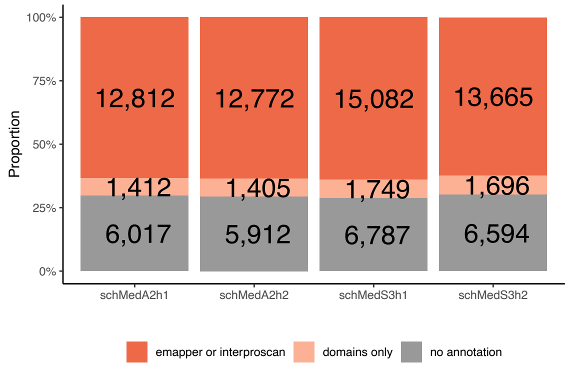

Supplementary Fig. 4 Summary of functional annotation of both the LabAsex and LabSex assemblies. Given are the number of genes with no functional annotation, with functional annotation based on InterProScan or emapper, and genes in which only functional domains were identified.

Supplementary Table 1 Summary of functional annotation of both the LabAsex and LabSex assemblies. Given are the number of genes with no functional annotation, with functional annotation based on InterProScan or emapper, and genes in which only functional domains were identified.

**Assembly**	**Annotation source**	**Genes**	**Proportion**
LabAsex haplotype 1	domains only	1412	0.07
LabAsex haplotype 1	emapper or interproscan	12812	0.63
LabAsex haplotype 1	no annotation	6017	0.30
LabAsex haplotype 2	domains only	1405	0.07
LabAsex haplotype 2	emapper or interproscan	12772	0.64
LabAsex haplotype 2	no annotation	5912	0.29
LabSex haplotype 1	domains only	1749	0.07
LabSex haplotype 1	emapper or interproscan	15082	0.64
LabSex haplotype 1	no annotation	6787	0.29
LabSex haplotype 2	domains only	1696	0.08
LabSex haplotype 2	emapper or interproscan	13665	0.62
LabSex haplotype 2	no annotation	6594	0.30

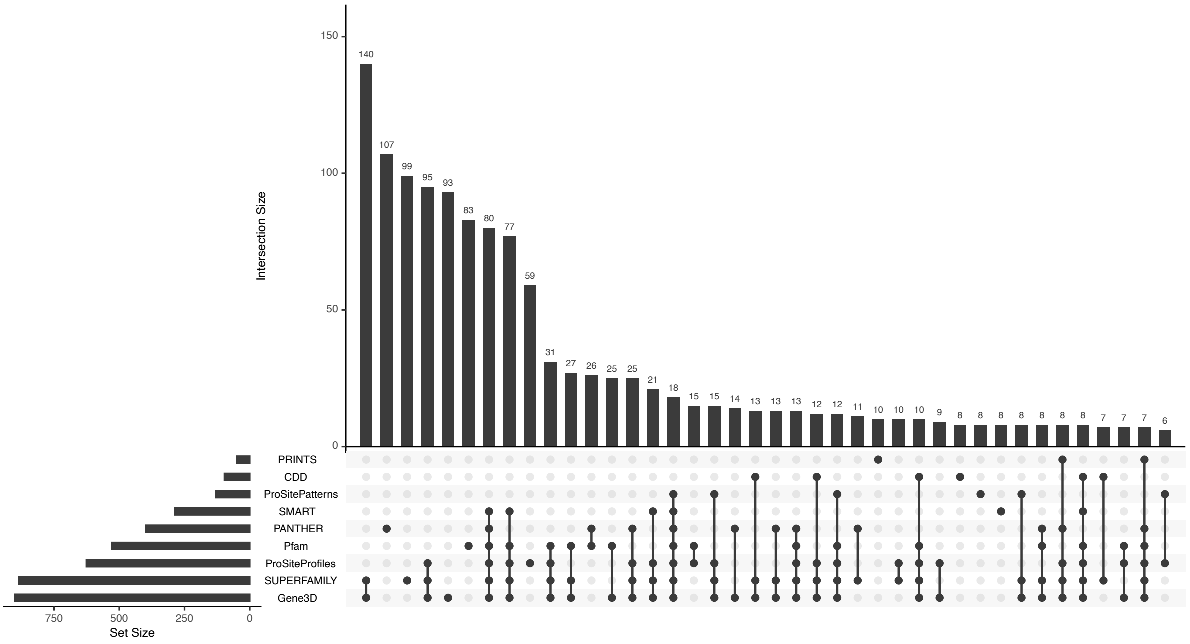

Supplementary Fig. 5 Intersection of gene annotation sets for the LabAsex haplotype 1 assembly. Upset plot showing the overlap of gene models across the different databases annotated using InterProScan. Horizontal bars represent the total number of genes identified by each method, while vertical bars indicate the size of each unique intersection set.

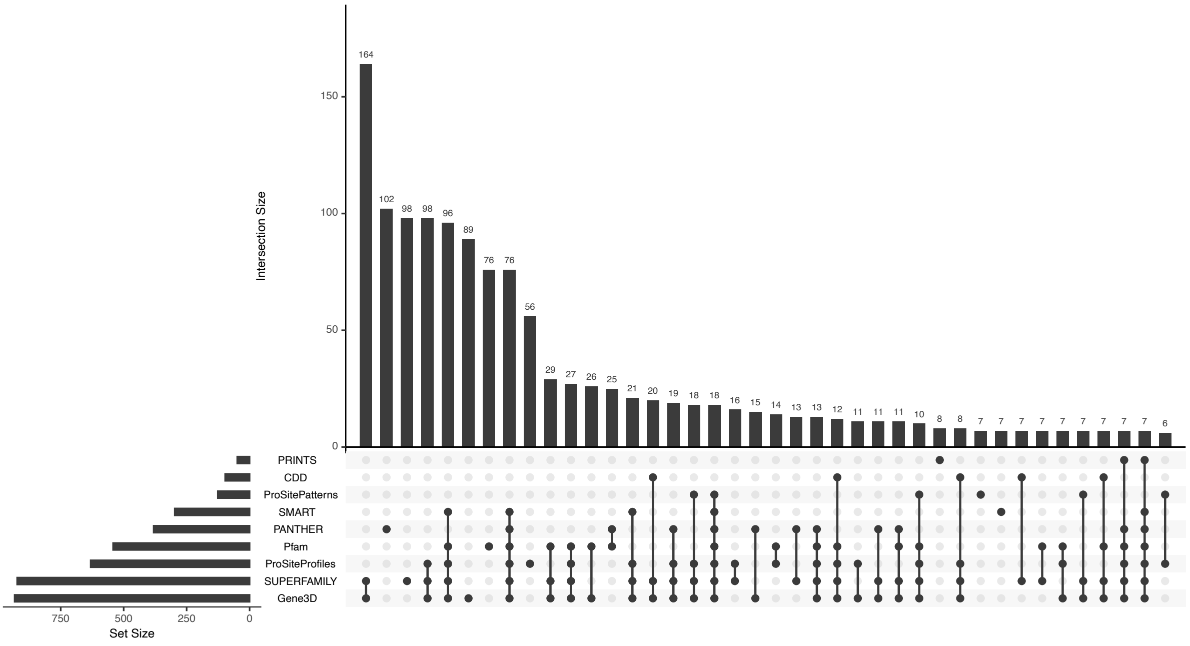

Supplementary Fig. 6 Intersection of gene annotation sets for the LabAsex haplotype 2 assembly. Upset plot showing the overlap of gene models across the different databases annotated using InterProScan. Horizontal bars represent the total number of genes identified by each method, while vertical bars indicate the size of each unique intersection set.

Supplementary Note 3 | Hi-C Analyses

Hi-C Quality Control

Supplementary Table 2 Mapping statistics of Hi-C data from the sexual LabSex strain (S2F18), the asexual lab strain (CIW4), three isolines of the Menorca population (MEN 9a, MEN 9f, MEN 5+6), and all Menorca isolines combined (MEN all). Mapping was performed with the LabSex (schMedS3) and LabAsex genome (schMedA2) as a reference.

**LabSex haplotype 1**	**LabSex**	**LabAsex**	**MEN all**	**MEN 9a**	**MEN 9f**	**MEN 5+6**
Input (M reads)	1138.4	584.7	482.6	153.5	144.7	184.4
Mean insert size (bp)	308	345	401	359	443	403
SE mapped (%)	92	89.2	79.2	74.4	79.8	82.6
SE truncated (%)	29.6	36.4	20.7	23.9	16.7	21
Mapped (M reads)	222.4	49	38	10.1	11.9	15.9
Duplicate (M reads)	33.5	9.8	8.5	2.2	2.8	3.5
Unique (M reads)	159.7	56.1	24.9	7.2	7	10.8
Cis >15kb (M reads)	89.7	32.3	14	3.9	4.1	6.1
Trans (M reads)	51.1	16.9	7.5	2.6	1.8	3.1
Restriction site (M reads)	42.2	3.3	7.4	2	2.3	3
Restriction site (%)	12.8	4.2	12.1	11.9	12.3	12.1
Cis/Trans	1.8	1.9	1.9	1.5	2.3	1.9

**LabAsex haplotype 1**	**LabSex**	**LabAsex**	**MEN all**	**MEN 9a**	**MEN 9f**	**MEN 5+6**
Input (M reads)	1138.4	584.7	482.6	153.5	144.7	184.4
Mean insert size (bp)	310	345	397	352	440	401
SE mapped (%)	88.4	92	83.5	78.7	84.3	86.7
SE truncated (%)	29.6	36.4	20.7	23.9	16.7	21
Mapped (M reads)	112.1	82.3	43.8	11.2	13.1	19.4
Duplicate (M reads)	15.1	17.2	10.1	2.5	3.1	4.5
Unique (M reads)	71.2	98.9	29.9	8.1	7.7	14.1
Cis >15kb (M reads)	37	57.9	17	4.3	4.5	8.1
Trans (M reads)	24.6	29.5	8.9	3	1.9	4.1
Restriction site (M reads)	24.6	4.6	8.3	2.2	2.6	3.5
Restriction site (%)	14.8	3.5	11.7	11.9	12.3	11.2
Cis/Trans	1.5	2	1.9	1.5	2.3	2

Hi-C of Chromosome 4

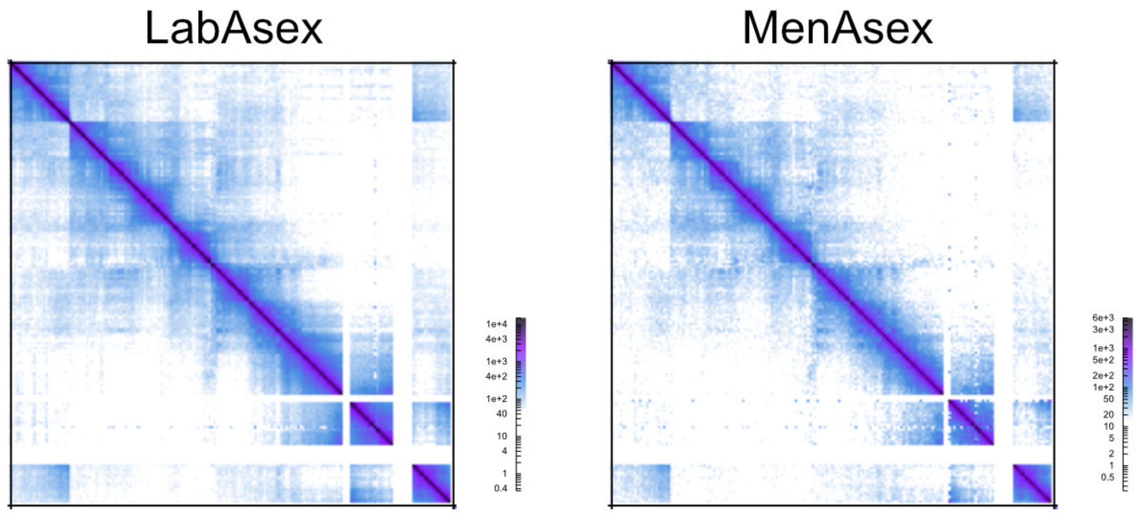

Supplementary Fig. 7 Hi-C contact map of Chr4 of the asexual reference genome (schMedA2h1), showing the Hi-C signal from the asexual laboratory strain (LabAsex, CIW4) and from a combination of three clonal lines generated from wild asexual individual from Menorca (MenAsex). Note the highly repetitive region towards the end of the scaffold, indicated by large stretches of no contact and the intense off-diagonal signal between the start and the end. Resolution of the contact map is 250 kb per pixel.

Hi-C Comparison of LabSex and LabAsex

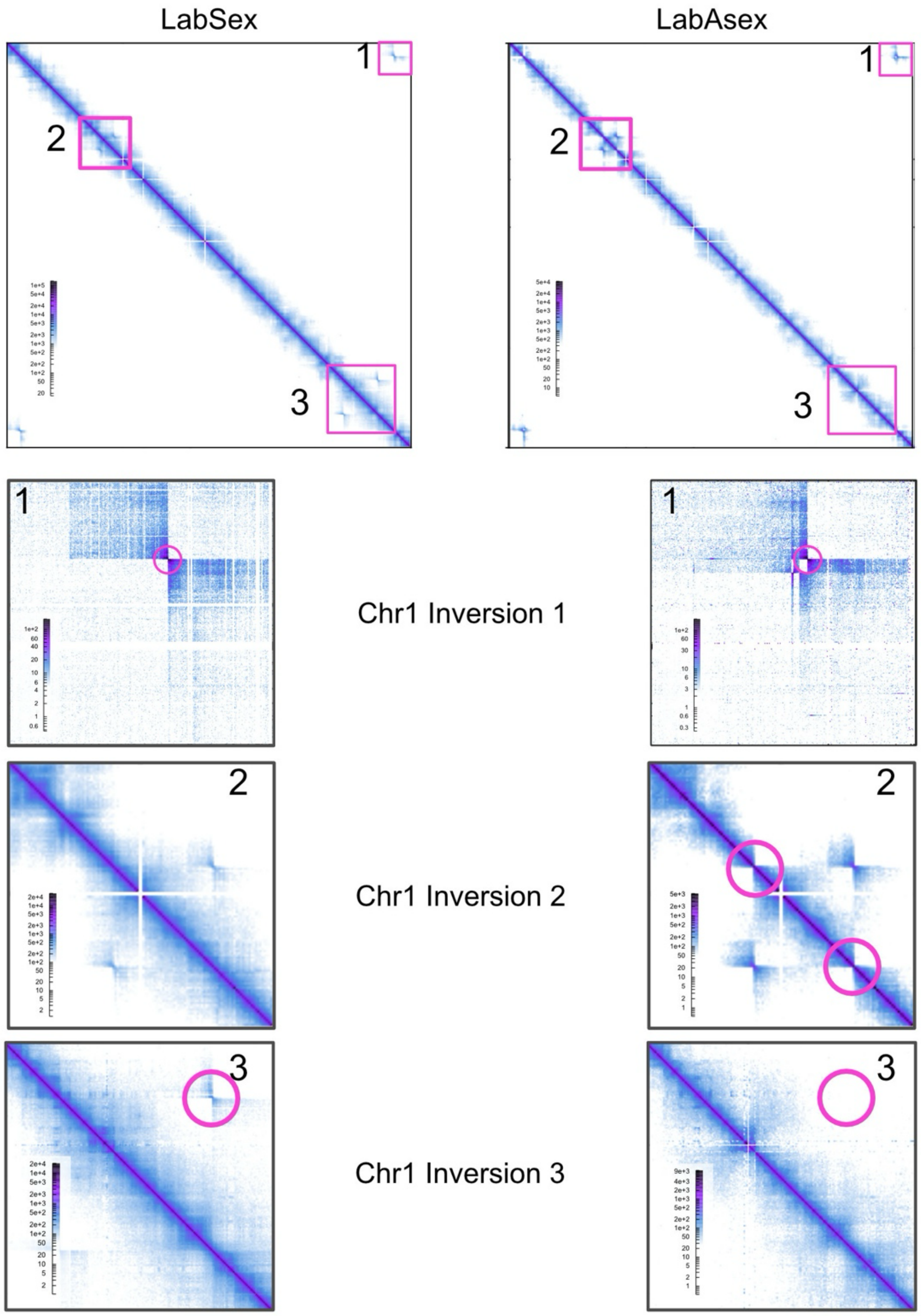

Supplementary Fig. 8 Hi-C signal of the sexual strain (LabSex, S2F18) and the asexual laboratory strain (LabAsex, CIW4) when mapped onto Chr1 of the LabSex reference genome (schMedS3h1). Pink boxes highlight regions of interest that are enlarged below. The enlarged regions of interest, showing Hi-C signal in LabSex (left) and LabAsex (right). The signal indicates that the asexual strain shares the large inversion (1) but does not have the two smaller inversions (2,3). Note that asexual haplotype 1 is inverted in relation to the sexual reference chromosome leading to the off-diagonal signal but a lack of signal on the diagonal in (2). Further note that the signal in 1 is duplicated in LabAsex due to a duplication of that region in the asexual strain.

Hi-C contact matrices generated with the Arima pipeline were used as described in the main text. Hi-C interaction patterns were compared among LabSex, LabAsex, and MenAsex samples at 1 Mb and 25 kb resolutions. Analyses were performed using HiCExplorer (v3.7.6[5]). Prior to matrix correction, matrices were normalized using hicNormalize with the smallest method to account for differences in sequencing depth. Normalized matrices were then corrected using Knight–Ruiz (KR) balancing with hicCorrectMatrix, and interaction signals were compared across samples using hicCompareMatrices.

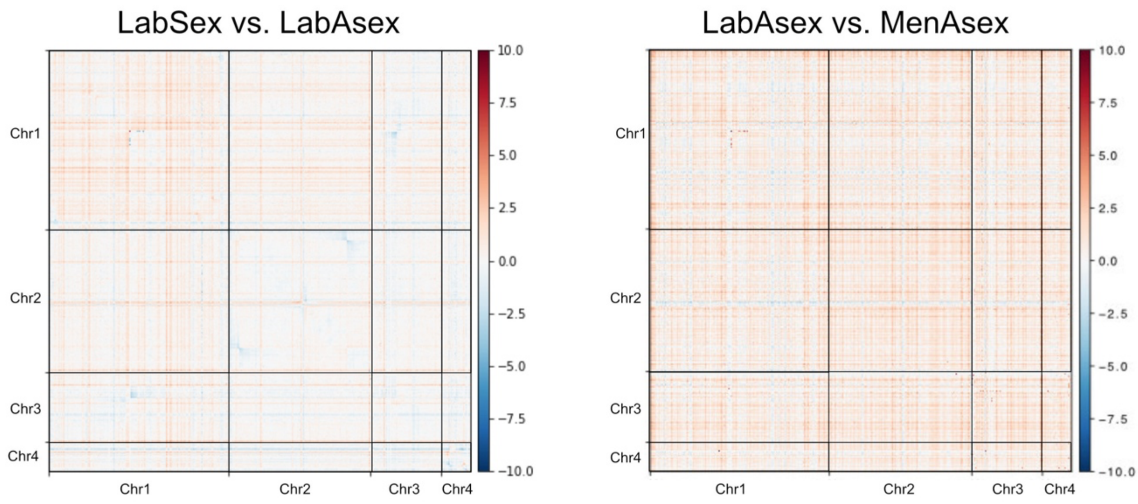

Supplementary Fig. 9 Log 2-fold difference in Hi-C signal between LabSex and LabAsex and between LabAsex and MenAsex. LabSex lacks the off-diagonal signals indicating the Chr1/3 translocation and the Chr2 inversion. No clear difference is found between LabAsex and MenAsex at 1 Mb resolution.

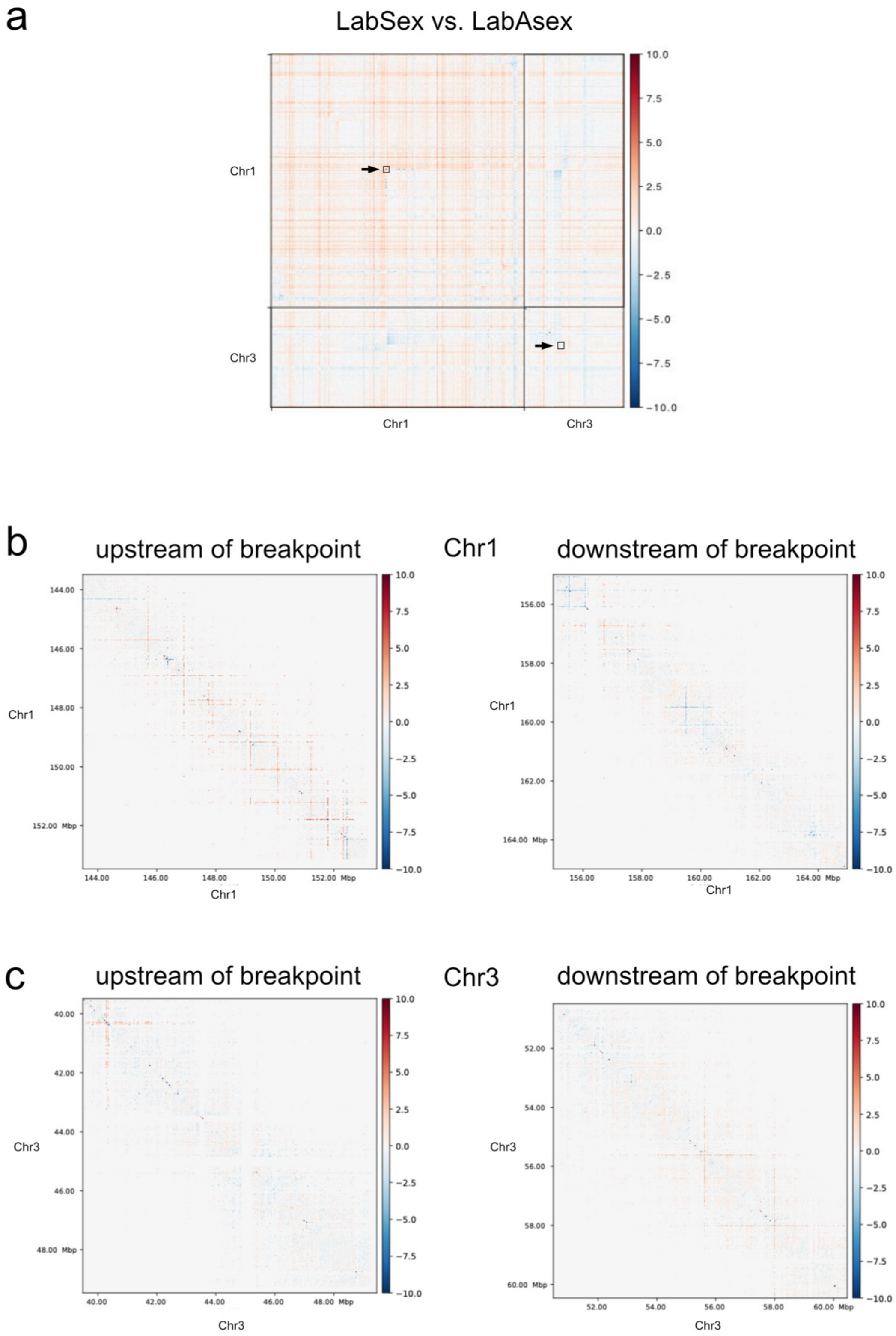

Supplementary Fig. 10 Log 2-fold difference in Hi-C signal between LabSex and LabAsex in proximity to the homologous Chr1/3 breakpoint region. No pattern of differential chromatin organization is apparent on Chr1 or Chr3. Data is shown on Chr1 and Chr3 of the LabSex reference genome at 1 Mb resolution and at 25 kb resolution for b-c.

Hi-C Menorca Isolines

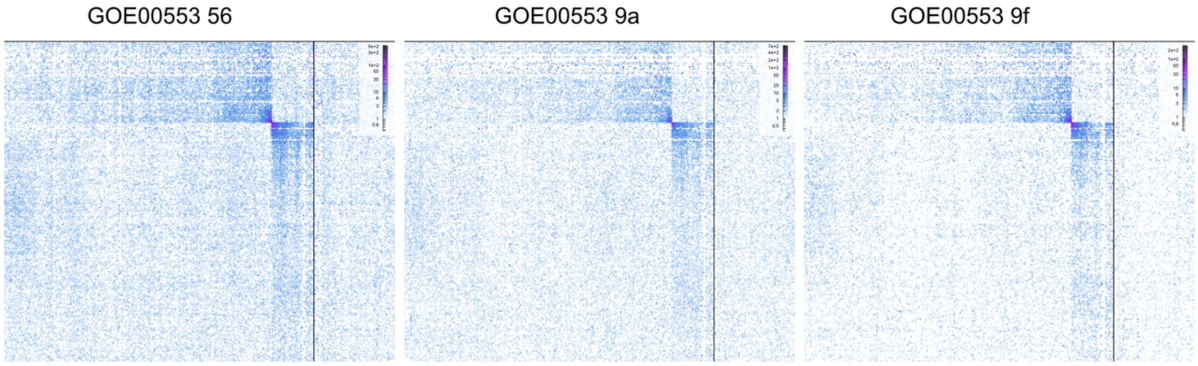

Supplementary Fig. 11 Hi-C signal of three clonal lines derived from wild asexuals from Menorca. Shown is the signal for the Chr1 inversion 1. Resolution of the contact map is 250 kb per pixel.

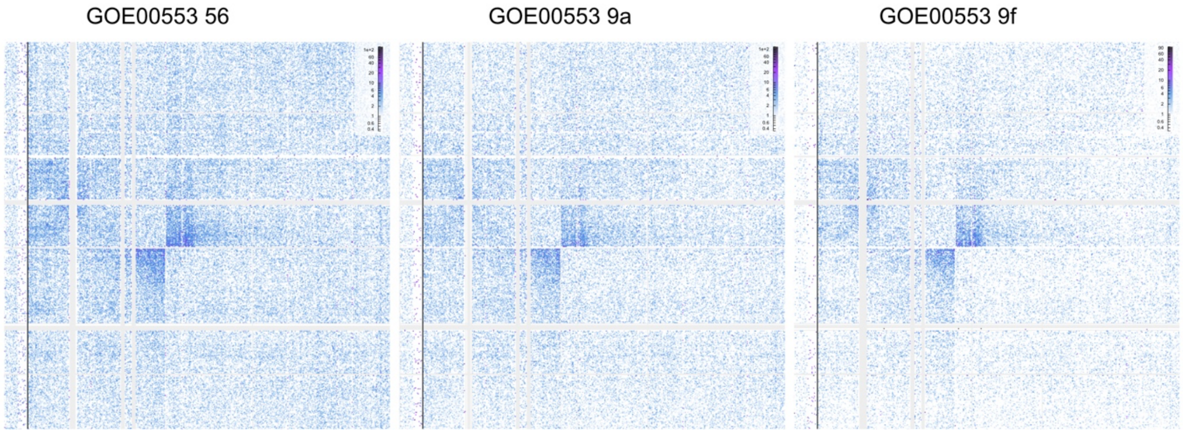

Supplementary Fig. 12 Hi-C signal of three clonal lines derived from wild asexuals from Menorca. Shown is the signal for the Chr1/3 translocation. Resolution of the contact map is 250 kb per pixel.

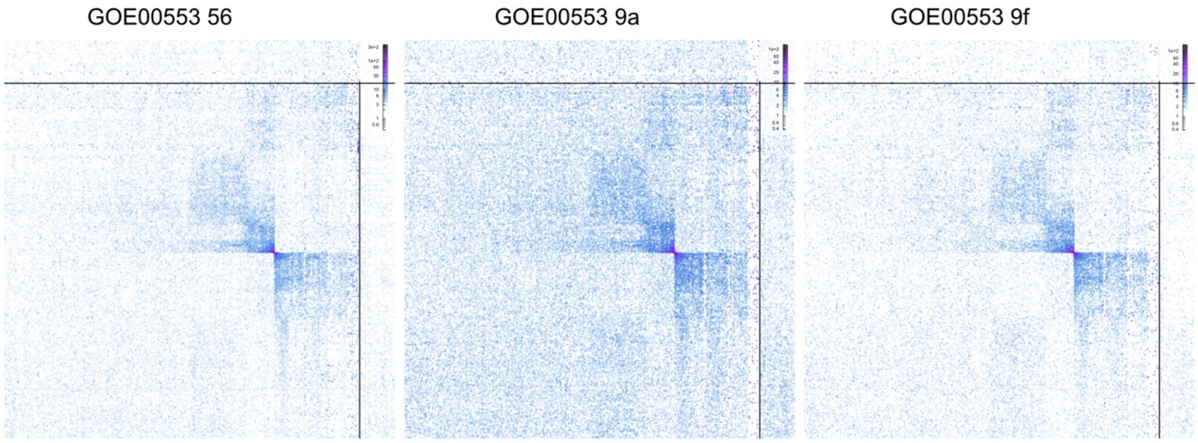

Supplementary Fig. 13 Hi-C signal of three clonal lines derived from wild asexuals from Menorca. Shown is the signal for the Chr2 inversion. Resolution of the contact map is 250 kb per pixel.

Supplementary Note 4 | Karyology from the Literature

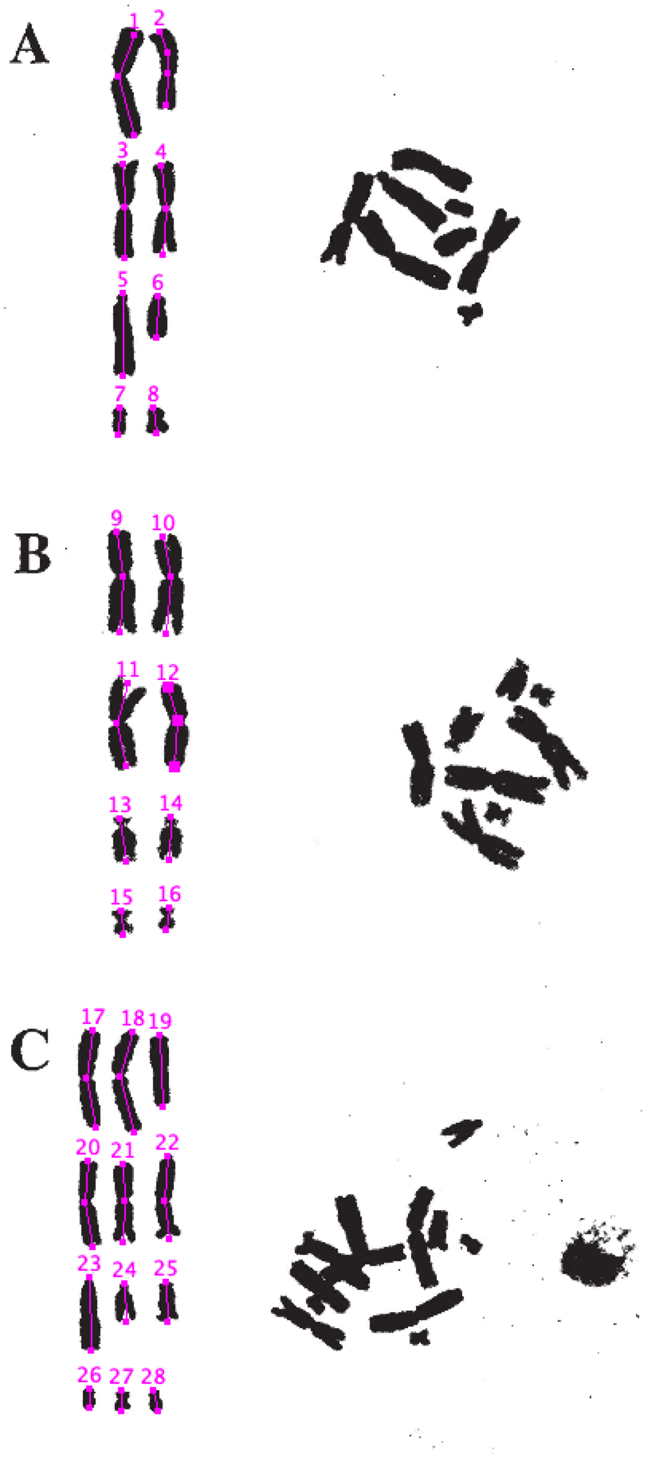

Fig 1 - Mitotic metaphase plates and karyotypes of *Schmidtea mediterranea*. A) 2n = 8, asexual diploid; B) 2n = 8, sexual diploid; C) 3n = 12, asexual triploid.

Supplementary Fig. 14 Estimation of relative chromosome size based on the publication of Baguñà et al. 1999.

Supplementary Table 3 Literature-based relative size estimates of *S. mediterranea* chromosomes. Values from Baguñà et al. 1999 were extracted as shown in Supplementary Fig. 14.

**Reference**	**Population**	**Chromosome**	**Relative length**
Baguñà et al. 1999	asexual 2n	Chr1	197.7
		Chr1'	140.7
		Chr2	176
		Chr2'	168.8
		Chr3'	156
		Chr3	78.1
		Chr4	52
		Chr4'	50.4
Baguñà et al. 1999	sexual 2n	Chr1	190.8
		Chr1'	186.1
		Chr2	160.2
		Chr2'	152.2
		Chr3	82.6
		Chr3'	82
		Chr4	46
		Chr4'	44.4
Baguñà et al. 1999	Menorca 3n	Chr1	185.9
		Chr1'	194.6
		Chr1''	134.8
		Chr2	163.5
		Chr2'	148.7
		Chr2''	155.5
		Chr3	136
		Chr3'	74.1
		Chr3''	74.1
		Chr4	38
		Chr4'	38.5
		Chr4''	40.1
Ribas 1990	Barcelona 2n	Chr1	41.95
		Chr1'	29.38
		Chr2	33.85
		Chr2'	33.85
		Chr3	27.86
		Chr3'	15.41
		Chr4	8.82
		Chr4'	8.82

Reference	Population	Chromosome	Relative length
Ribas 1990	Menorca 3n	Chr1	42.19
		Chr1'	30.49
		Chr2	33.37
		Chr2'	33.37
		Chr3	27.78
		Chr3'	17.78
		Chr4	8.71
		Chr4'	8.71
De Vries 1984	Mallorca 2n	Chr1	37.74
		Chr2	31.77
		Chr3	29.5
		Chr3'	16.18
		Chr4	7.6
	Barcelona 2n	Chr1	34.96
		Chr2	31.76
		Chr3	28.31
		Chr3'	18.08
		Chr4	10.55

Supplementary Table 4 Size of chromosome scaffolds in the LabAsex assembly, the scaffold names, and the assigned chromosome to allow comparisons to data in Supplementary Table 3.

Population	Chr	Scaffold	Scaffold length (bp)
Barcelona CIW4	Chr1	Chr1_h1	327,432,606
Barcelona CIW4	Chr1'	Chr3_h2	224,520,763
Barcelona CIW4	Chr2	Chr2_h1	272,298,851
Barcelona CIW4	Chr2'	Chr2_h2	267,951,672
Barcelona CIW4	Chr3	Chr3_h1	119,429,788
Barcelona CIW4	Chr3'	Chr1_h2	219,263,528
Barcelona CIW4	Chr4	Chr4_h1	46,130,935
Barcelona CIW4	Chr4'	Chr4_h2	46,645,998

Supplementary Table 5 Relative differences (Δ) between Chr1 and Chr3 homologs in the asexual strain, based on estimates from the literature and the genome assembly presented in this study. Our estimate agrees well with the literature.

Population	Publication	Δ Chr1/Chr1'	Δ Chr3/Chr3' (%)
Barcelona 2n	Ribas 1990	30	44.7
Barcelona 2n	Baguñà et al. 1999	28.8	49.9
Barcelona 2n	De Vries 1984	-	36.1
Barcelona 2n	This study	31.4	45.5
Mallorca 2n	De Vries 1984	-	45.2
Menorca 3n	Ribas 1990	27.7	36
Menorca 3n	Baguñà et al. 1999	30.7	45.5

Supplementary Note 5 | Chromosome FISH

Generation of DNA probes specific to the predicted centromeric satellite DNA repeats

Total gDNA was isolated from a pool of 10 worms. Before DNA extraction, worms were washed in 0.05% NAC solution (N-acetyl-L-cysteine) for 1 min. Worms were disrupted by homogenization, and DNA was extracted with the phenol-chloroform procedure followed by ethanol precipitation. The extracted gDNA was used as a template in the following PCR.

Amplification of fragments of centromeric DNA repeats was performed using standard PCR with specific forward and reverse primers (Supplementary Data 1). Then PCR products were labeled in an additional 20 cycles of PCR using corresponding specific primers in the presence of Flu-12-dUTP, fluorescein-5(6)-carboxamidocaproyl-[5(3-aminoallyl)2'-deoxyuridine-5'-Triphosphate)] (Biosan, Novosibirsk, Russia), or TAMRA-5-dUTP, 5-tetramethylrhodamine-dUTP (Biosan, Novosibirsk, Russia). Modified oligonucleotides (CEN-SmedSat1_158 and CEN-SmedSat2_159) were ordered in the laboratory of synthetic biology (ICBFM SB RAS, Novosibirsk, Russia). All primers and oligo DNA probes used in this study are listed in Supplementary Table 2.

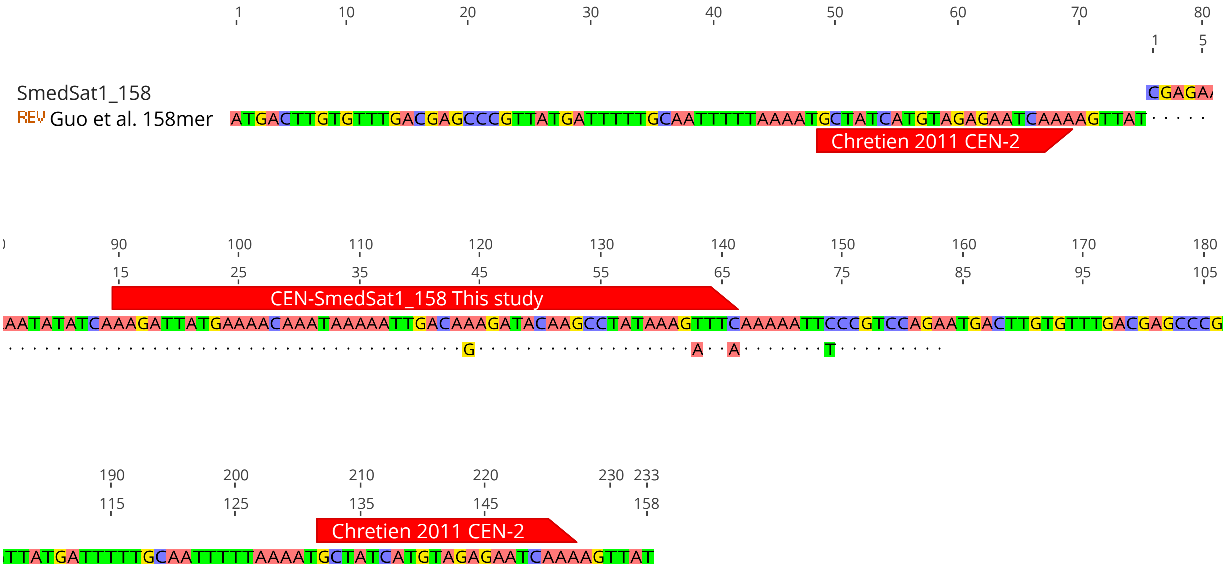

Supplementary Fig. 15 Similarity of the SmedSat 158mer repeat targeted in this study to the k-mer studied by Guo et al., showing the predicted probe binding location of the probe used in this study and the one used by Chretien 2011.

Chromosome Slide Preparation

Metaphase spreads were obtained from dividing cells from regenerated tissue. Worms of *S. mediterranea* from both sexual and asexual strains were cut and left to regenerate in 6-well plates (1 individual per well) containing 1XPW for two days. Then the regenerated tissue of the worm was isolated and used for the following slide preparation. For that, the pieces of tissue were placed into 0.2% colchicine (in 1XPW) for 1 h at RT. Immediately after, they were transferred into 0.2% KCl (in 1XPW) for 1 h at RT. After that, the treated material was carefully placed onto the clean, dry slides, and residues of the solution were removed. Immediately after, a small amount (about 20 µL) of the fixative solution I (glacial acetic acid:ethanol:bidist H2O, 3:3:4) was added, and the material was macerated in the fixative with a glass needle. Then the slide with the macerated material was placed into water bath vapors, and the fixatives II and III (glacial acetic acid:ethanol, 1:1, and glacial acetic acid, correspondingly) were placed onto the material and left for 2-3 min. Then the prepared chromosome slides were air-dried at RT and used for the following procedures.

Fluorescent *in situ* Hybridization

FISH experiments with DNA probes generated using PCR were carried out as was described earlier (Zadesenets et al., 2020). Briefly, after a pretreatment step including RNase, pepsin, and fixation, the DNA of chromosomal material and generated DNA probes was denatured simultaneously at 72°C for 2 min. For hybridization, chromosome slides were incubated overnight at 37°C. Afterward, slides were washed using 50% formamide in 2×SSC (three times for 5 min), 2×SSC (two times for 5 min), and 0.2×SSC (two times for 5 min). All washings were done at 45°C.

Oligo-FISH was performed on metaphase chromosomes of *S. mediterranea* (sexual and asexual strains). Pre-treatment of chromosome slides was done according to the standard FISH technique (Zadesenets et al., 2020). Oligo-FISH probes (CEN-SmedSat1_158, 22 μM, and CEN-SmedSat2_159, 67 μM) were dissolved in hybridization mixture (50% formamide, 10% dextran sulfate, 4×SSC) until the final concentration (1:500 and 1:1000, correspondingly). After that, the simultaneous hybridization of DNA of chromosome slides and oligo-FISH probes was done at 72°C for 2 min and followed by hybridization for two days at 37°C in a dark place. Then the slides were washed with 2× SSC, 0.5× SSC, and 1× TNT (100 mM Tris-HCl, 150 mM NaCl, 0.1% Tween 20, pH 7.4) for 15 min each at RT. After chromosome slides were dehydrated in 70%, 90%, and 100% graded ethanol for 2 min each at RT and air-dried. Metaphase chromosomes were counterstained with 4',6-diamidino-2-phenylindole solution (DAPI) (VectaShield, USA) according to standard protocol.

Quantification of FISH Signal in LabAsex and LabSex

Supplementary Table 6 Signal quantification of oligo-FISH signal of the 158mer and 159mer based on 50 metaphase plates from different specimens of the asexual laboratory strain. When it was cytologically possible to separate the homologs, they were labeled accordingly. Otherwise, they were grouped by signal intensity.

Oligo	Chr1		Chr2		Chr3		Chr4	
	Chr1	Chr1'	hom. 1	hom. 2	Chr3	Chr3'	hom. 1	hom. 2
158mer	2.84±0.618	2.43±0.5	2	2	1.02±0.143	1.65±0.48	0	0
159mer	0	0	0	0	1	1.65±0.52	2	1

Supplementary Table 7 Signal quantification of oligo-FISH signal of the 158mer and 159mer based on metaphase plates from different specimens of the sexual laboratory strain. Homologous chromosomes were grouped by signal intensity.

Oligo	Chr1		Chr2		Chr3		Chr4	
	hom. 1	hom. 2	hom. 1	hom. 2	hom. 1	hom. 2	hom. 1	hom. 2
158mer	2.60±0.599	2.53±0.50	2.23±0.42	2.17±0.38	1.75±0.43	1.55±0.5	0	0
159mer	0	0	0	0	1	1	2	2

FISH Images

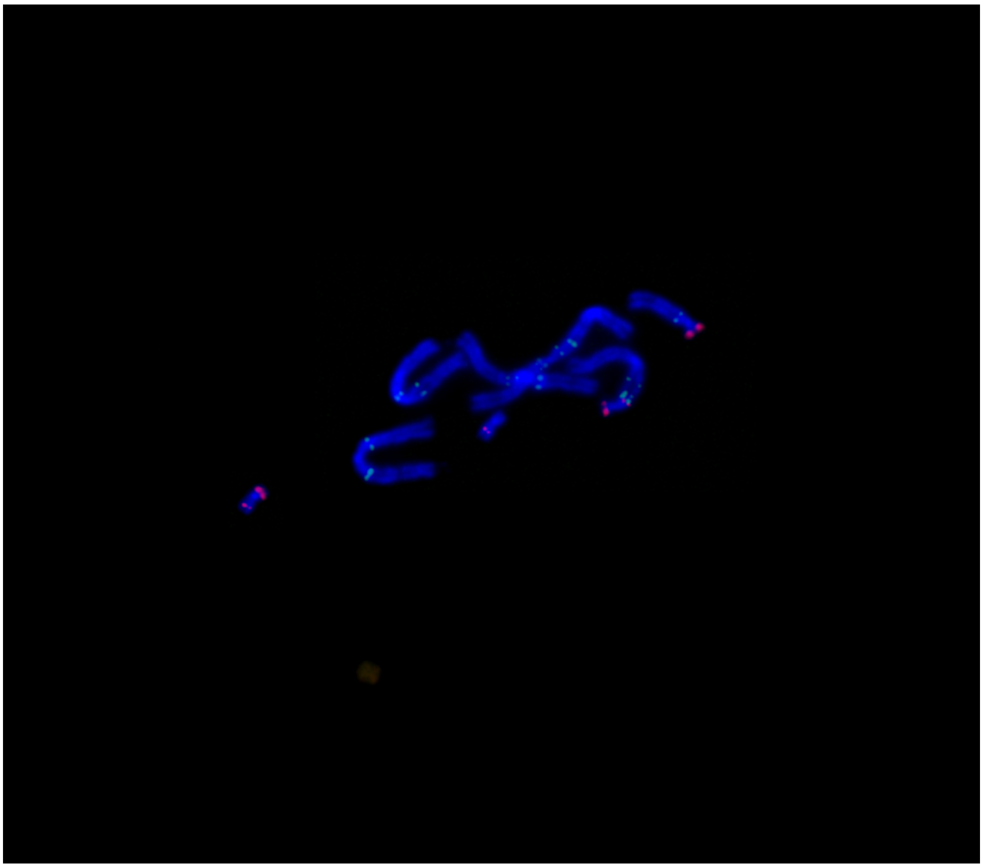

Supplementary Fig. 16 Raw metaphase spread used for visualization in Supplementary Fig. 1d of the main text. Note that overlapping chromosomes had to be digitally separated.

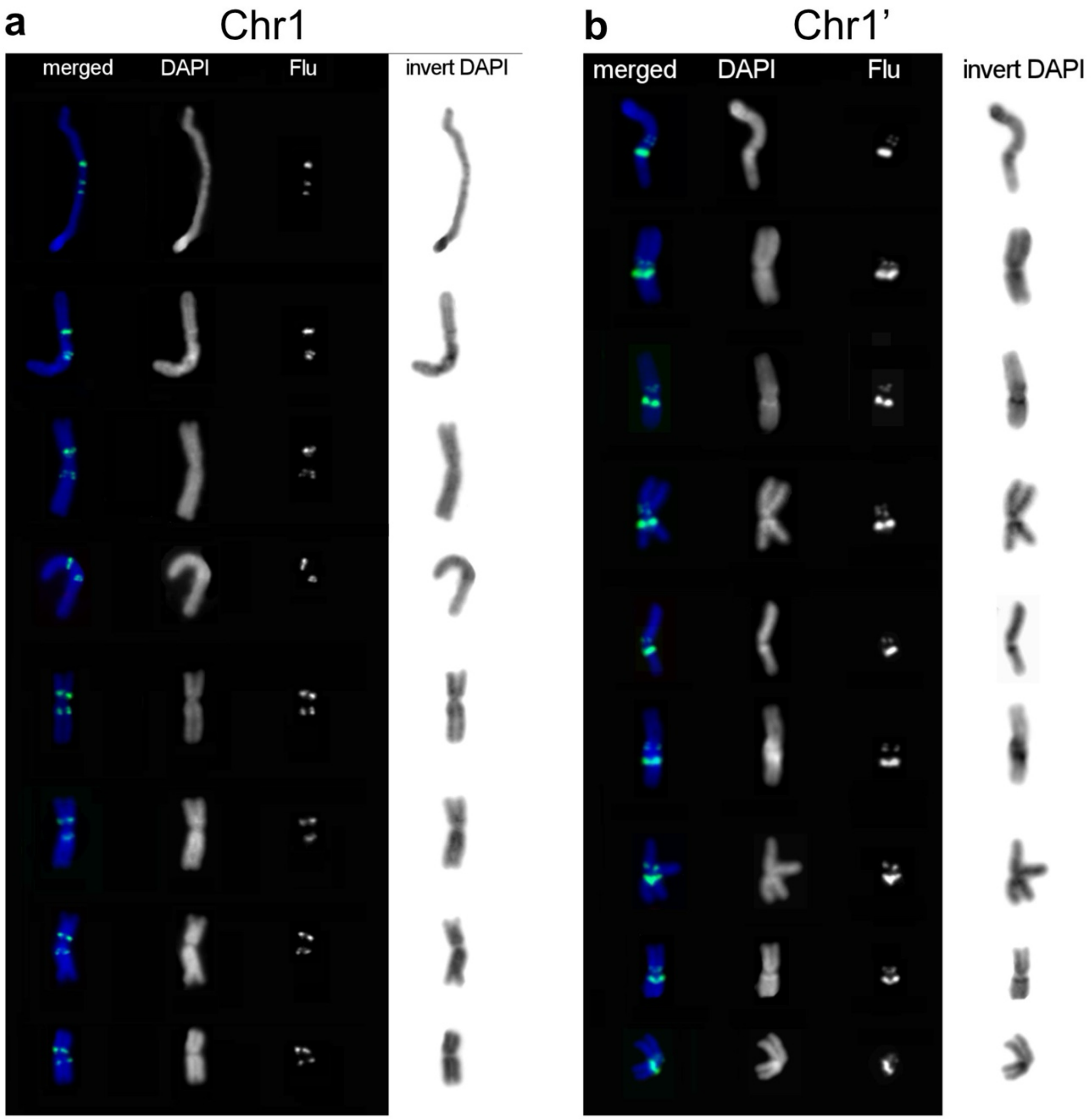

Supplementary Fig. 17 Example variation in chromosome FISH of Chr1 (a) and the rearranged Chr1’ (b) in the asexual laboratory strain. Flu signal shows labelling of the 158mer.

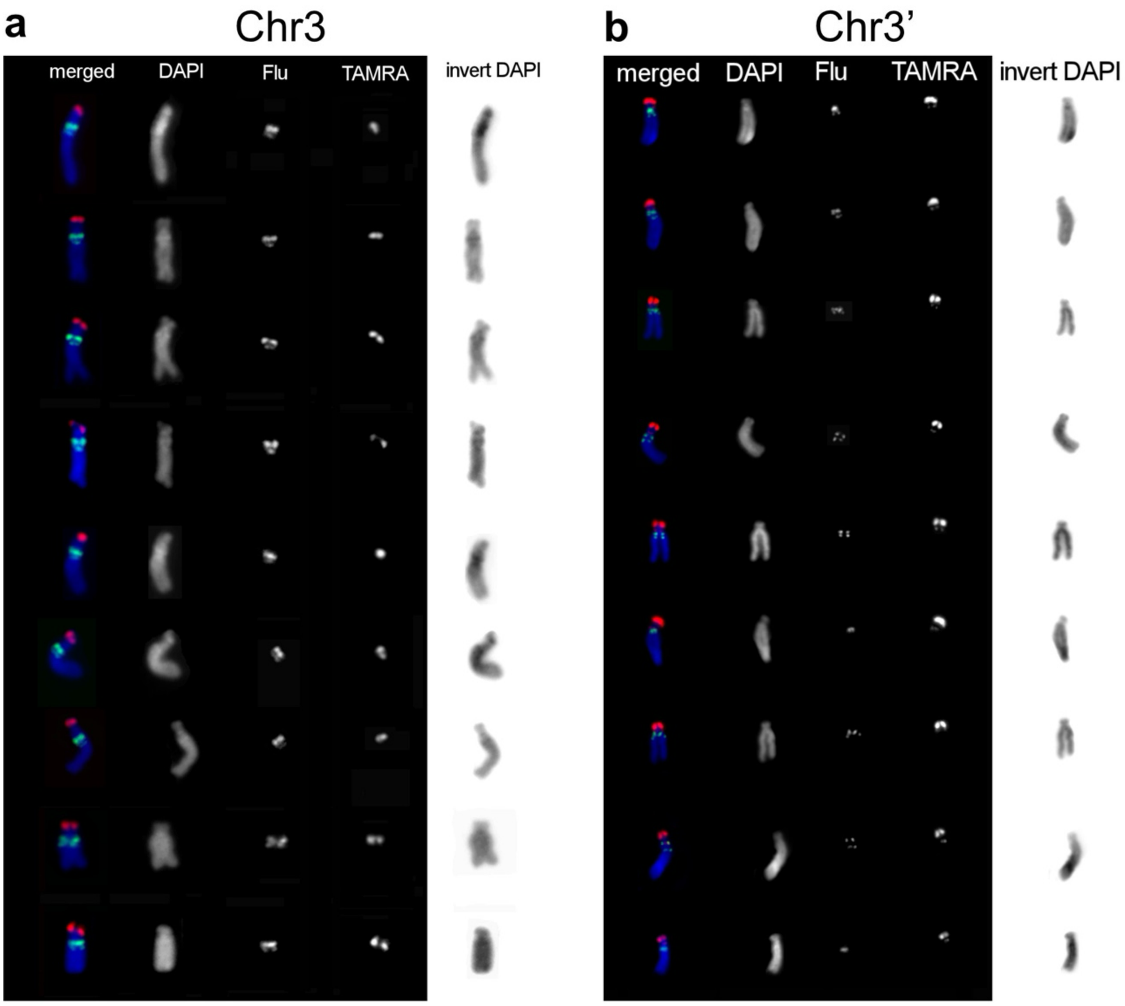

Supplementary Fig. 18 Example variation in chromosome FISH of Chr3 (a) and the rearranged Chr3’ (b) in the asexual laboratory strain. Flu signal shows labelling of the 158mer and TAMRA shows labelling of 159mer.

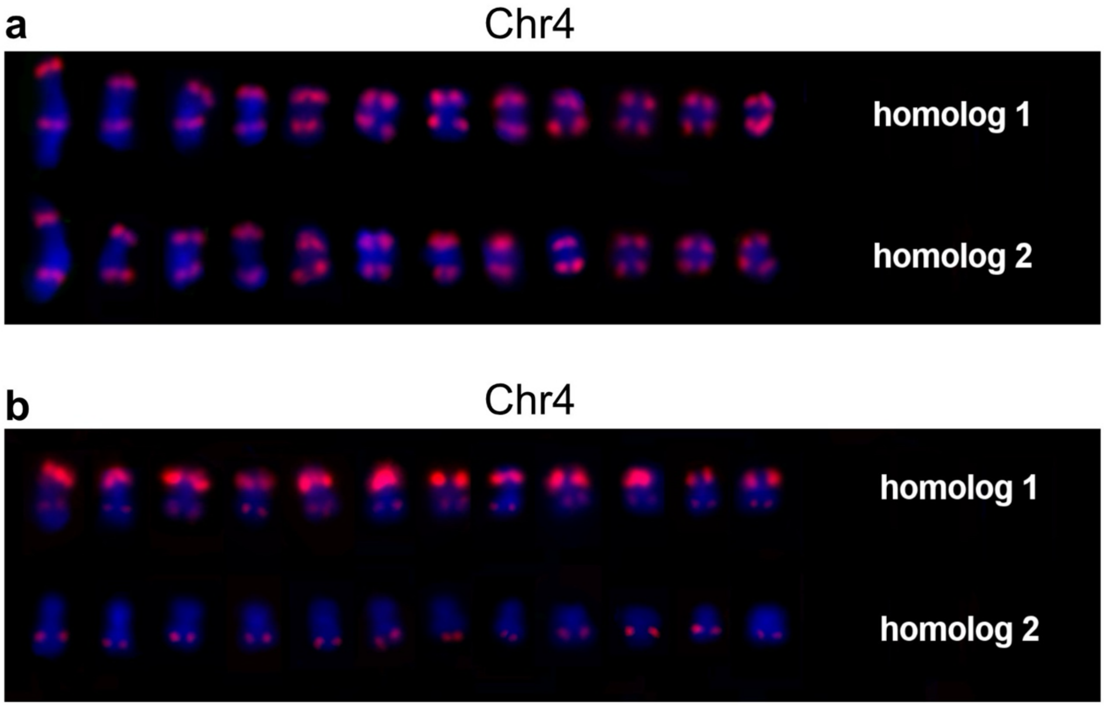

Supplementary Fig. 19 Example variation in chromosome FISH of Chr4 and Chr4' in the sexual laboratory strain (a) and Chr1 and asexual laboratory strain (b). Signal shows labelling of the 159mer.

Supplementary Note 6 | Synteny between LabSex and LabAsex

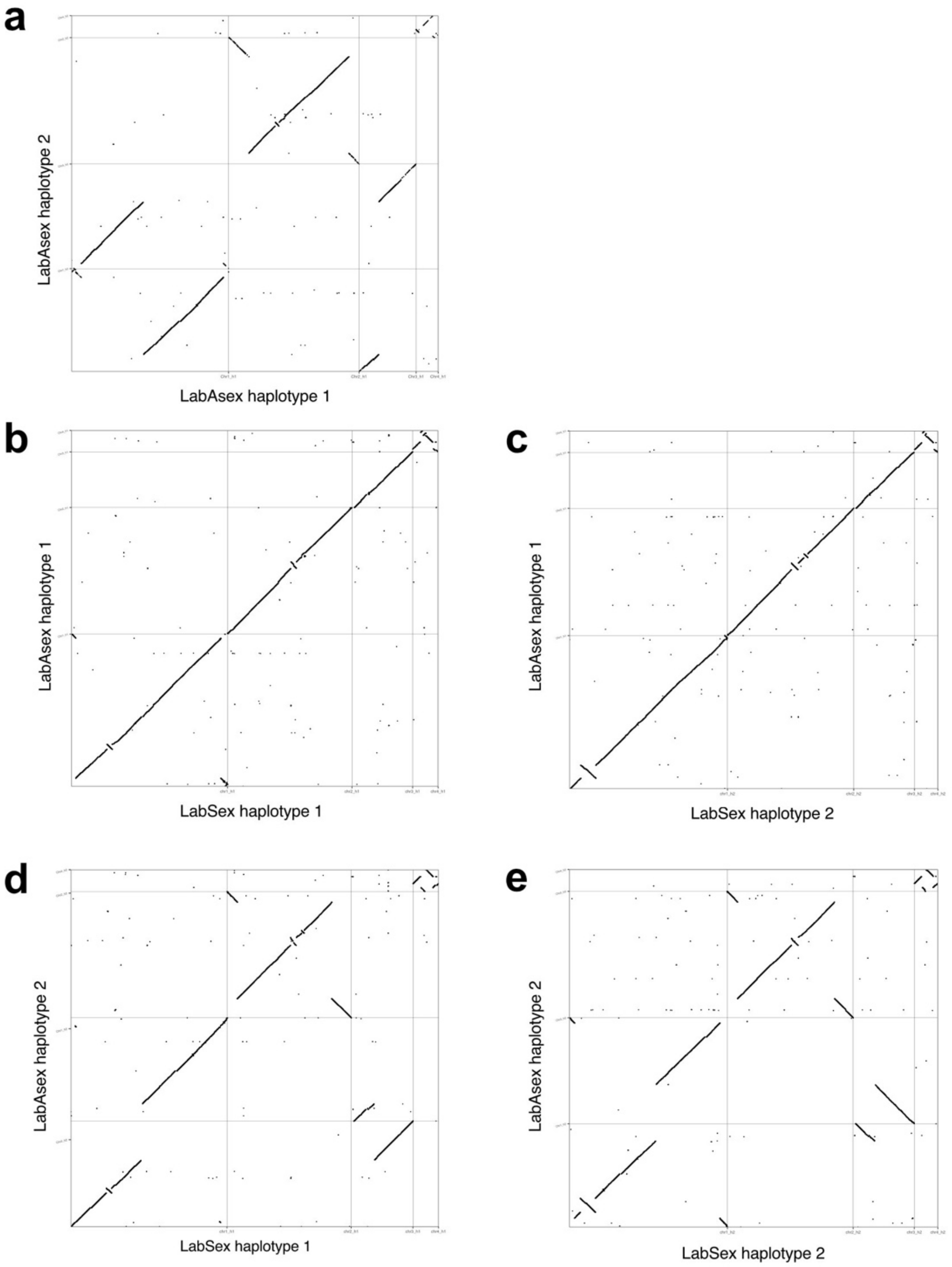

Supplementary Fig. 20 Dotplots representing whole genome alignments between the sexual (LabSex) and asexual (LabAsex) genome assemblies (a) LabAsex haplotype 1 and LabAsex haplotype 2; (b) LabSex haplotype 1 and LabAsex haplotype 1; (c) LabSex haplotype 2 and LabAsex haplotype 1; (d) LabSex haplotype 1 and LabAsex haplotype 2; (e) LabSex haplotype 2 and LabAsex haplotype 2. Dots represent minimap2 alignments with a minimum length of 1 kb and a mapping quality >30.

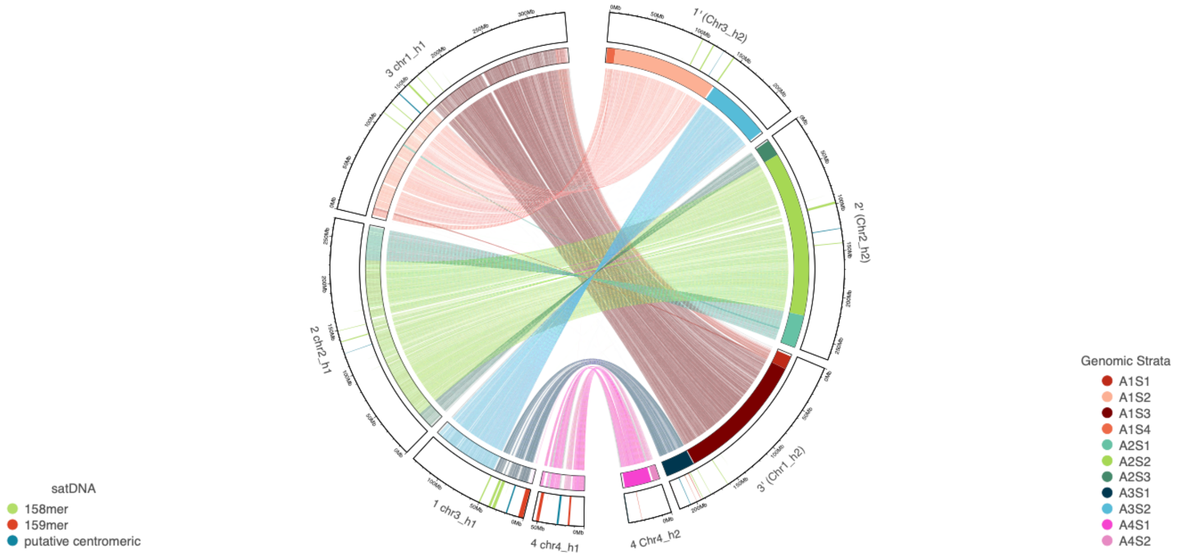

Supplementary Fig. 21 Whole-genome alignment-based liftover of LabAsex haplotype 2 genomic strata, which represents the derived haplotype with the reciprocal translocation, shown on the right, onto LabSex haplotype 1 shown on the left.

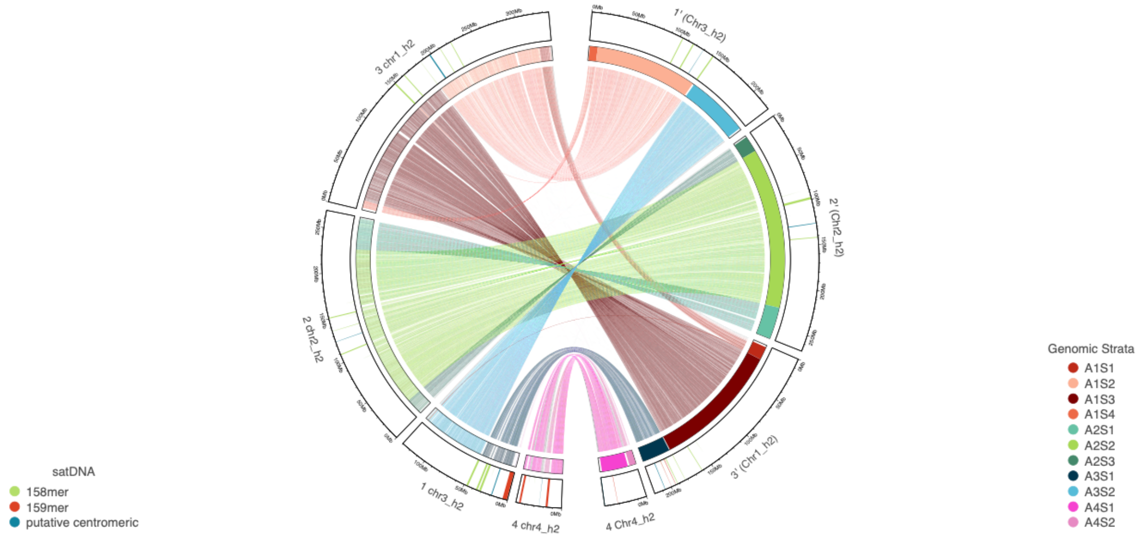

Supplementary Fig. 22 Whole-genome alignment-based liftover of LabAsex haplotype 2 genomic strata, which represents the derived haplotype with the reciprocal translocation, shown on the right, onto LabSex haplotype 2 shown on the left.

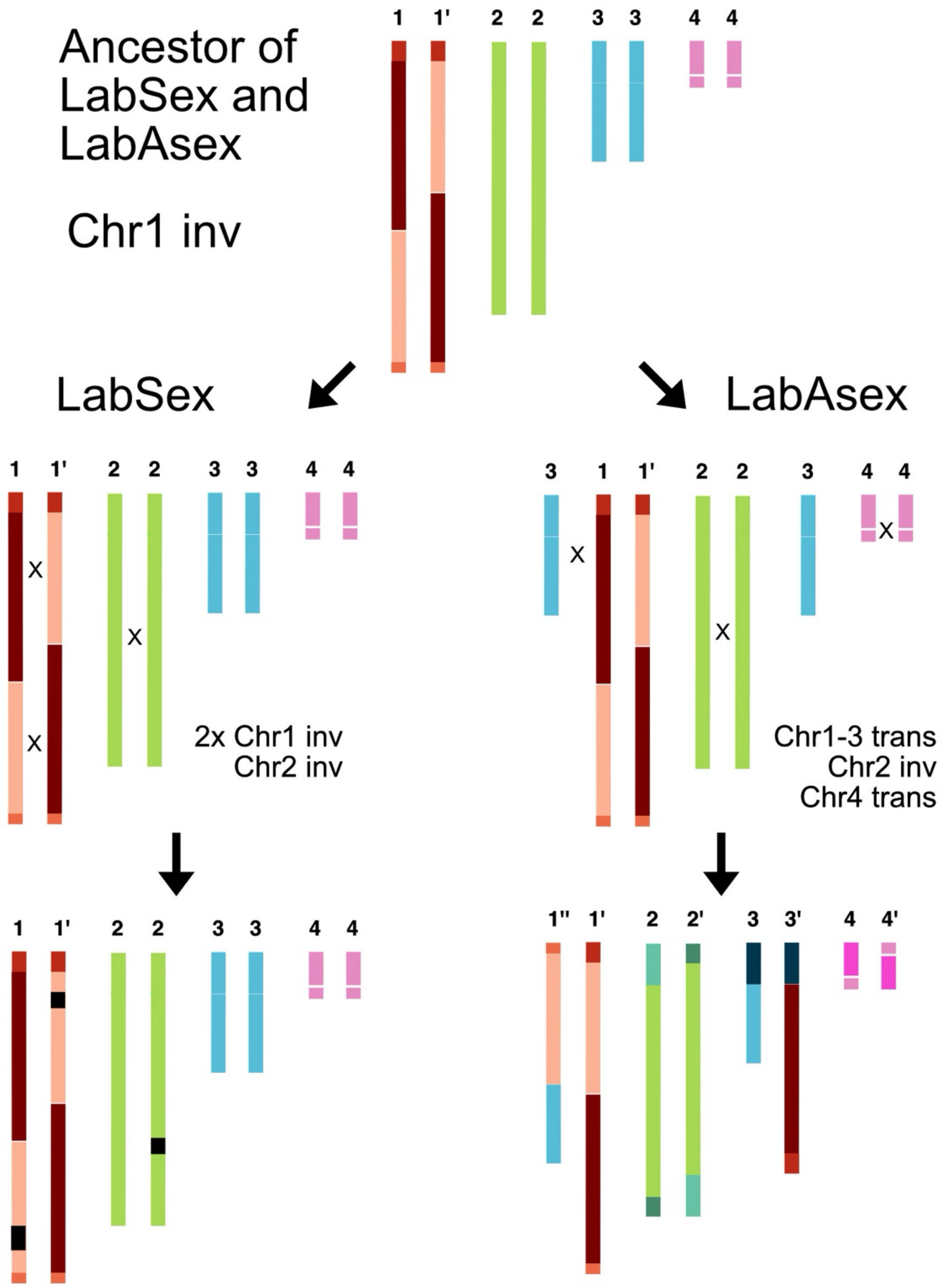

Supplementary Fig. 23 Most parsimonious history of chromosomal rearrangements explaining the observed structural differences between the sexual LabSex and asexual LabAsex strains. The Chr1 inversion is shared, while all other rearrangements have occurred independently.

Supplementary Note 7 | Reproduction-related Genes

To gain functional information about gene loss candidate genes, we established a detailed annotation of reproduction-related genes. We generated gene expression profiles from sexual and asexual wild-type animals, as well as from sexual control *RNAi* (eGFP) and *ophis* RNAi, which is known to block the development of all reproductive system components at an early stage[6,7]. Using these data, we annotated 2,136 protein-coding genes as high-confidence reproduction-related if they were significantly down-regulated in *ophis* RNAi and asexual wild-type compared to sexual control (see Methods, Main text Fig. 3d-f, Supplementary Table 4-6). In this section, we will detail the experimental design and the results of this analysis.

Power Calculation

To determine the appropriate sample size and read depth we performed a power calculation using ssizeRNA single function from the R package ssizeRNA. We oriented our projected number of differentially expressed genes based on the comparison of sexual and asexual wild-type data from Davies et al., [8] where, with an average read count of 20 million per library, 52% of transcripts were significantly differentially expressed and 60% had a log2fold-change of >2. This determined that increasing the coverage to 40 million reads per library and a sample size of six would result in an acceptable beta of 0.72, assuming an alpha of 0.05.

Confirmation of RNAi via Phenotyping

We confirmed the phenotype of sexual animals by observing a gonopore and laid cocoons in the eGFP RNAi and sexual wild-type group. While in the ophis RNAi treatment only two animals had a slightly light spot in the area where a gonopore might appear. Size was assessed six days before collection and revealed no significant size difference between RNAi groups, but slightly larger size in wild-type, likely because they received *ad-libitum* food (Supplementary Fig. 24). We also confirmed the loss of major reproductive structures in *ophis* RNAi via whole-mount DAPI staining of animals in the treatment groups (Supplementary Fig. 25).

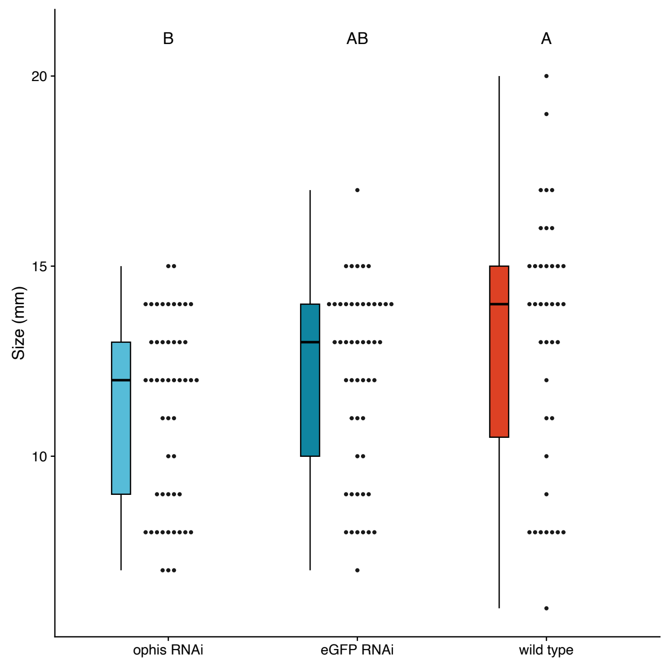

Supplementary Fig. 24 Body length of the three sexual treatment groups six days before collection. Boxes indicate the interquartile range (IQR), with the median as a horizontal line. Whiskers extend to 1.5×IQR, and individual points represent raw data. Different letters indicate statistically significant differences between groups (Tukey HSD, $p < 0.05$).

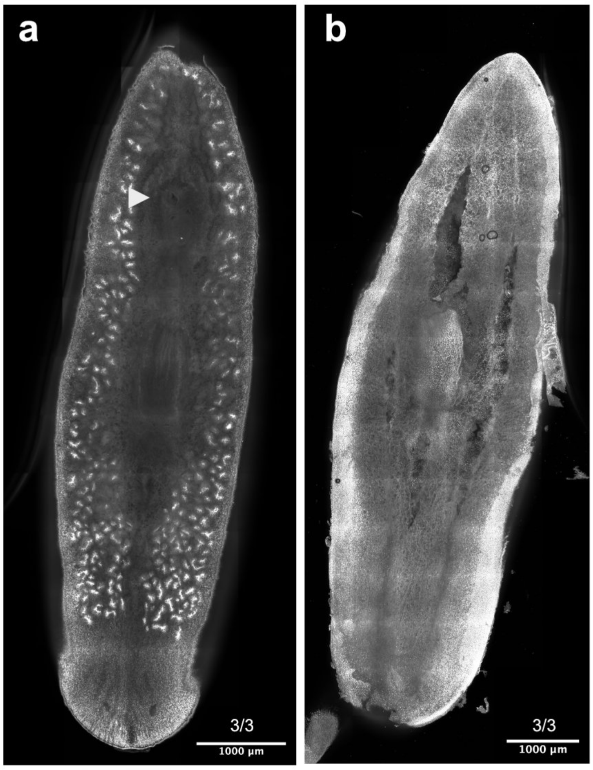

Supplementary Fig. 25 DAPI staining of representative samples from the (a) *eGFP* RNAi and (b) *ophis* RNAi treatments. Three specimens were imaged from each condition. (a) shows a single slice of the image stack with a view of the abundant testes. (b) is a maximum intensity projection of the entire stack showing the lack of testes. White arrow in (a) highlights the copulatory apparatus, which is absent in (b).

Differential Gene Expression Analysis

We observed that, as expected, the *eGFP* RNAi control and the sexual wild-type clustered in the PCA (Supplementary Fig. 26a). Only three genes were detected as significantly down-regulated and six as significantly up-regulated in the eGFP RNAi vs sexual wild-type contrast (Supplementary Fig. 26b). Therefore, we grouped these two controls for the entire analysis. Next, we confirmed that *ophis* RNAi indeed knocked down the gene (Supplementary Fig. 26c, Supplementary Table 8) and then defined genes upregulated in the sexual control (wild-type & eGFP RNAi) vs. *ophis* RNAi and sexual control vs. asexuals wild-type (Supplementary Fig. 26d-g) as high-confidence reproduction-related genes. Enrichment analysis of these 2,136 reproduction-related genes revealed 30 GO terms that were statistically significantly enriched. 9 of these terms were related to cilium assembly or other cilium related terms, likely due to an enrichment in sperm-related processes in animals with developed testes (Supplementary Fig. 27, Supplementary Fig. 28). Using these data we clearly see that identification of reproduction-related genes via simple comparison of sexual and asexual wild-type would lead to the identification of genes that are also differentially expressed between ophis RNAi and asexual wild type. I.e., genes that are likely different between strains – largely independent of their role in the reproductive system. Indeed, we find 1,829 genes that are upregulated in both these contrasts, but also 1,213 that are downregulated. Enrichment analysis of these strain-enriched genes, showed enrichment in GO terms related to immunity (Supplementary Fig. 29, Supplementary Fig. 30).

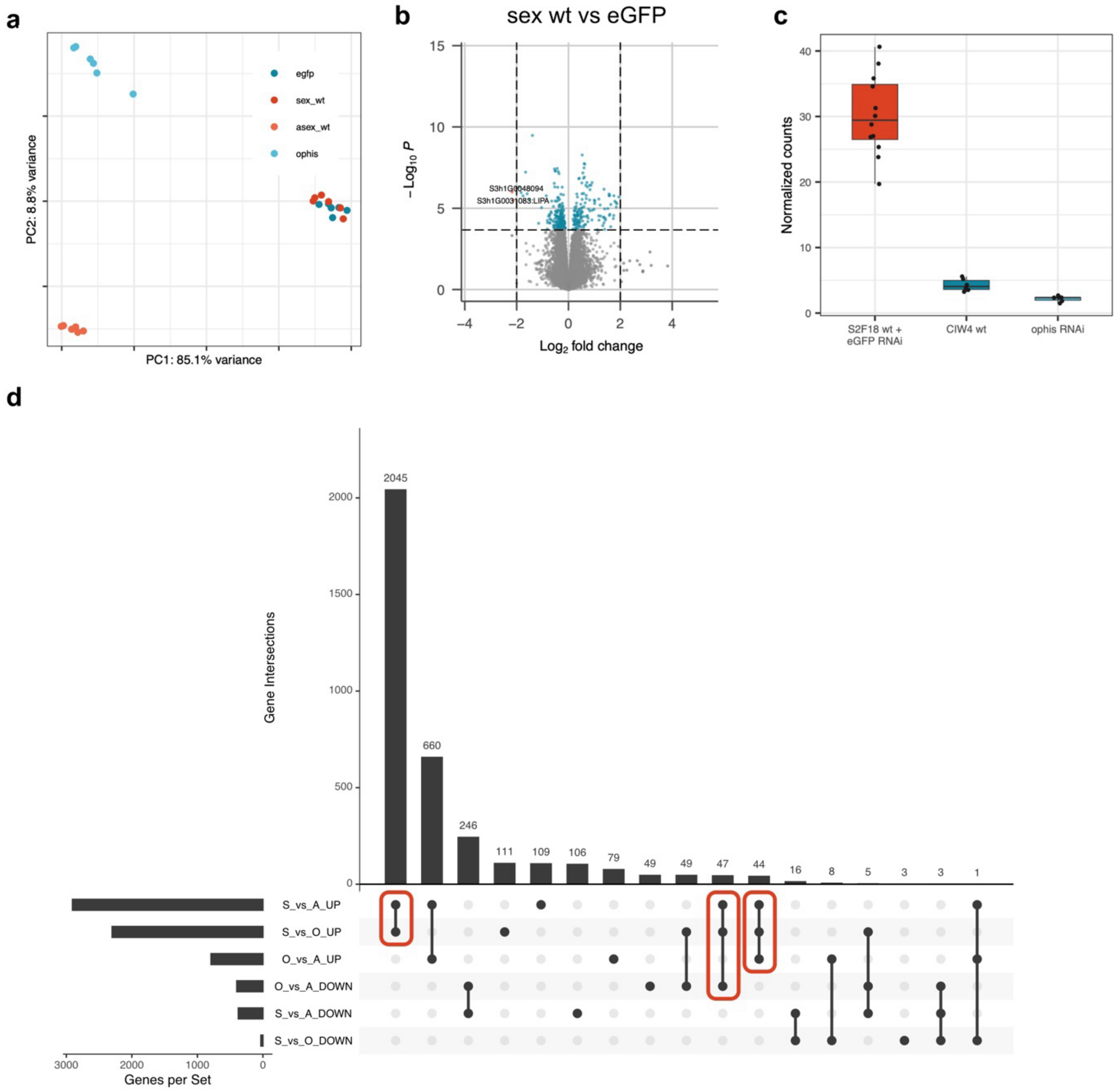

Supplementary Fig. 26 Identification of reproduction-related genes using RNA interference (RNAi) and differential expression analysis. **a** Principal component analysis of the expression of the four treatment groups: *eGFP* RNAi, *ophis* RNAi, LabSex wild-type, and LabAsex wild-type, each with six replicates. Minimum expression filtered counts were normalized, and log2 transformed using the trimmed mean of the M-values (TMM) method and regression using the voom function of the R package limma. **b** Volcano plot from differential expression analysis between sexual wild-type and *eGFP* RNAi, showing minimal differences (limma moderated t-test, two-sided, Benjamini-Hochberg correction for multiple comparisons; significance thresholds: $|\log2FC| \geq 2$, adjusted $p < 0.01$) **c** Normalized read counts for the RNAi target *ophis* (S3h1G0033988) in sexual control, asexual wild type, and *ophis* RNAi, indicating successful knock-down. Boxes indicate the interquartile range (IQR), with the median as a horizontal line. Whiskers extend to 1.5×IQR, and individual points represent raw data. **d** Upset plot summarizing the results from differential expression calls in Fig. 2d-f of the main text. Red boxes outline the sets designated as high-confidence reproduction-related transcripts.

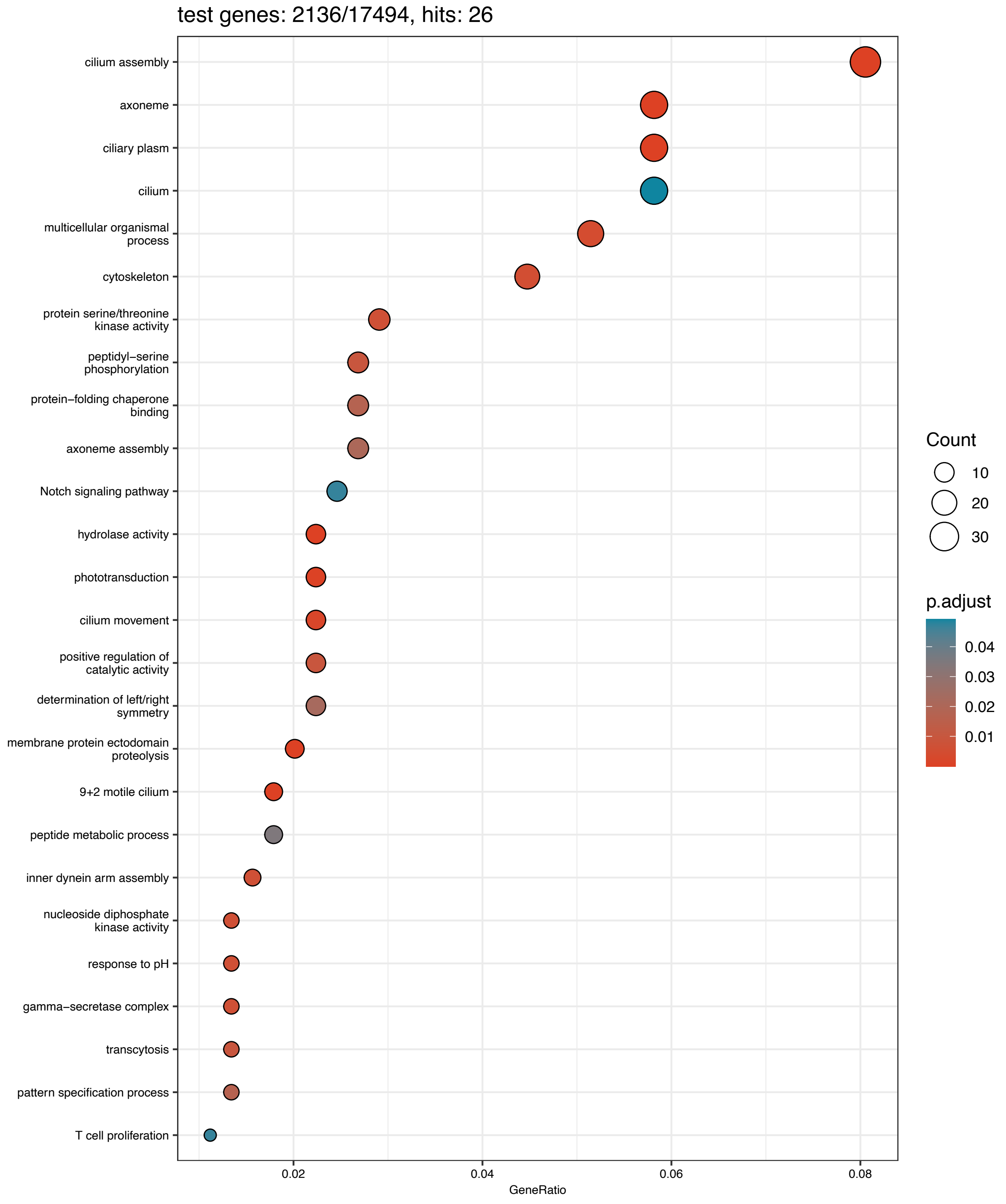

Supplementary Fig. 27 Enrichment dotplot showing GeneRatio and number of enriched genes for the 30 statistically significantly enriched GO terms in the set of high-confidence reproduction related genes.

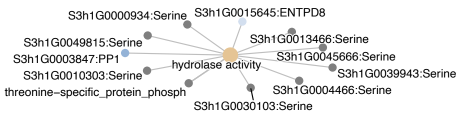

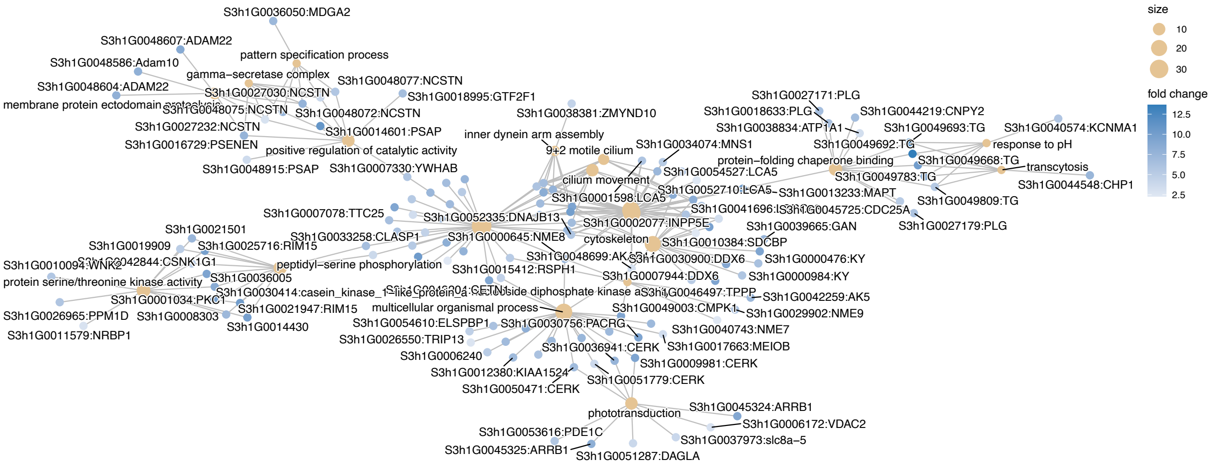

Supplementary Fig. 28 Gene-Concept Network plot showing the enriched GO terms and the associated genes for the set of high-confidence reproduction-related genes.

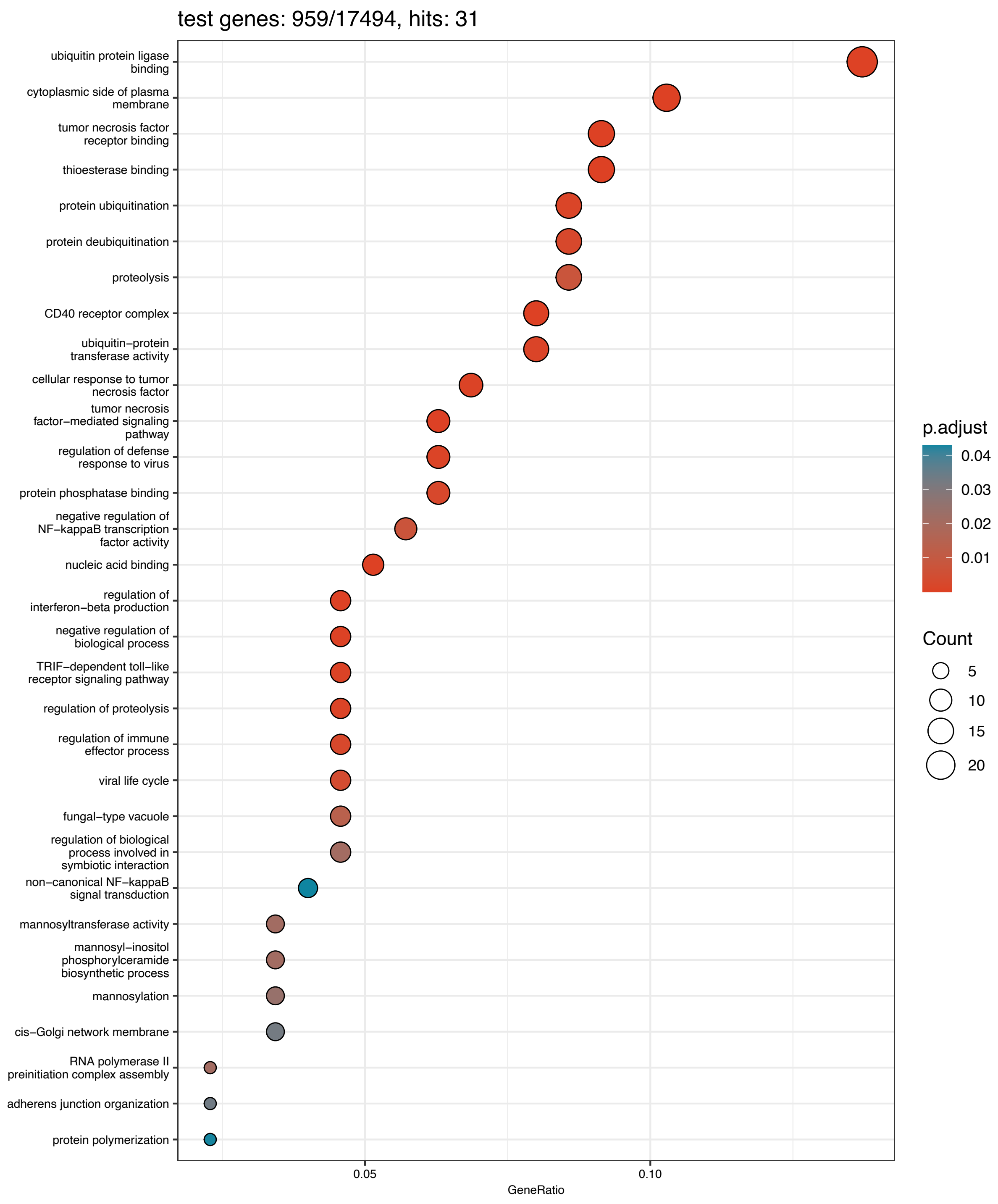

Supplementary Fig. 29 GO enrichment analysis of transcripts identified as enriched in one strain, but not due to the reproductive system.

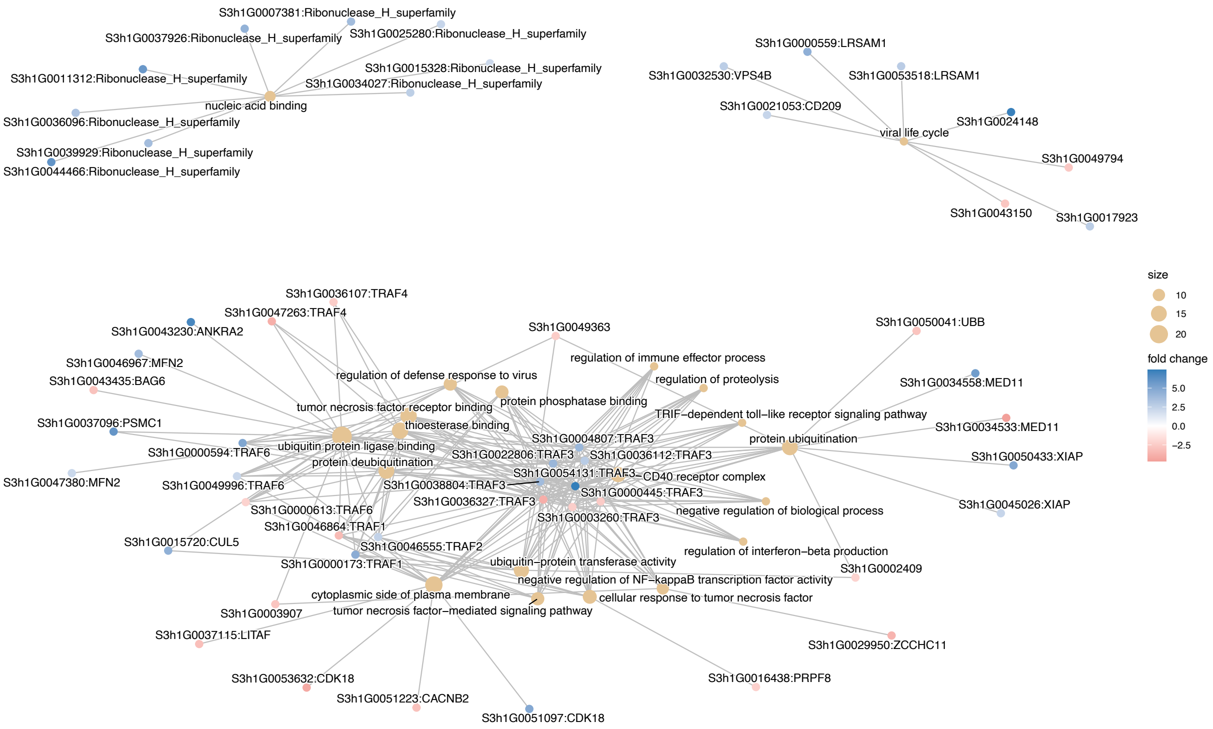

Supplementary Fig. 30 Gene-Concept Network plot showing the 31 enriched GO terms and the associated genes for strain-enriched genes.

Supplementary Note 8 | Gene Loss

To probe for potential gene losses in the vicinity of the breakpoints or for possible proximity effects on the expression of important reproductive system development genes, we analyzed the 112 gene models within a 1 Mb window flanking the Chr1/3 translocation breakpoints. To systematically analyze potential gene losses, we used the whole-genome alignment-based annotation pipeline TOGA[9]. Since TOGA is reference annotation centric, we again chose haplotype 1 of the LabSex genome as our reference and improved upon our previous annotation of the sexual genome via a liftover of haplotype 2 (Supplementary Table 3). We then used our whole-genome alignments to identify genes that were flagged as lost or potentially lost in the LabAsex genome. In addition, we assessed the general conservation of genes using whole-genome alignments of the high-quality genomes of the three most closely related *Schmidtea* species—*S. polychroa*, *S. lugubris*, and *S. nova*[10].

We then intersected the TOGA results with our RNAi-based annotation of reproduction-related genes (see section reproduction-related genes). In addition, we annotated 76 genes known from the literature to be important for germline development to assess if they were in the vicinity of the genomic rearrangements (Supplementary Table 8).

With these data sets in hand, we analyzed 112 gene models within a 1 Mb window flanking the breakpoint. Of those, 9 were flagged as lost or potentially lost in both haplotypes of the asexual strain. However, the flagged genes were scattered rather than forming a contiguous block, none of them were conserved in the three sister species of *S. mediterranea* (Supplementary Fig. 31; Supplementary Table 9), and 7/9 overlapped repetitive regions. Together, these data suggest that the ”lost” genes are likely repetitive elements and that the Chr1/3 translocation did not lead to additional gene deletion in the vicinity (Supplementary Table 9). In terms of reproduction-related genes, 13 mapped to within 1 Mb of the break point.

Because the Chr1/3 translocation was not associated with loss of reproduction-related genes, we next investigated whether the other large structural variations were accompanied by gene disruptions. Specifically, we examined the large inversions on Chr1 and Chr2, while ignoring the rearrangement on Chr4 due to possible issues with

the scaffolding quality (Supporting Information: Hi-C Chromosome 4). In the 1 Mb regions flanking the breakpoint of inv(1)(10M_325M), we identified 2/23 genes as potentially lost, but neither one was included in the set of reproduction-related genes (Supplementary Table 9). One of the lost genes was not conserved in *Schmidtea*, while the other was conserved but occurred within a tandem array of three gene copies, suggesting a recent duplication (Supplementary Table 9; Supplementary Fig. 32). Examining the asexual-specific Chr2 inversion, we found 5/65 genes near the breakpoints flagged as lost or potentially lost. One of them was annotated as reproduction-related but none were conserved across the genus, and three were in regions highly enriched for repeats. In addition to the potential gene losses, we detected an exon loss in one gene within LabAsex haplotype 2 (Supplementary Table 9; Supplementary Fig. 32). Two candidates are currently under investigation via knock-down assays.

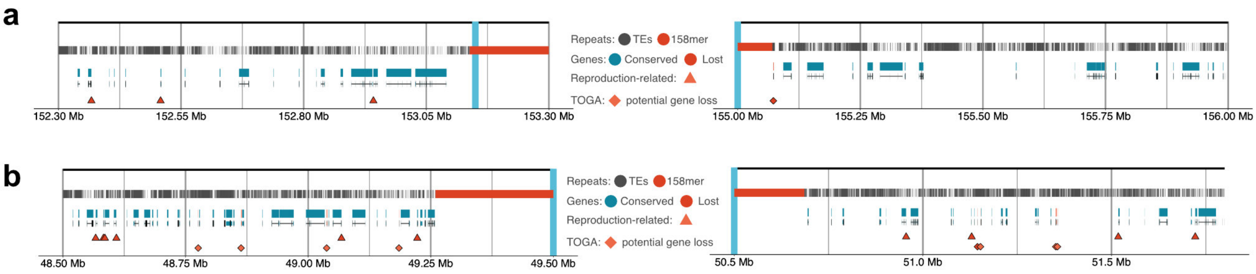

Supplementary Fig. 31 Gene loss analysis in the homologous regions of the Chr1/3 translocation breakpoints in the LabSex assembly **a-b** Enlarge view of the regions outlined in Supplementary Fig. 3 a-b of the main text, showing the distribution of transposable elements (TEs), the 158mer repeats (158mer), and gene annotations. Triangles indicate high-confidence reproduction-related genes, and diamonds mark genes flagged as potentially lost in both haplotypes of the LabAsex assembly using the gene annotation pipeline TOGA.

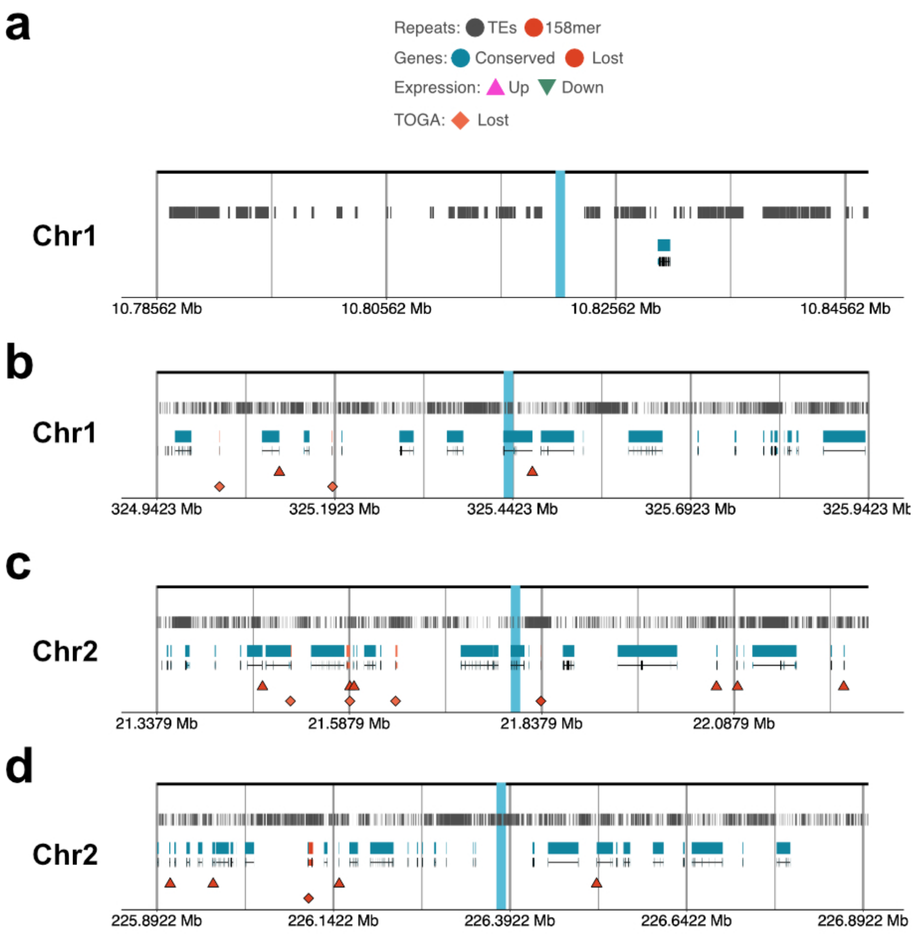

Supplementary Fig. 32 Genomic context on the LabSex assembly haplotype 1 of chromosome inversion breakpoints of the Chr1 Inversion 1 (a-b) and Chr2 inversion (c-d). Shown are the distribution of transposable elements (TEs) and gene annotations. Triangles indicate high-confidence reproduction-related genes, and diamonds mark genes flagged as potentially lost in both haplotypes of the LabAsex assembly using the gene annotation pipeline TOGA.

Supplementary Note 9 | Population Genomics

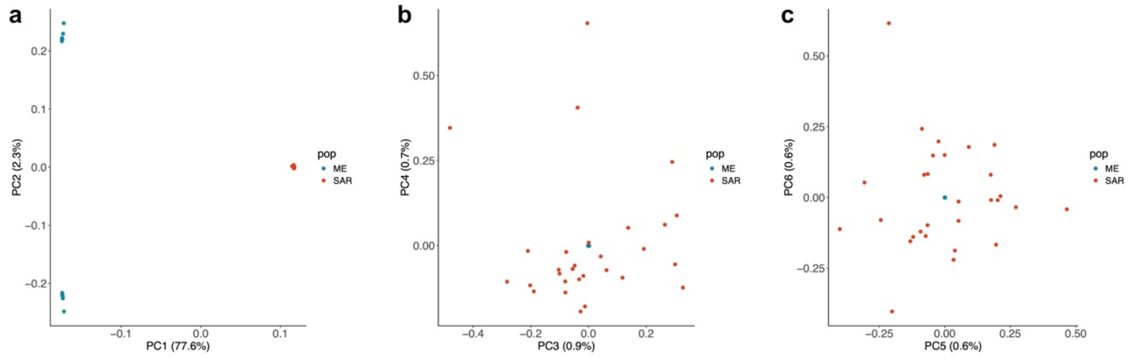

Supplementary Fig. 33 Principal component (PC) analysis of whole-genome sequencing data of individuals from Menorca (ME) and Sardinia (SAR) populations. Shown are (a) PC1 & PC2, (b) PC3 & PC4, and (c) PC5 & PC6.

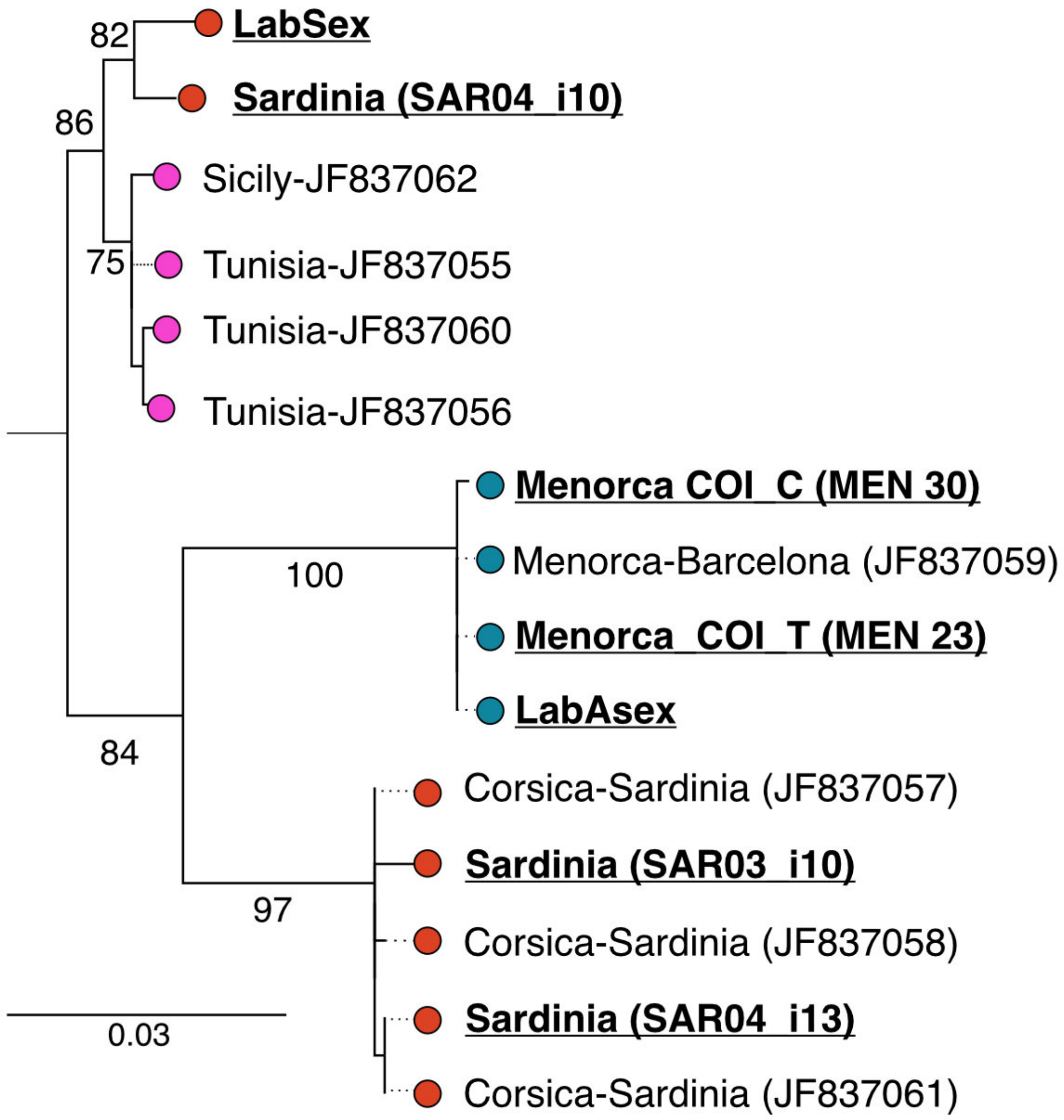

Supplementary Fig. 34 Maximum-likelihood phylogeny of representative sequences of the Sardinia population (SarSex), the Menorca population (MenAsex), the asexual laboratory strain from Barcelona (LabAsex), the sexual laboratory strain from Sardinia (LabSex), and publicly available sequences of a fragment of the COI barcoding gene. We find high similarity between MenAsex and LabAsex and more diversity within SarSex, which does not appear monophyletic. However, note the low node support, which is based on 100 nonparametric bootstrap samples. As an outgroup we used the COI sequence of *S. polychroa* extracted from the dd_Spol_v4 transcriptome available from Planmine (contig Spol_v4_43147_1_1). All sequences were trimmed to the length of the barcoding region provided by Larao et al. [11].

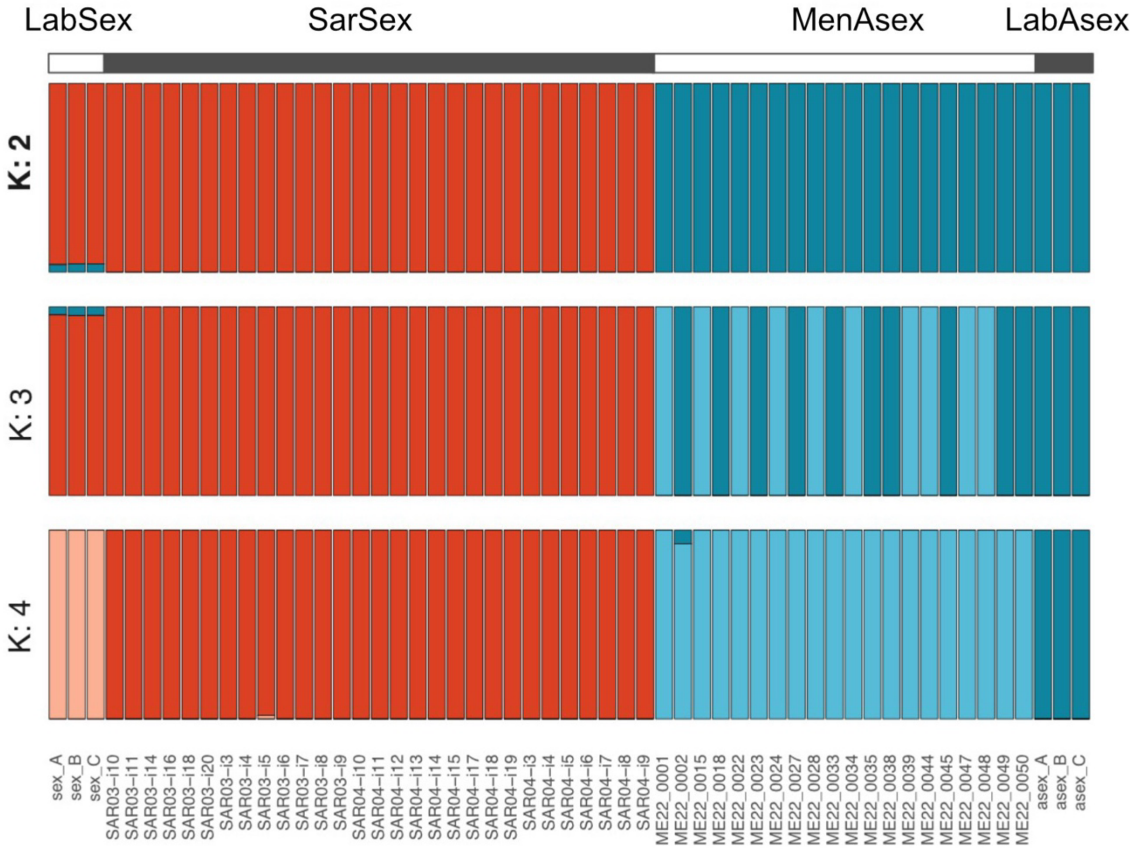

Supplementary Fig. 35 Population structure inferred using ngsAdmix. Best K = 2 was identified using Evanno's method.

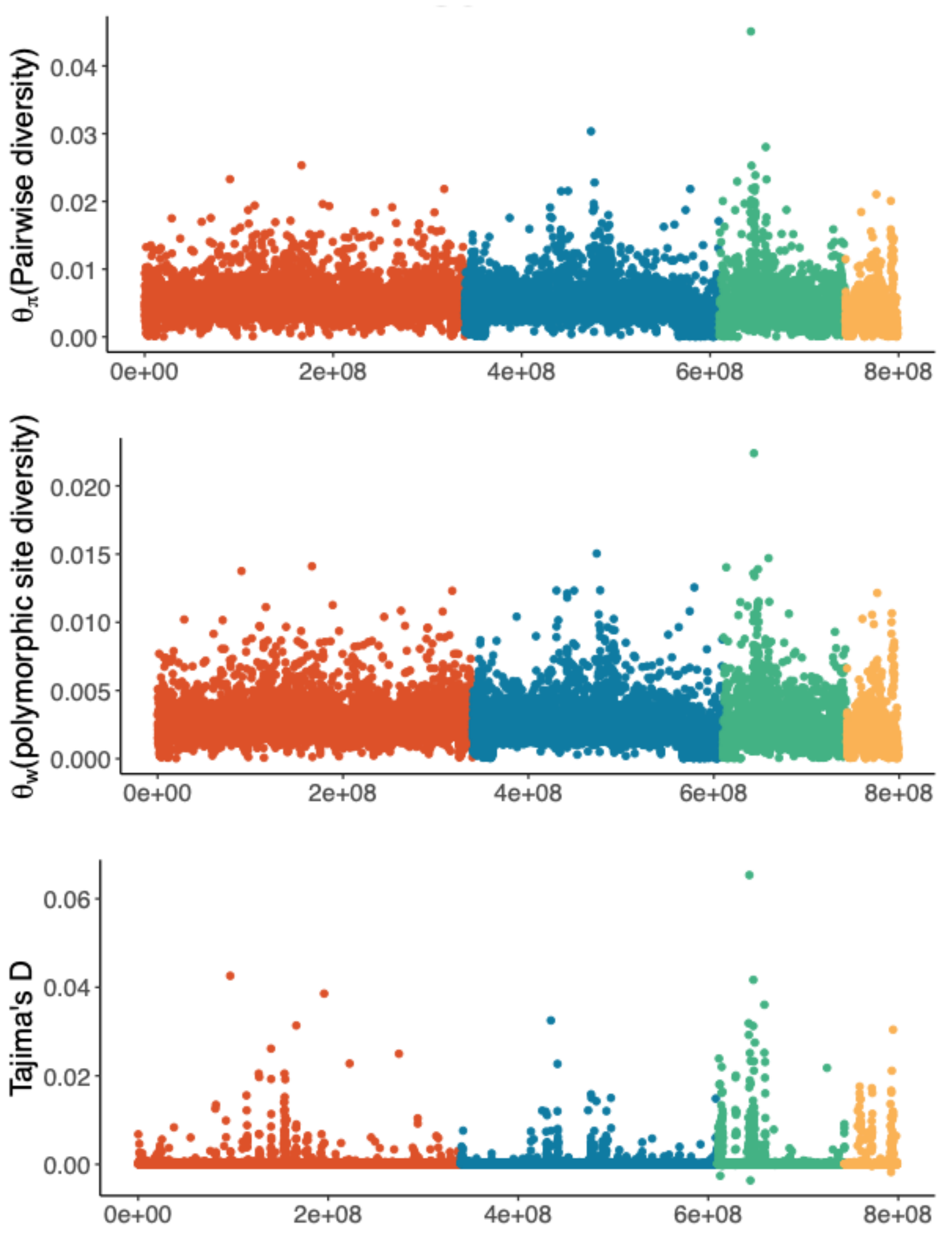

Supplementary Fig. 36 Population genomic parameters for the asexual population from Menorca (MenAsex). Show are theta estimates based on nucleotide diversity, polymorphic site distribution and Tajima's D inferred for 50Kb windows across the chromosome scaffolds.

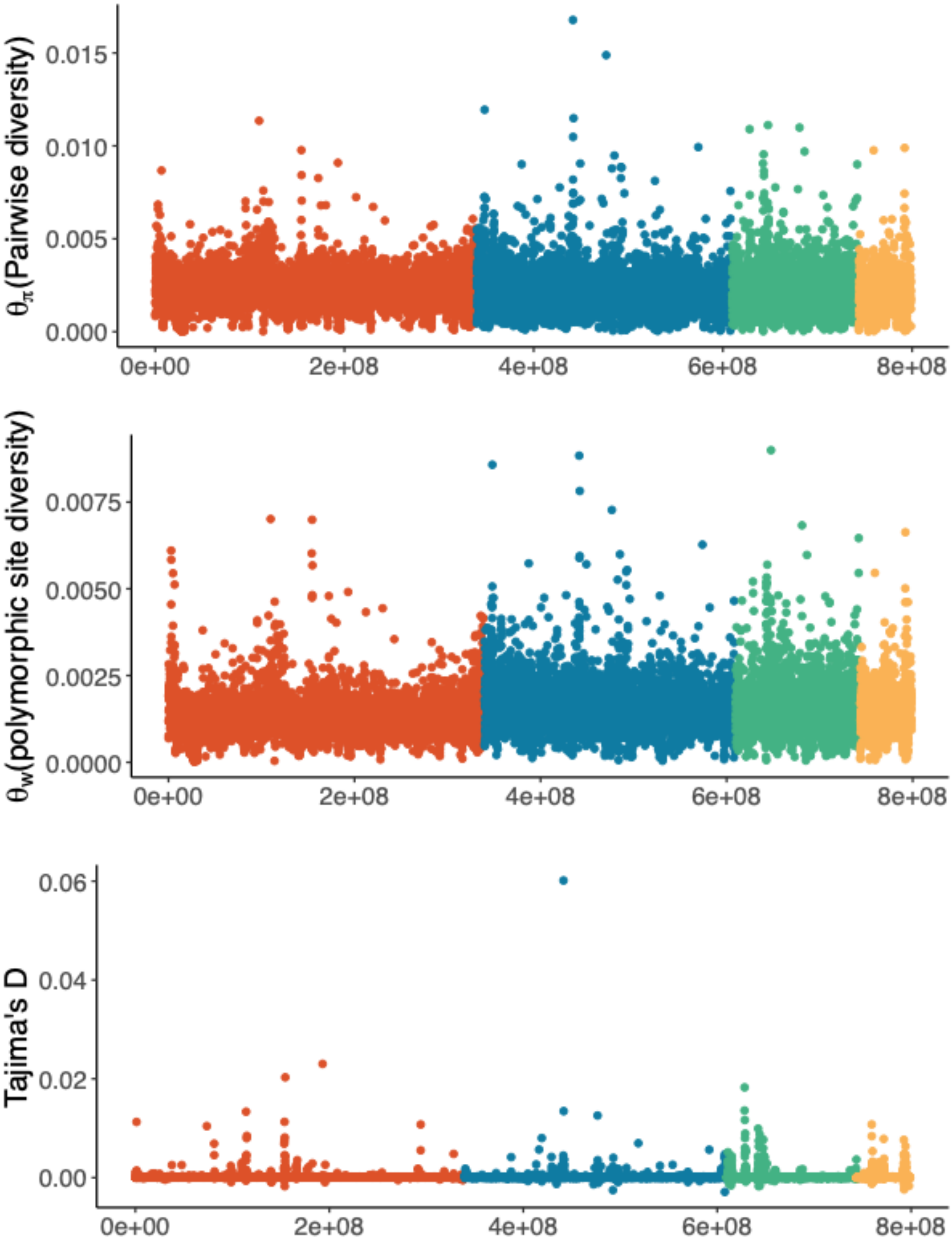

Supplementary Fig. 37 Population genomic parameters for the asexual population from Sardinia (SarSex). Show are theta estimates based on nucleotide diversity, polymorphic site distribution and Tajima's D inferred for 50Kb windows across the chromosome scaffolds.

Supplementary Table 8 Mean Diversity (π) and Watterson (W) estimators of the population parameter theta (θ) in SarSex and MenAsex, both genome-wide and for each chromosome scaffold. The last column indicates the grouping of chromosomes according to the test presented in the table below.

θ estimator	Population	Median	Mean	SD	Chromosome	Wilcoxon group
π	SarSex	0.00229	0.00233	0.00087	chr1	A
π	SarSex	0.00209	0.00219	0.00110	chr2	B
π	SarSex	0.00208	0.00222	0.00120	chr3	B
π	SarSex	0.00213	0.00218	0.00105	chr4	B
π	SarSex	0.00219	0.00225	0.00103	genome-wide	
π	MenAsex	0.00463	0.00504	0.00219	chr1	A
π	MenAsex	0.00415	0.00449	0.00246	chr2	B
π	MenAsex	0.00383	0.00441	0.00316	chr3	C
π	MenAsex	0.00268	0.00319	0.00270	chr4	D
π	MenAsex	0.00429	0.00463	0.00254	genome-wide	
W	SarSex	0.00121	0.00129	0.00053	chr1	A
W	SarSex	0.00148	0.00157	0.00066	chr2	B
W	SarSex	0.00153	0.00163	0.00074	chr3	C
W	SarSex	0.00144	0.00151	0.00065	chr4	D
W	SarSex	0.00136	0.00146	0.00064	genome-wide	
W	MenAsex	0.00239	0.00264	0.00125	chr1	A
W	MenAsex	0.00213	0.00238	0.00137	chr2	B
W	MenAsex	0.00204	0.00240	0.00174	chr3	C
W	MenAsex	0.00154	0.00185	0.00156	chr4	D
W	MenAsex	0.00221	0.00246	0.00142	genome-wide	

Supplementary Table 9 Wilcoxon test showing that the SarSex population has significantly lower values of θ_π and θ_W than the MenAsex population. Tests are two-sided.

θ estimator	group1	group2	n1	n2	statistic	p
π	SarSex	MEN	15752	15463	36511700	< 2.2e-16
W	SarSex	MEN	15752	15463	53283438	< 2.2e-16

Supplementary Table 10 All pairwise Wilcoxon tests for differences in θ_π and θ_W between chromosomes. All tests are two-sided and corrected for multiple testing using the Benjamini-Hochberg procedure.

**θ estimator**	**Population**	**Group1**	**Group2**	**n1**	**n2**	**Statistic**	**p**	**p.adj**	**signif**
π	SarSex	chr1	chr2	6717	5307	20158263	4.7E-35	2.82E-34	****
π	SarSex	chr1	chr3	6717	2649	9956697	2.37E-19	7.11E-19	****
π	SarSex	chr1	chr4	6717	1079	4038495	1.52E-09	3.04E-09	****
π	SarSex	chr2	chr3	5307	2649	7018898	0.916	0.916	ns
π	SarSex	chr2	chr4	5307	1079	2830526	0.555	0.833	ns
π	SarSex	chr3	chr4	2649	1079	1417973	0.708	0.85	ns
W	SarSex	chr1	chr2	6717	5307	12440432	2E-178	1.2E-177	****
W	SarSex	chr1	chr3	6717	2649	6013554	3.6E-132	1.1E-131	****
W	SarSex	chr1	chr4	6717	1079	2740903	6.98E-38	1.4E-37	****
W	SarSex	chr2	chr3	5307	2649	6735214	0.002	0.003	**
W	SarSex	chr2	chr4	5307	1079	3006429	0.009	0.009	**
W	SarSex	chr3	chr4	2649	1079	1555912	0.000021	3.15E-05	****
π	MenAsex	chr1	chr2	6654	5255	20512606	1.88E-59	2.82E-59	****
π	MenAsex	chr1	chr3	6654	2548	10576677	1.06E-75	2.12E-75	****
π	MenAsex	chr1	chr4	6654	1006	5179419	6.8E-173	4.1E-172	****
π	MenAsex	chr2	chr3	5255	2548	7258113	1.58E-09	1.58E-09	****
π	MenAsex	chr2	chr4	5255	1006	3685260	1.38E-87	4.14E-87	****
π	MenAsex	chr3	chr4	2548	1006	1673004	8.91E-46	1.07E-45	****
W	MenAsex	chr1	chr2	6654	5255	20207816	1.96E-48	2.94E-48	****
W	MenAsex	chr1	chr3	6654	2548	10177919	2.64E-50	5.28E-50	****
W	MenAsex	chr1	chr4	6654	1006	4842587	7.6E-116	4.6E-115	****
W	MenAsex	chr2	chr3	5255	2548	7053488	0.000122	0.000122	***
W	MenAsex	chr2	chr4	5255	1006	3454871	7.28E-54	2.18E-53	****
W	MenAsex	chr3	chr4	2548	1006	1599602	8.46E-31	1.02E-30	****

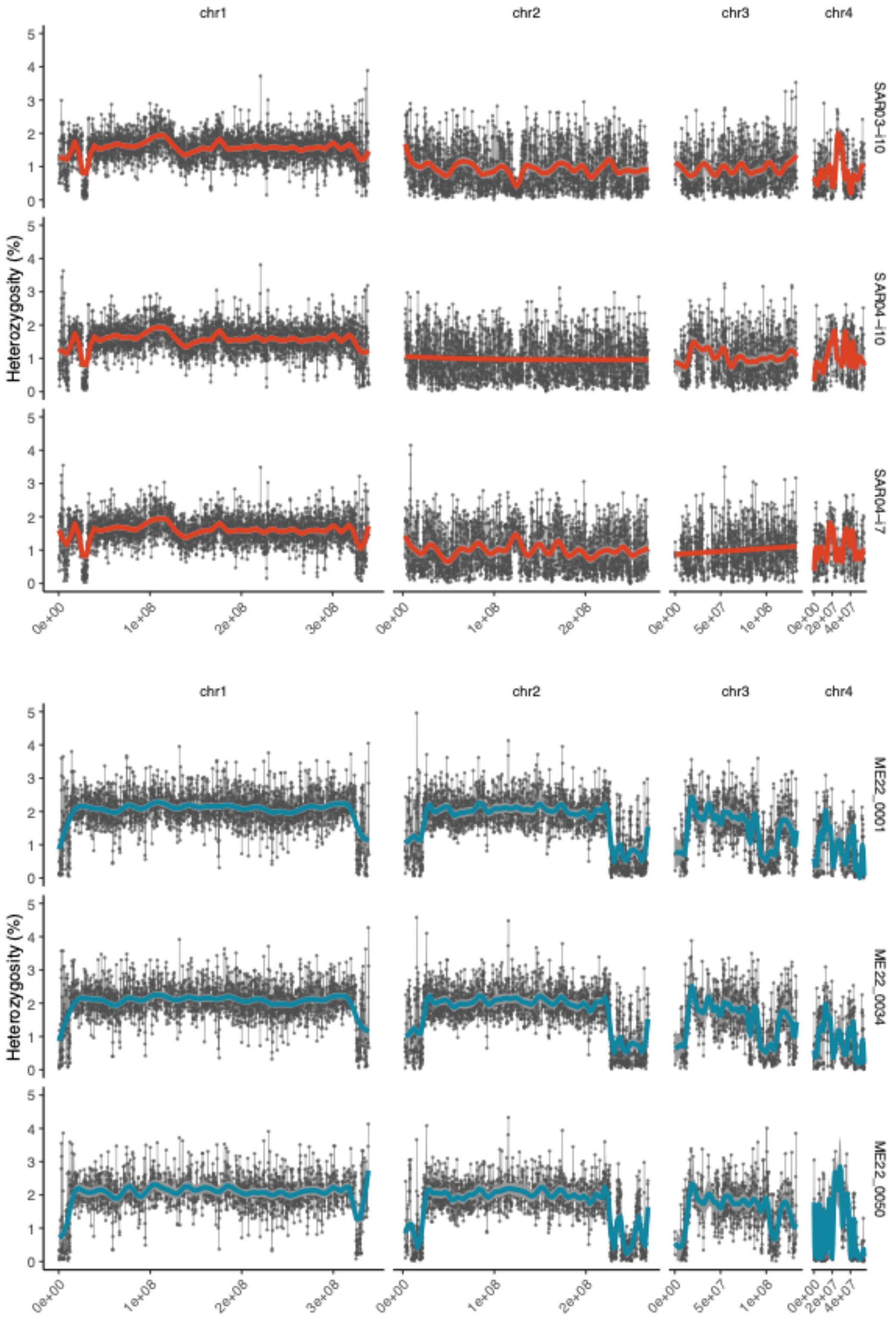

Supplementary Fig. 38 Observed heterozygosity calculated as the percentage of heterozygous sites per called site (min 10 reads, FS < 30). Points show value within a 100kb window and are connected by thin lines. The colored lines show the fit of a general additive model (GAM) the r package ggplot2. The fit was calculated using the formula ‘y ~ s(x, k = 50)’. Envelope around the colored lines represent 95% confidence intervals. Red: three samples from the SarSex population; blue: three samples from the MenAsex population.

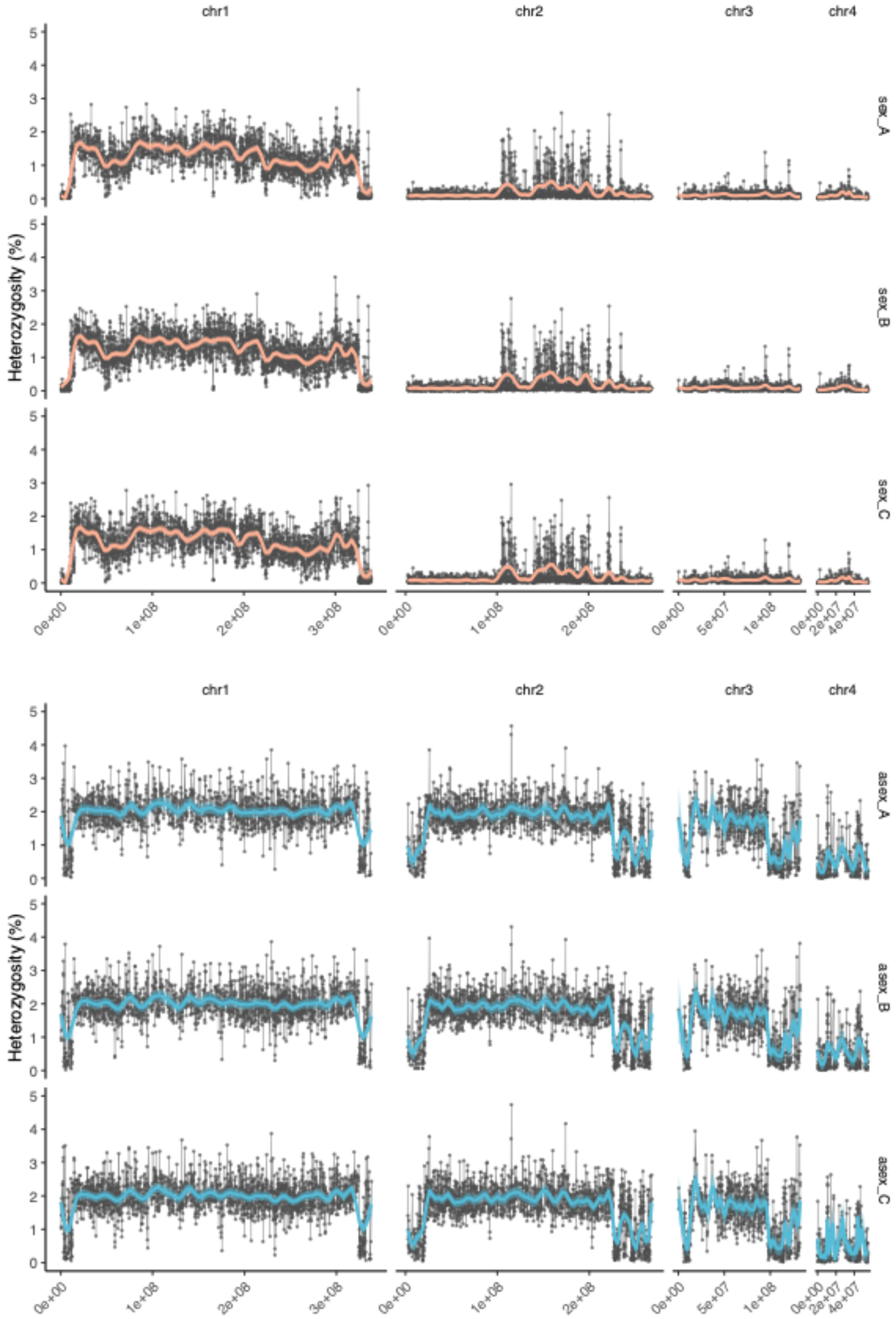

Supplementary Fig. 39 Observed heterozygosity calculated as the percentage of heterozygous sites per called site (min 10 reads, FS < 30). Points show value within a 100kb window and are connected by thin lines. The colored lines show the fit of a general additive model (GAM) fit via the r package ggplot2. The fit was calculated using the formula 'y ~ s(x, k = 50)'. Envelope around the colored lines represent 95% confidence intervals. Red: three samples from the LabSex strain; blue: three samples from the LabAsex strain.

Supplementary Note 10 | Dataset Intersection

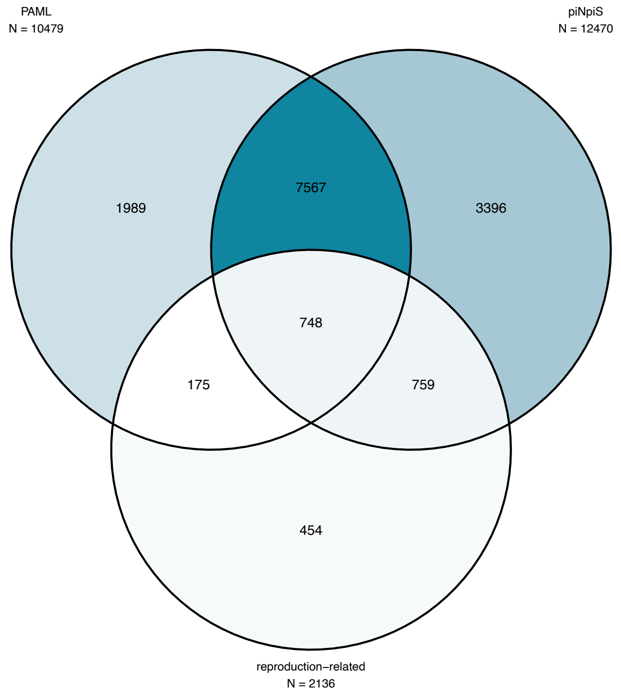

Supplementary Fig. 40 Venn diagram showing the overlap between genes used in the dN/dS analysis (PAML), the piN/piS analysis(piNpiS), and genes annotated as reproduction-related.

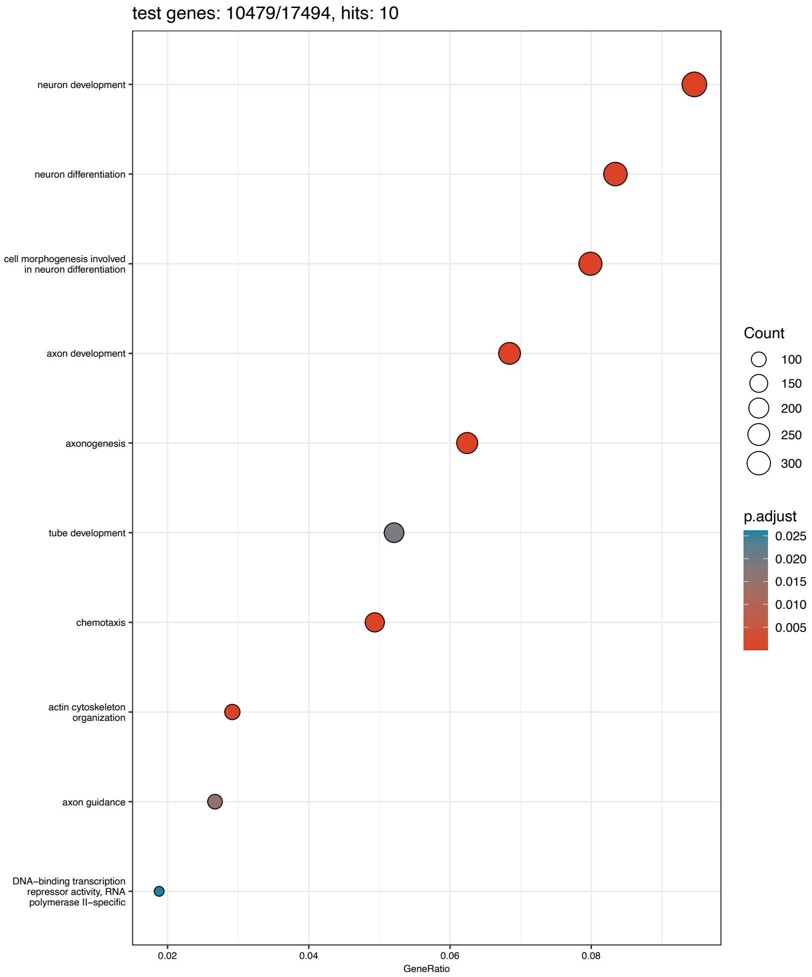

Supplementary Fig. 41 Enrichment dotplot showing GeneRatio and number of enriched genes for the 10 statistically significantly enriched GO terms in the set of single-copy genes used for the d_N/d_S analysis.

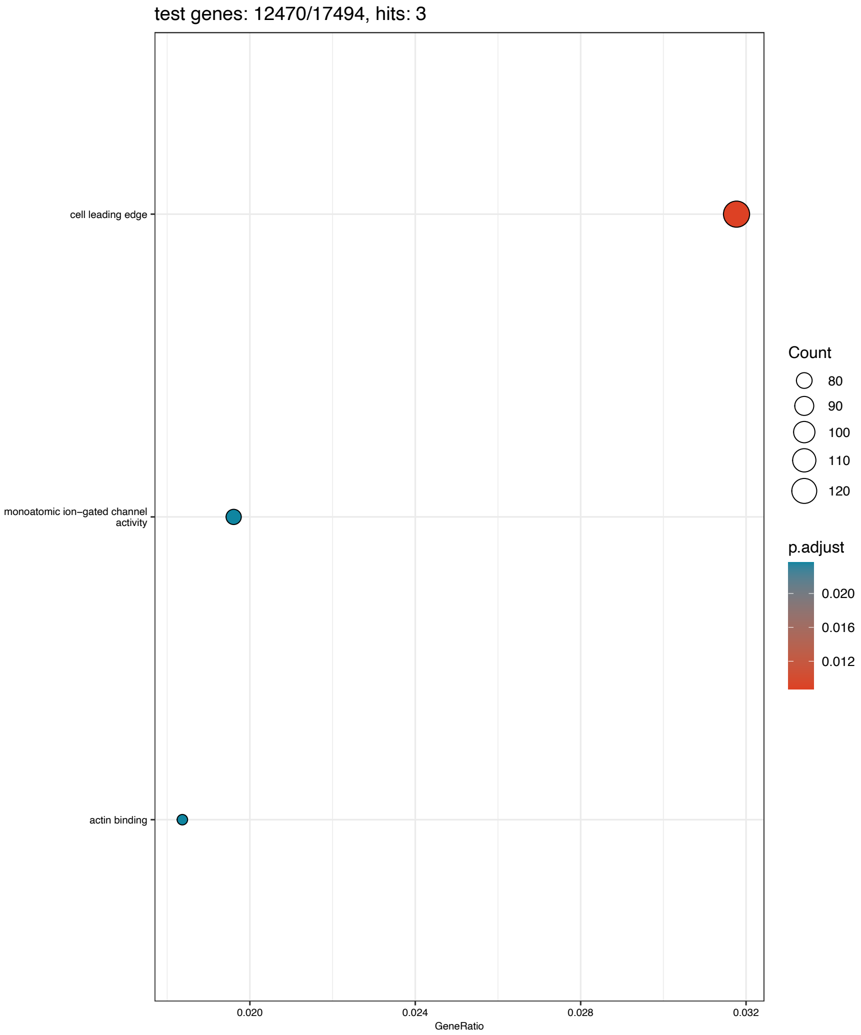

Supplementary Fig. 42 Enrichment dotplot showing GeneRatio and number of enriched genes for the 3 statistically significantly enriched GO terms in genes used for the piN/piS analysis.

Supplementary Note 11 | LTR Insertion Age

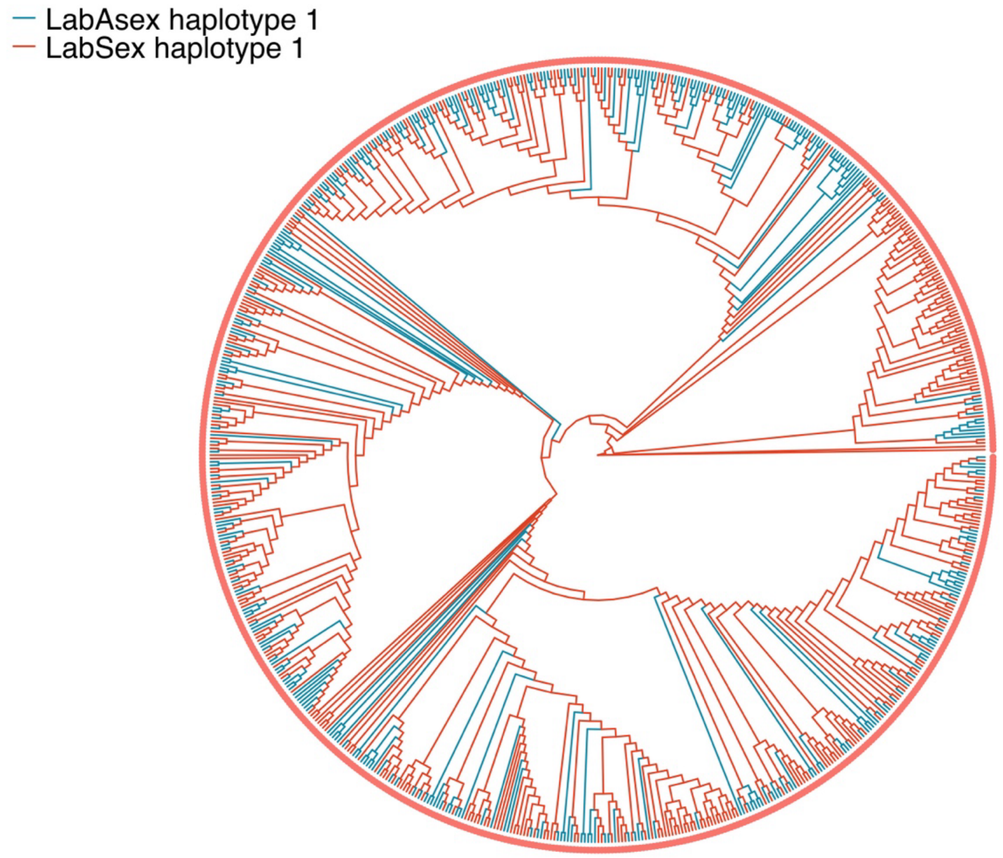

Supplementary Fig. 43 Phylogenetic tree of LTR transposons identified to have inserted exclusively in the asexual LabAsex (schMedA2) and sexual LabSex (schMedS3) genomes.

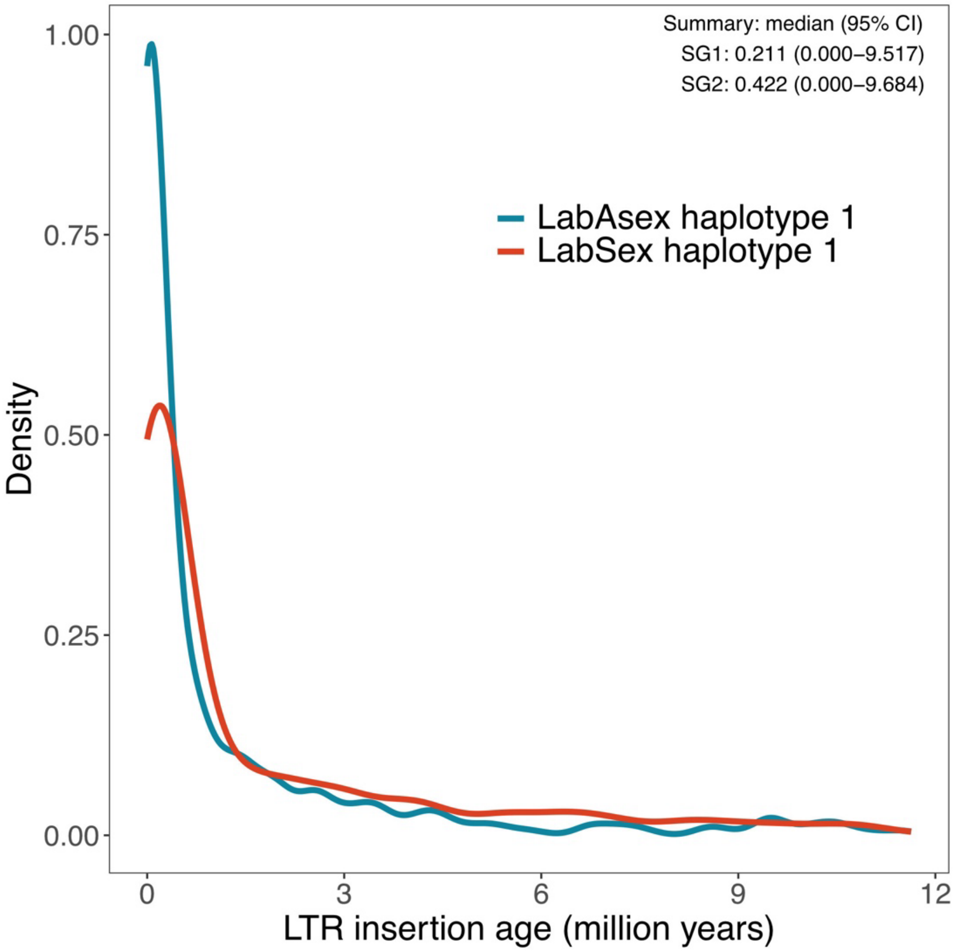

Supplementary Fig. 44 Density plot showing the distribution of divergence between LTR copies. Median insertion age is 0.211 Ma and 0.422 Ma in the asexual and sexual genome, respectively.

References

1. Li, H. New strategies to improve minimap2 alignment accuracy. *Bioinforma. Oxf. Engl.* **37**, 4572–4574 (2021).
2. Steinegger, M. & Söding, J. MMseqs2 enables sensitive protein sequence searching for the analysis of massive data sets. *Nature Biotechnology* https://www.nature.com/articles/nbt.3988 (2017) doi:10.1038/nbt.3988.
3. Pertea, G. & Pertea, M. GFF Utilities: GffRead and GffCompare. *F1000Research* **9**, 304 (2020).
4. Pardo-Palacios, F. J. *et al.* SQANTI3: curation of long-read transcriptomes for accurate identification of known and novel isoforms. *Nat. Methods* 1–5 (2024) doi:10.1038/s41592-024-02229-2.
5. Wolff, J. *et al.* Galaxy HiCExplorer 3: a web server for reproducible Hi-C, capture Hi-C and single-cell Hi-C data analysis, quality control and visualization. *Nucleic Acids Res.* **48**, W177–W184 (2020).
6. Saberi, A., Jamal, A., Beets, I., Schoofs, L. & Newmark, P. A. GPCRs Direct Germline Development and Somatic Gonad Function in Planarians. *PLOS Biol.* **14**, e1002457 (2016).
7. Issigonis, M. & Newmark, P. A. From worm to germ: Germ cell development and regeneration in planarians. in *Current Topics in Developmental Biology* vol. 135 127–153 (Elsevier, 2019).
8. Davies, E. L. *et al.* Embryonic origin of adult stem cells required for tissue homeostasis and regeneration. *eLife* **6**, (2017).
9. Kirilenko, B. M. *et al.* Integrating gene annotation with orthology inference at scale. *Science* **380**, eabn3107 (2023).
10. Leria, L., Sluys, R. & Riutort, M. Diversification and biogeographic history of the Western Palearctic freshwater flatworm genus *Schmidtea* (Tricladida: Dugesiidae), with a redescription of *Schmidtea nova*. *J. Zool. Syst. Evol. Res.* **56**, 335–351 (2018).
11. Lázaro, E. M. *et al. Schmidtea mediterranea* phylogeography: an old species surviving on a few Mediterranean islands? *BMC Evol. Biol.* **11**, 274 (2011).
