# Supplementary material for: Stepwise emergence of recombination suppression precedes fissiparous asexuality in the planarian Schmidtea mediterranea
Source: Nat Commun. 2026 Jun 26;17:5588. doi: 10.1038/s41467-026-74605-9 (PMC13309565; doi:10.1038/s41467-026-74605-9)
Supplement: Supplementary file 3 — Reporting Summary [file 41467_2026_74605_MOESM3_ESM.pdf]

nature portfolio

Corresponding author(s): Jochen C. Rink

Last updated by author(s): Aug 8, 2025

Reporting Summary

Nature Portfolio wishes to improve the reproducibility of the work that we publish. This form provides structure for consistency and transparency in reporting. For further information on Nature Portfolio policies, see our Editorial Policies and the Editorial Policy Checklist.

Statistics

For all statistical analyses, confirm that the following items are present in the figure legend, table legend, main text, or Methods section.

n/a	Confirmed	
☐	☒	The exact sample size (*n*) for each experimental group/condition, given as a discrete number and unit of measurement
☒	☐	A statement on whether measurements were taken from distinct samples or whether the same sample was measured repeatedly
☐	☒	The statistical test(s) used AND whether they are one- or two-sided<br>*Only common tests should be described solely by name; describe more complex techniques in the Methods section.*
☒	☐	A description of all covariates tested
☐	☒	A description of any assumptions or corrections, such as tests of normality and adjustment for multiple comparisons
☐	☒	A full description of the statistical parameters including central tendency (e.g. means) or other basic estimates (e.g. regression coefficient) AND variation (e.g. standard deviation) or associated estimates of uncertainty (e.g. confidence intervals)
☐	☒	For null hypothesis testing, the test statistic (e.g. F, t, r) with confidence intervals, effect sizes, degrees of freedom and P value noted<br>*Give P values as exact values whenever suitable.*
☒	☐	For Bayesian analysis, information on the choice of priors and Markov chain Monte Carlo settings
☒	☐	For hierarchical and complex designs, identification of the appropriate level for tests and full reporting of outcomes
☐	☒	Estimates of effect sizes (e.g. Cohen's *d*, Pearson's *r*), indicating how they were calculated

Our web collection on statistics for biologists contains articles on many of the points above.

Software and code

Policy information about availability of computer code

Data collection	No software was used to collect data.

Data analysis	Custom code is available at https://doi.org/10.5281/zenodo.18415144<br>Genome assembly and scaffolding: hifiasm v0.7; SALSA v2; Arima SV pipeline v1.3; Juicer v1.6.99; BWA-MEM v0.7.17; Minimap2 v2.28<br>Hi-C processing and visualization: HiCUP v0.8.0; HiCExplorer v3.7.65; HiGlass v1.13.4; StainedGlass v0.6<br>Genome quality assessment: meryl v1.4.1; merqury v1.3; GenomeScope 2.0 v2.0.1; BUSCO v5.3.2<br>Genome annotation: eggNOG-mapper v2.1.10; InterProScan v5.54-87.0; DIAMOND v2.0.14; TransDecoder v5.7.1; MMseqs2 v16.747c62; GFFcompare v0.12.23; SQANTI3 v5.2.14; TOGA v1.1.7<br>Genome comparison and synteny: SyRI v1.6; GENESPACE v1.0.8; OrthoFinder v2.5.4; MCSanX v1.0.0<br>RNA-seq processing and differential expression: Trimmomatic v0.39; STAR v2.7.9a; edgeR v4.4.0; limma v3.62.1; EnhancedVolcano v1.24.0; ssizeRNA v1.3.3<br>Population genomics and variant calling: BCFtools v1.19; GATK v4.0; ANGSD v0.940; PCAngsd v1.10; NgsAdmix (commit a5cde05); CLUMPAK (initial release); ngsF-HMM v1.1.0; ngsLD v1.2.0; SMC++ v1.15.2<br>Recombination and selection analysis: genmap v1.3.0-2; SNPGenie v1.0; degenotate.py v1.3; PAML/CODEML v4.9; PAL2NAL v14.1; codonw v1.4.4<br>Phylogenetics: IQ-TREE v2.3.6; MAFFT v7.480; AMAS v1.02; PHYML v2.2.4; CAT v1.3<br>Statistical analysis: R v4.4.2; tidyverse v2.0.0; coin v1.4-3; effsize v0.8.1; boot v1.3-31; GenomicRanges v1.64.0<br>Contamination screening: Kraken2 v2.1.3<br>Cytogenetics: ISIS4 (METASystems)

For manuscripts utilizing custom algorithms or software that are central to the research but not yet described in published literature, software must be made available to editors and reviewers. We strongly encourage code deposition in a community repository (e.g. GitHub). See the Nature Portfolio guidelines for submitting code & software for further information.

Data

Policy information about availability of data

All manuscripts must include a data availability statement. This statement should provide the following information, where applicable:

- Accession codes, unique identifiers, or web links for publicly available datasets
- A description of any restrictions on data availability
- For clinical datasets or third party data, please ensure that the statement adheres to our policy

All sequencing data generated for this study have been deposited in the National Center for Biotechnology Information (NCBI) database. The schMedA2 LabAsex genome assembly is available under GenBank accessions JBMAJT000000000 (haplotype 1) and JBMAJU000000000 (haplotype 2). The schMedA2 genome gene and repeat annotation are available from Zenodo under accession code 18415144. Data used for genome assembly and annotation are available under BioProject accession PRJNA1289722. RNA-seq data used for the identification of reproduction-related genes are available under BioProject accession PRJNA1289391. Population genomics data for Schmidtea mediterranea are available under BioProject accession PRJNA1287507. Source data are provided as a Source Data file.

Research involving human participants, their data, or biological material

Policy information about studies with human participants or human data. See also policy information about sex, gender (identity/presentation), and sexual orientation and race, ethnicity and racism.

Reporting on sex and gender	*Use the terms sex (biological attribute) and gender (shaped by social and cultural circumstances) carefully in order to avoid confusing both terms. Indicate if findings apply to only one sex or gender; describe whether sex and gender were considered in study design; whether sex and/or gender was determined based on self-reporting or assigned and methods used.<br>Provide in the source data disaggregated sex and gender data, where this information has been collected, and if consent has been obtained for sharing of individual-level data; provide overall numbers in this Reporting Summary. Please state if this information has not been collected.<br>Report sex- and gender-based analyses where performed, justify reasons for lack of sex- and gender-based analysis.*
Reporting on race, ethnicity, or other socially relevant groupings	*Please specify the socially constructed or socially relevant categorization variable(s) used in your manuscript and explain why they were used. Please note that such variables should not be used as proxies for other socially constructed/relevant variables (for example, race or ethnicity should not be used as a proxy for socioeconomic status).<br>Provide clear definitions of the relevant terms used, how they were provided (by the participants/respondents, the researchers, or third parties), and the method(s) used to classify people into the different categories (e.g. self-report, census or administrative data, social media data, etc.)<br>Please provide details about how you controlled for confounding variables in your analyses.*
Population characteristics	*Describe the covariate-relevant population characteristics of the human research participants (e.g. age, genotypic information, past and current diagnosis and treatment categories). If you filled out the behavioural & social sciences study design questions and have nothing to add here, write "See above."*
Recruitment	*Describe how participants were recruited. Outline any potential self-selection bias or other biases that may be present and how these are likely to impact results.*
Ethics oversight	*Identify the organization(s) that approved the study protocol.*

Note that full information on the approval of the study protocol must also be provided in the manuscript.

Field-specific reporting

Please select the one below that is the best fit for your research. If you are not sure, read the appropriate sections before making your selection.

- [x] Life sciences
- [ ] Behavioural & social sciences
- [ ] Ecological, evolutionary & environmental sciences

For a reference copy of the document with all sections, see nature.com/documents/nr-reporting-summary-flat.pdf

Life sciences study design

All studies must disclose on these points even when the disclosure is negative.

Sample size	To determine the appropriate sample size and read depth for the RNA-seq experiment, we performed a power calculation using ssizeRNA single function from the R package ssizeRNA. We oriented our projected number of differentially expressed genes based on the comparison of sexual and asexual wild-type data from Davies et al., 7 where, with an average read count of 20 million per library, 52% of transcripts were significantly differentially expressed and 60% had a log2fold-change of >2. This determined that increasing the coverage to 40 million reads per library and a sample size of six would result in an acceptable beta of 0.72, assuming an alpha of 0.05.<br>For the population genomic analyses we sequence 20 and 29 individuals, since 20-30 samples is generally considered the desirable range for robust inference of allele frequencies (e.g., see 10.1111/1755-0998.12654 for a review).
Data exclusions	No data was excluded from the final analyses.
Replication	For the CIW4 HiC analyses we performed 4 independent experiments with independent biological replicates and library preparation. Since the HiC signal was highly similar, the data was grouped for the final analysis. For the Menorca animals we generated libraries from 2 biological replicates for each of 3 independent clonal lines. Details on the high similarity of the data is given in the Supporting information. Again data was pooled for the final comparison with CIW4.
Randomization	For the RNA-seq experiment biological replicates consisting of 10 animals were cultured in separate petri dishes. The dishes of the three treatments were houses in the same climate chamber, distributed in a balanced manner to avoid edge effects, and fed on the same schedule. for each species and fed in a staggered interval. Sample processing was also done balanced and batched.
Blinding	Blinding was not necessary for the bioinformatic analyses.

Reporting for specific materials, systems and methods

We require information from authors about some types of materials, experimental systems and methods used in many studies. Here, indicate whether each material, system or method listed is relevant to your study. If you are not sure if a list item applies to your research, read the appropriate section before selecting a response.

Materials & experimental systems

n/a	Involved in the study
x	Antibodies
x	Eukaryotic cell lines
x	Palaeontology and archaeology
	x Animals and other organisms
x	Clinical data
x	Dual use research of concern
x	Plants

Methods

n/a	Involved in the study
x	ChIP-seq
x	Flow cytometry
x	MRI-based neuroimaging

Animals and other research organisms

Policy information about studies involving animals; ARRIVE guidelines recommended for reporting animal research, and Sex and Gender in Research

Laboratory animals	Laboratory strain of the sexual biotype of Schmidtea mediterranea (S2F18, derived from S2F2, internal ID: GOE00500). Laboratory strain of the asexual biotype of S. mediterranea (CIW4, internal ID: GOE00071).
Wild animals	Asexual Schmidtea mediterranea were collected from a stream along Rafal Colom Road, Menorca, Spain (39.90317° N, 4.23302° E) in May 2022. Sexual S. mediterranea were collected from the Temo River, Sardinia, Italy (40.398667° N, 8.559492° E) in April 2024.
Reporting on sex	The studied animals are simultaneous hermaphrodites or asexual.
Field-collected samples	Schmidtea mediterranea specimens were collected in the field by gently brushing them from rocks. They where then transported in falcon tubes to the Animal Facility of the Max Planck Institute for Multidisciplinary sciences, where they were housed in climate controlled cubboards in Montjuice water at 10-20°C and fed with organic calve liver paste.

Ethics oversight	All experiments were conducted in accordance with German law and the ethical guidlines of the Max Planck Society. No special approval is required to work with flatworms.

Note that full information on the approval of the study protocol must also be provided in the manuscript.

Plants

Seed stocks	*Report on the source of all seed stocks or other plant material used. If applicable, state the seed stock centre and catalogue number. If plant specimens were collected from the field, describe the collection location, date and sampling procedures.*
Novel plant genotypes	*Describe the methods by which all novel plant genotypes were produced. This includes those generated by transgenic approaches, gene editing, chemical/radiation-based mutagenesis and hybridization. For transgenic lines, describe the transformation method, the number of independent lines analyzed and the generation upon which experiments were performed. For gene-edited lines, describe the editor used, the endogenous sequence targeted for editing, the targeting guide RNA sequence (if applicable) and how the editor was applied.*
Authentication	*Describe any authentication procedures for each seed stock used or novel genotype generated. Describe any experiments used to assess the effect of a mutation and, where applicable, how potential secondary effects (e.g. second site T-DNA insertions, mosiacism, off-target gene editing) were examined.*
