# Supplementary material for: Stepwise emergence of recombination suppression precedes fissiparous asexuality in the planarian Schmidtea mediterranea
Source: Nat Commun. 2026 Jun 26;17:5588. doi: 10.1038/s41467-026-74605-9 (PMC13309565; doi:10.1038/s41467-026-74605-9)
Supplement: Supplementary file 2 — Transparent Peer Review file [file 41467_2026_74605_MOESM2_ESM.pdf]

nature portfolio

Peer Review File

Stepwise emergence of recombination suppression precedes fissiparous asexuality in the planarian *Schmidtea mediterranea*

Corresponding Author: Professor Jochen Rink

This file contains all reviewer reports in order by version, followed by all author rebuttals in order by version.

Version 1:

Reviewer comments:

Reviewer #1

(Remarks to the Author)
I commend the authors for an excellent and carefully executed piece of work on a very interesting and timely topic. The manuscript presents a technically sophisticated and conceptually ambitious study that investigates the genomic underpinnings of asexual reproduction in Schmidtea mediterranea. The integration of long-read sequencing, Hi-C–based chromosomal architecture, cytogenetics, and population genomic analyses represents a major advance for the field. The resulting haplotype-phased genome assembly of the asexual strain CIW4 is of very high quality and will undoubtedly become an important resource for the planarian community. The paper also has broad relevance for evolutionary genomics, as it addresses fundamental questions about how recombination suppression and chromosomal rearrangements relate to the evolution of asexuality.

While the study is technically impressive and the data are of high quality, several key interpretations, particularly those linking chromosomal rearrangements to the origin of asexuality, remain correlative rather than causal. This distinction between correlation and causation underpins most of my concerns. The authors convincingly show that extensive structural rearrangements are present in the asexual genome and that they predate or accompany the loss of sex, but they do not provide direct evidence that these rearrangements caused the transition to fissiparous reproduction. In its current form, the paper somewhat overstates this causal link, and the discussion would benefit from a more balanced consideration of alternative explanations.

- Correlation versus causation:

The central model proposed—whereby stepwise recombination suppression progressively eroded the benefits of sex and facilitated the emergence of asexuality—is elegant but not fully demonstrated. The data clearly support that inversions and translocations occurred in a sexual ancestor and that recombination was reduced in affected regions. However, whether this suppression directly triggered the loss of sexual reproduction, or whether both phenomena resulted from other demographic or developmental factors, remains unresolved. The authors should temper the causal language throughout the manuscript and frame their model as one plausible scenario among several. Phrasing such as “may have facilitated” or “is consistent with” would more accurately reflect the evidence.

Moreover, it would strengthen the manuscript to explicitly test or at least discuss alternative hypotheses that could generate similar genomic patterns. For example, the heterozygosity and linkage disequilibrium signatures attributed to recombination suppression could also arise from demographic events, such as bottlenecks, isolation, or hybridization between divergent sexual populations. Hybrid origin in particular could produce highly heterozygous, structurally rearranged genomes and impaired meiosis, leading to secondary asexuality—a scenario that has been documented in many other asexual systems. The authors might also consider that the structural rearrangements could have evolved under local adaptation or drift in small island populations, with the loss of sex following later due to regulatory or epigenetic changes in reproductive genes. These possibilities do not contradict their data but offer alternative, potentially more parsimonious explanations that should be acknowledged.

- Gene loss in sexual genes:

An additional point concerns the interpretation of the gene loss analysis near chromosomal breakpoints. While the authors convincingly show that no coding genes are physically disrupted by inversions or translocations, the conclusion that these rearrangements have no functional impact may be premature. Even if genes remain intact at the sequence level, their regulatory function could have been altered through changes in chromatin topology, enhancer–promoter interactions, or higher-order gene regulatory networks. Given that the authors already generated high-quality Hi-C data, they have everything needed to explore whether these structural rearrangements modify the connectivity of regulatory domains around key reproductive genes. Integrating such chromatin interaction data with transcriptomic profiles could reveal whether changes in genome architecture correspond to altered expression dynamics rather than to physical gene loss. This would allow the authors to move beyond the absence of coding gene loss and directly assess whether genome restructuring has affected the functional regulation of sexual development pathways.

- Functional annotation:

Although the authors describe the use of eggNOG-mapper, InterProScan, and DIAMOND for gene annotation, they do not provide quantitative information on annotation completeness, such as the percentage of genes with functional annotation, GO terms, or orthology assignments (or at least I have not found them, but they may be reported in the supplementary perhaps). Reporting these summary statistics would considerably enhance the transparency and reproducibility of the work, as well as enable meaningful cross-species comparisons. Based on my experience, these annotation pipelines typically leave a substantial fraction of genes unannotated, particularly in non-model and compositionally biased genomes such as planarians. It would therefore be valuable for the authors to assess the proportion of unannotated genes and consider complementary approaches to improve functional annotation, such as recently developed methods based on large language models (eg, FANTASIA) or deep learning (eg, DeepGoPlus), for instance.

- Divergence times inference:

A further point worth considering concerns the reliability of the molecular dating analyses in the context of S. mediterranea's extreme structural genome dynamics. As shown by their previous work in Ivanković et al. (2024), planarian genomes exhibit exceptionally high rates of chromosomal rearrangement and a general lack of conserved synteny, even among closely related lineages. If synteny is largely uncoupled from evolutionary constraint in these animals, then clock-like assumptions based on sequence divergence alone may not accurately capture the true timing of lineage splits. In this sense, the inferred divergence between the sexual and asexual strains, already surprisingly recent, should be interpreted cautiously. The authors could strengthen this section by explicitly discussing how structural volatility and relaxed synteny conservation may affect the robustness of their dating estimates, and by exploring whether alternative calibration approaches (e.g., using gene families with conserved chromosomal context or benchmarking against more stable taxa) yield consistent results.

- Presentation and figures:

The manuscript is generally well written and well organized, though somewhat dense in parts and difficult to follow, particularly with the acronyms of the populations, etc. The figures are clear but could be simplified in places. Some secondary analyses might be moved to Supplementary Figures to enhance readability. Methods are described in commendable detail and appear sufficient for reproducibility, but sequencing depth per sample and the exact filtering thresholds for evolutionary analyses should be reported in the main text or figure legends, I feel that the authors refer to previous work too often (eg, genome assembly methods, etc.). On the other hand, there are parts, such as the genome assembly and annotation of the asexual strain in Results, that could go almost directly to Supplementary Material. The data availability statement should include accession numbers for all newly generated sequencing data to ensure transparency, which I'm sure will be fixed in later revisions.

Reviewer #2

(Remarks to the Author)
The authors present a phased and annotated genome of the asexual laboratory strain of Schmidtea mediterranea flatworms, along with an in-depth population-genomic study of wild asexual and sexual populations. The volume of genomic data and the thoroughness of the analyses is impressive. The conclusions about the history of the genomes and populations is largely persuasive. I have one substantive comment and a number of minor copy-editing suggestions:

Substantive comment

The suggestion that fissiparity can partially overcome the cost of sex by avoiding the one-cell bottleneck is an interesting one. Kondrashov made an opposite argument (Kondrashov, A. S. (1994). Mutation load under vegetative reproduction and cytoplasmic inheritance. Genetics, 137(1), 311–318.), which the authors do not cite. The authors should at least cite that article and ideally explain why it is invalid or does not apply.

Copy-editing suggestions

lines 67- 68, change “sexual reproduction, parthenogenesis” to sexual reproduction, to parthenogenesis”

line 124, change “mercury” to “merqury”
line 508, change, “which exhibit” to “which exhibits”
line 517, change “Rotifers” to “rotifers”
lines 595-596, is the reported degree of precision in coordinates of collection sites (to nearest 1m in Menorca and to nearest 10cm in Sardinia) justified? Typically, “N”, “E”, and the degree symbol are omitted when giving decimal latitude and longitude.

line 681, change “Transcriptome completeness was done” to “Transcriptome completeness was quantified”
line 703 italicize “Schmidtea mediterranea”
line 717, change “identified Guo” to “identified by Guo”
line 1157, ref 77, give authors.

Reviewer #3

(Remarks to the Author)
This manuscript by Brand and colleagues presents a haplotype-phased genome assembly of an asexual strain of the planarian Schmidtea mediterranea, alongside population genomic data. The authors report that chromosomal rearrangements suppress recombination across the genome without disrupting reproductive genes. Given that S. mediterranea is a well-established model for studying stem cell biology and regeneration, this work provides important insight into the genomic basis of its asexual reproduction—a process often regarded as an evolutionary dead end. Earlier cytogenetic work by Baguñà et al. (1999) suggested that a chromosomal translocation might underlie the asexual phenotype, but that conclusion was based on mitotic metaphase observations that could not resolve genome-scale structures. The lack of a haplotype-phased genome for the asexual strain has therefore long limited understanding of its genomic architecture. In this study, Brand et al. successfully reconstruct the chromosomal rearrangement history and show that the translocation previously implicated in asexuality is not directly responsible for the loss of sexual reproduction.

Overall, this is a timely and broadly relevant study that revisits the long-standing question of how chromosomal evolution shapes asexuality in S. mediterranea. Building on their earlier work on the sexual strain, the authors identify three successive inversions and a reciprocal translocation that together appear to have driven genome-wide suppression of recombination while leaving reproductive genes largely intact. These findings provide a coherent and updated evolutionary framework for understanding the genomic basis of asexual reproduction in this model organism. However, the manuscript would benefit from a clearer consideration of alternative structural models and more detailed discussion of the proposed mechanisms before it can be considered for publication.

Major comments:

1. The manuscript provides a well-integrated historical perspective on the sexual and asexual strains and presents new genomic evidence that refines their evolutionary timeline. Using high-quality genome assemblies, the authors estimate the divergence between the two lineages to be approximately 0.64 million years ago—substantially more recent than the previously inferred 8 million years. It would be valuable if the authors could include additional discussion or analysis addressing the biological implications of this revised divergence time, particularly in relation to possible geographic isolation or climatic factors that might have influenced the evolution of asexuality in S. mediterranea.

2. The generation of a haplotype-phased assembly represents a substantial advance, allowing detailed characterisation of chromosomal rearrangements beyond karyotype-level observations. The finding that the asexual strain CIW4 exhibits extensive haplotype divergence is striking, as it suggests that homologous recombination is effectively suppressed. The synteny analysis provides valuable insight into the structural complexity, although the rearrangements may involve more intricate events than a simple translocation between chromosomes 1 and 3. These findings highlight the need for a clearer explanation of the structural basis of these rearrangements and their evolutionary implications. The authors are encouraged to reorganise Fig. 3 to make this point more readily interpretable, particularly Fig. 3a, which is somewhat difficult to follow in its current form. These considerations lead to the following specific questions.

3. While the authors interpret these rearrangements as interchromosomal translocations, could a stepwise process of chromosome fusion, inversion, and subsequent fission also explain the observed pattern? This alternative scenario might better account for the conservation of homologous sequences at chromosome ends, which would be less expected under classical translocation. In Fig. 2b, for instance, the comparison between Chr2 and Chr2′ appears counter-intuitive: rather than an inversion occurring at the chromosome ends, the pattern of conserved chromosome ends with an inverted central region would be more consistent with a single large inversion spanning the internal part of the chromosome. The current depiction suggests that the light-green internal region remains uninverted while only the terminal region is inverted, which seems mechanistically less plausible. A similar situation is seen in the comparison between Chr4 and Chr4′, where inversion of the darker pink region alone would parsimoniously explain the data. Mechanistically, a single inversion mediated by double-strand break repair would be more consistent with the observed synteny patterns than multiple independent end inversions.

4. Following this reasoning, the authors might wish to explore whether a more parsimonious explanation of the structural differences could be proposed. For instance, the observed configuration of Chr1 and Chr1′ might be consistent with a fusion event between chromosomes 1 and 3, followed by multiple inversions and a subsequent fission. Such an alternative reconstruction could involve the following steps (regions defined according to the colour codes in Fig. 2b). This scenario may provide a more parsimonious explanation for the structural variation observed between haplotypes.

Step 1: Fusion of Chr1 and Chr3, resulting in the combination of Region4 (orange), Region3 (dark crimson), Region2 (light peach), and Region1 (brick red) from Chr1 with Region6 (blue) and Region5 (dark cyan) from Chr3, according to the current presentation. This step does not require an inversion per se, as the orientation can be accounted for by the fusion itself.
Step 2: Inversion of the fused Chr1–Chr3 structure, involving Region3 (dark crimson), Region2 (light peach), Region1 (brick red), and Region6 (blue) situated between Region4 (orange) and Region5 (dark cyan). This would produce the arrangement: Region4 (orange), Region6 (blue), Region1 (brick red), Region2 (light peach), Region3 (dark crimson), Region5 (dark cyan).
Step 3: Inversion of Region6 (blue), Region1 (brick red), and Region2 (light peach).
Step 4: Inversion of Region1 (brick red) and Region6 (blue).
Step 5: Fission between Region1 (brick red) and Region6 (blue).

5. The authors are encouraged to discuss whether such alternative scenarios could also account for the observed haplotype divergence. Presenting alternative models side by side—especially if supported by synteny maps—could strengthen the interpretation of the chromosomal rearrangements.

6. To further clarify the evolutionary scenario, it would be useful to include a direct synteny comparison between the sexual strain (S2F18, presumably representing the ancestral condition) and the asexual strain (CIW4).

Minor comments:

7. In Fig. 2b, please define the notations “der()”, “t()”, and “inv()” in the legend for clarity.

8. There appears to be a bibliography formatting error preventing the proper display of references. This issue should be corrected to allow reviewers to verify key citations and assess the study in its broader context during revision.

9. In the Data Availability section, placeholders (XXX) remain. These should be replaced with actual accession numbers or links to ensure transparency and reproducibility.

Version 2:

Reviewer comments:

Reviewer #1

(Remarks to the Author)
The authors have addressed succesfully all the concerns raised. Congrats on a very nice piece of work.

Reviewer #2

(Remarks to the Author)
The authors have done a good job of addressing reviewer comments. In my view, the manuscript is ready to publish. Congratulations to the authors on their excellent work.

Reviewer #3

(Remarks to the Author)
The authors have addressed my comments well. I have no further comments and congratulate them on providing this important genomic resource to the community.

Open Access This Peer Review File is licensed under a Creative Commons Attribution 4.0 International License, which permits use, sharing, adaptation, distribution and reproduction in any medium or format, as long as you give appropriate credit to the original author(s) and the source, provide a link to the Creative Commons license, and indicate if changes were made.
In cases where reviewers are anonymous, credit should be given to 'Anonymous Referee' and the source.
The images or other third party material in this Peer Review File are included in the article's Creative Commons license, unless indicated otherwise in a credit line to the material. If material is not included in the article's Creative Commons license and your intended use is not permitted by statutory regulation or exceeds the permitted use, you will need to obtain permission directly from the copyright holder.
To view a copy of this license, visit https://creativecommons.org/licenses/by/4.0/

RESPONSE TO REVIEWERS' COMMENTS

Reviewer #1 (Remarks to the Author):

I commend the authors for an excellent and carefully executed piece of work on a very interesting and timely topic. The manuscript presents a technically sophisticated and conceptually ambitious study that investigates the genomic underpinnings of asexual reproduction in Schmidtea mediterranea. The integration of long-read sequencing, Hi-C–based chromosomal architecture, cytogenetics, and population genomic analyses represents a major advance for the field. The resulting haplotype-phased genome assembly of the asexual strain CIW4 is of very high quality and will undoubtedly become an important resource for the planarian community. The paper also has broad relevance for evolutionary genomics, as it addresses fundamental questions about how recombination suppression and chromosomal rearrangements relate to the evolution of asexuality.

Response:

Thank you for recognizing the interesting and timely nature of our work.

While the study is technically impressive and the data are of high quality, several key interpretations, particularly those linking chromosomal rearrangements to the origin of asexuality, remain correlative rather than causal. This distinction between correlation and causation underpins most of my concerns. The authors convincingly show that extensive structural rearrangements are present in the asexual genome and that they predate or accompany the loss of sex, but they do not provide direct evidence that these rearrangements caused the transition to fissiparous reproduction. In its current form, the paper somewhat overstates this causal link, and the discussion would benefit from a more balanced consideration of alternative explanations.

- Correlation versus causation:

The central model proposed—whereby stepwise recombination suppression progressively eroded the benefits of sex and facilitated the emergence of asexuality—is elegant but not fully demonstrated. The data clearly support that inversions and translocations occurred in a sexual ancestor and that recombination was reduced in affected regions. However, whether this suppression directly triggered the loss of sexual reproduction, or whether both phenomena resulted from other demographic or developmental factors, remains unresolved. The authors should temper the causal language throughout the manuscript and frame their model as one plausible scenario among several. Phrasing such as "may have facilitated" or "is consistent with" would more accurately reflect the evidence.

Response:

Thank you and we agree. We have carefully revised the manuscript throughout to temper causal statements and to explicitly frame our model as one plausible scenario consistent with the data rather than a definitive explanation.

Specifically, we have made the following changes:

- In the abstract we now write “Altogether, our results are consistent with a model” instead of “Altogether, our results support a model”
- The final sentence of the introduction now reads: “Based on our findings, we propose a stepwise model for the evolution of asexuality in S. mediterranea, [...]” instead of “Our support the stepwise evolution of asexuality in S. mediterranea, [...]”
- In the discussion, the text now reads “Specifically, the chromosomal rearrangements may have given rise to balanced-lethality systems” instead of “Specifically, the chromosomal rearrangements gave rise to balanced-lethality systems”
- In a later section of the discussion, the text now reads: “Taken together, our model represents one interpretation consistent with the data, in which adaptive chromosomal rearrangements progressively undermined the efficiency and genomic benefits of sex [...]” instead of “Taken together, our model posits that [...]”

We feel that the text now accurately distinguishes between facts and interpretation, and that the discussion provides a balanced view of the alternatives without overstating the causal conclusions that can be drawn from this observational study (see next point).

Moreover, it would strengthen the manuscript to explicitly test or at least discuss alternative hypotheses that could generate similar genomic patterns. For example, the heterozygosity and linkage disequilibrium signatures attributed to recombination suppression could also arise from demographic events, such as bottlenecks, isolation, or hybridization between divergent sexual populations. Hybrid origin in particular could produce highly heterozygous, structurally rearranged genomes and impaired meiosis, leading to secondary asexuality—a scenario that has been documented in many other asexual systems. The authors might also consider that the structural rearrangements could have evolved under local adaptation or drift in small island populations, with the loss of sex following later due to regulatory or epigenetic changes in reproductive genes. These possibilities do not contradict their data but offer alternative, potentially more parsimonious explanations that should be acknowledged.

Response:

We thank the reviewer for highlighting scenarios that could be complementary explanations for the presented data. We are unfortunately not able to devise a test to

differentiate such scenarios. We have expanded the Discussion to explicitly address local adaptation, drift in island populations, and hybrid origin as alternative explanations

- We have added a paragraph discussing the role of hybridization and polyploidization: "In an alternative scenario the structural rearrangements observed in LabAsex and MenAsex as well as the disruption of germline development could have arisen via hybridization between highly diverged sexual populations. Evidence for this scenario comes from the presence of a triploid population of an asexual strain in Menorca, which, based on cytological evidence, appears to share the Chr1/3 translocation. Triploidy in *S. mediterranea* could arise from meiotic disruption in hybrids between highly diverged diploid populations, leading to the production of unreduced (diploid) gametes that, upon crossing with a diploid sexual partner, would produce triploid offspring[72]. Under this model, the structural differences could have emerged rapidly during the hybridization event. However, as mentioned above, it is unclear how the rearrangement-dependent pattern of heterozygosity would have arisen. Phylogenetic analysis that includes triploid strains from both Sardinia and Menorca would be required to shed more light on this scenario."
- In the following section we added a sentence highlighting the potential role of local adaptation. "). In addition to shielding recessive deleterious alleles from inbreeding effects[78], reduced recombination may have facilitated the maintenance of locally adapted haplotypes by limiting their breakdown through recombination with maladapted migrant alleles[78,79]. This effect may have been particularly relevant in geographically isolated island populations, where restricted gene flow and heterogeneous environmental conditions could favor the accumulation and persistence of locally beneficial allele combinations within rearranged chromosomal regions."

- Gene loss in sexual genes:

An additional point concerns the interpretation of the gene loss analysis near chromosomal breakpoints. While the authors convincingly show that no coding genes are physically disrupted by inversions or translocations, the conclusion that these rearrangements have no functional impact may be premature. Even if genes remain intact at the sequence level, their regulatory function could have been altered through changes in chromatin topology, enhancer–promoter interactions, or higher-order gene regulatory networks. Given that the authors already generated high-quality Hi-C data, they have everything needed to explore whether these structural rearrangements

modify the connectivity of regulatory domains around key reproductive genes. Integrating such chromatin interaction data with transcriptomic profiles could reveal whether changes in genome architecture correspond to altered expression dynamics rather than to physical gene loss. This would allow the authors to move beyond the absence of coding gene loss and directly assess whether genome restructuring has affected the functional regulation of sexual development pathways.

Response:

We agree and attempted to detect such effects using Hi-C data but were unable to identify a high-confidence signal. This may reflect the use of bulk Hi-C from whole animals, which could mask tissue-specific regulatory effects, or a limited role of higher-order chromatin structure in planarian gene regulation.

We have added supplementary Hi-C comparisons at 1 Mb genome-wide resolution and 25 kb resolution at breakpoint regions. We now write:

"These results suggest that the large-scale structural rearrangements themselves did not disrupt coding genes. In addition, a comparison of Hi-C signals within 10 Mb flanking the breakpoint did not reveal any overt changes in chromatin conformation, consistent with a lack of regulatory effects (Supplementary Note 3)."

- Functional annotation:

Although the authors describe the use of eggNOG-mapper, InterProScan, and DIAMOND for gene annotation, they do not provide quantitative information on annotation completeness, such as the percentage of genes with functional annotation, GO terms, or orthology assignments (or at least I have not found them, but they may be reported in the supplementary perhaps). Reporting these summary statistics would considerably enhance the transparency and reproducibility of the work, as well as enable meaningful cross-species comparisons. Based on my experience, these annotation pipelines typically leave a substantial fraction of genes unannotated, particularly in non-model and compositionally biased genomes such as planarians. It would therefore be valuable for the authors to assess the proportion of unannotated genes and consider complementary approaches to improve functional annotation, such as recently developed methods based on large language models (eg, FANTASIA) or deep learning (eg, DeepGoPlus), for instance.

Response:

We have added quantitative summary plots of functional annotation completeness. We now write:

“Functional annotation based on homology searches and domain predictions enabled annotation of 70% of all coding genes (Supporting Information: Gene annotation). “

A 70% efficiency is satisfying for the analyses presented here. We have also previously explored FANTASIA and an approach based on a combination of AlphaFold2 and FoldSeek. We saw that AlphaFold2-based predictions can lead to improvement in annotation but it was not computationally feasible to perform such a search genome-wide. Our functional annotation pipeline has been integrated into the planmine database and will be made public with the next upcoming release.

- Divergence times inference:

A further point worth considering concerns the reliability of the molecular dating analyses in the context of S. mediterranea’s extreme structural genome dynamics. As shown by their previous work in Ivanković et al. (2024), planarian genomes exhibit exceptionally high rates of chromosomal rearrangement and a general lack of conserved synteny, even among closely related lineages. If synteny is largely uncoupled from evolutionary constraint in these animals, then clock-like assumptions based on sequence divergence alone may not accurately capture the true timing of lineage splits. In this sense, the inferred divergence between the sexual and asexual strains, already surprisingly recent, should be interpreted cautiously. The authors could strengthen this section by explicitly discussing how structural volatility and relaxed synteny conservation may affect the robustness of their dating estimates, and by exploring whether alternative calibration approaches (e.g., using gene families with conserved chromosomal context or benchmarking against more stable taxa) yield consistent results.

Response:

We utilized three independent strategies for divergence estimation. Their convergence on highly similar estimates suggests that these results are robust, even within the context of a dynamic genome.

1. Orthology and Synteny Conservation: The concern regarding structural dynamics potentially compromising degenerate site analysis primarily hinges on orthology inference. However, while Ivanković et al. (2024) highlights synteny loss between planarians and distantly related flatworm groups (e.g., tapeworms, flukes), synteny remains high within the genus Schmidtea. Despite frequent chromosome fissions and fusions, syntenic blocks between species are relatively large (Median lengths: 1.5–2.2 Mb), and gene-order synteny is highly conserved. This conservation allowed us to use GENESPACE to identify high-confidence, single-copy orthologs. We further validated these assignments using TOGA (a whole-genome alignment tool). Crucially, any misassignment of orthologs or inclusion of paralogs would typically lead to an *overestimation* of divergence, rather than the surprisingly recent split observed.

2. Population Genomics (PSMC): To ensure that structural volatility did not interfere with the PSMC analysis, we applied stringent mapping and filtering criteria. These measures prevent segmental duplications or repetitive elements common in dynamic genomes from creating false heterozygosity signals, which would otherwise skew the timing estimates.

3. LTR Transposon Dating: Our third approach, using LTR transposon insertion dating, is notably robust against large-scale structural rearrangements. This method is effective even in polyploid organisms or those with recent whole-genome duplications, providing a structural-independent benchmark that aligns with our sequence-based estimates.

We have added a discussion regarding synteny conservation in this context and now refer to Supplementary Note 6, which visually demonstrate the maintained synteny. While we acknowledge these are broad estimates, the consistency across three distinct methodologies—including those insensitive to structural divergence—provides strong evidence for the recent nature of these lineage splits.

- Presentation and figures:

The manuscript is generally well written and well organized, though somewhat dense in parts and difficult to follow, particularly with the acronyms of the populations, etc. The figures are clear but could be simplified in places. Some secondary analyses might be moved to Supplementary Figures to enhance readability. Methods are described in commendable detail and appear sufficient for reproducibility, but sequencing depth per sample and the exact filtering thresholds for evolutionary analyses should be reported in the main text or figure legends, I feel that the authors refer to previous work too often (eg, genome assembly methods, etc.). On the other hand, there are parts, such as the genome assembly and annotation of the asexual strain in Results, that could go almost directly to Supplementary Material. The data availability statement should include accession numbers for all newly generated sequencing data to ensure transparency, which I'm sure will be fixed in later revisions.

Response:

We agree that the acronyms of the populations can be confusing. To reduce confusion, we have reduced the mention of the strain abbreviations and instead consistently refer to LabAsex and LabSex for the laboratory populations and refer to MenAsex and SarSex for the wild populations.

We have added information on sequencing depth and applied filters in the Results and Figure legends:

- In the Result section ‘with 44x coverage PacBio HiFi and 137x coverage Hi-C sequencing coverage’
- In the figure legend of Figure 2: ‘**a** Hi-C chromatin interaction heatmap on haplotype 1 of the LabAsex assembly based on 137x Hi-C coverage from the LabAsex strain.’
- Figure Legend 4 **g-j** Tests for costs of asexuality using whole-genome sequencing at 30x coverage
- Figure 5: **f** Hi-C matrix on haplotype 1 of the LabAsex assembly for 137x coverage Hi-C data from LabAsex (bottom triangle) and 113x coverage Hi-C data from MenAsex (top triangle),
- In the legend for Figure 4: ‘For $\pi N/\pi S$ analyses only conserved genes ($\pi N/\pi S < 1$) were included in the analysis.‘

We retain genome assembly results in the main text because they represent a substantial advance for the field.

We have also kept the references to previous work as this is primarily our recent paper on genome evolution in Planarians, which we cite for our wetlab methods related to DNA extraction.

Reviewer #2 (Remarks to the Author):

The authors present a phased and annotated genome of the asexual laboratory strain of Schmidtea mediterranea flatworms, along with an in-depth population-genomic study of wild asexual and sexual populations. The volume of genomic data and the thoroughness of the analyses is impressive. The conclusions about the history of the genomes and populations is largely persuasive. I have one substantive comment and a number of minor copy-editing suggestions:

Substantive comment

The suggestion that fissiparity can partially overcome the cost of sex by avoiding the one-cell bottleneck is an interesting one. Kondrashov made an opposite argument (Kondrashov, A. S. (1994). Mutation load under vegetative reproduction and cytoplasmic inheritance. Genetics, 137(1), 311–318.), which the authors do not cite. The authors should at least cite that article and ideally explain why it is invalid or does not apply.

Response:

Thank you for the suggestion. We are aware of the work by Kondrashov and do not consider it an issue for our argument. While Kondrashov indeed showed that under certain assumptions, vegetative reproduction will always accrue at least the same mutational load as apomixis and in many scenarios increase the load substantially. However, Kondrashov did not account for somatic selection. We therefore cite Otto and Orive (1995),

who showed that somatic selection can drastically reduce the mutational load even below the level for organisms with a single-cell bottleneck, such as apomixis or mixis.

We have added a sentence to make this important distinction explicit without the need to consult the reference. The relevant section now reads:
"On the other hand, the lack of mutational meltdown in our data and the abundance of asexually reproducing strains and species might indicate the existence of particularly effective means of purging deleterious mutations in flatworms. One key mechanism could be somatic selection. Early theoretical work on mutational load under vegetative reproduction predicted that the load is always at least as high as under sexual reproduction and often substantially higher[1]. Subsequent models, however, have shown that incorporating somatic selection can lead to efficient purging of deleterious mutations[1,2]."

Otto, S. P., and M. E. Orive. 1995. Evolutionary consequences of mutation and selection within an individual. Genetics 141:1173–1187.

Copy-editing suggestions

lines 67- 68, change "sexual reproduction, parthenogenesis" to sexual reproduction, to parthenogenesis"

The sentence now reads:
"[...]fascinating spectrum of reproductive strategies, ranging from hermaphroditic sexual reproduction, to parthenogenesis, and asexual fissiparous reproduction."

line 124, change "mercury" to "merqury"

Done.

line 508, change, "which exhibit" to "which exhibits"

Done.

line 517, change "Rotifers" to "rotifers"

Done.

lines 595-596, is the reported degree of precision in coordinates of collection sites (to

nearest 1m in Menorca and to nearest 10cm in Sardinia) justified? Typically, "N", "E", and the degree symbol are omitted when giving decimal latitude and longitude.

Thank you for pointing this out. We now give the coordinates with 10m accuracy and have removed the "N" and "E" and degree symbols.

line 681, change "Transcriptome completeness was done" to "Transcriptome completeness was quantified"

Done.

line 703 italicize "Schmidtea mediterranea"

Done.

line 717, change "identified Guo" to "identified by Guo"

Done.

line 1157, ref 77, give authors.

Done.

Reviewer #3 (Remarks to the Author):

This manuscript by Brand and colleagues presents a haplotype-phased genome assembly of an asexual strain of the planarian Schmidtea mediterranea, alongside population genomic data. The authors report that chromosomal rearrangements suppress recombination across the genome without disrupting reproductive genes. Given that S. mediterranea is a well-established model for studying stem cell biology and regeneration, this work provides important insight into the genomic basis of its asexual reproduction—a process often regarded as an evolutionary dead end. Earlier cytogenetic work by Baguñà et al. (1999) suggested that a chromosomal translocation might underlie the asexual phenotype, but that conclusion was based on mitotic metaphase observations that could not resolve genome-scale structures. The lack of a haplotype-phased genome for the asexual strain has therefore long limited understanding of its genomic architecture. In this study, Brand et al. successfully reconstruct the chromosomal rearrangement history and show that the translocation previously implicated in asexuality is not directly responsible for the loss of sexual reproduction.

Overall, this is a timely and broadly relevant study that revisits the long-standing question

of how chromosomal evolution shapes asexuality in S. mediterranea. Building on their earlier work on the sexual strain, the authors identify three successive inversions and a reciprocal translocation that together appear to have driven genome-wide suppression of recombination while leaving reproductive genes largely intact. These findings provide a coherent and updated evolutionary framework for understanding the genomic basis of asexual reproduction in this model organism.

Thank you for recognizing the timeliness and broad relevance of our work.

However, the manuscript would benefit from a clearer consideration of alternative structural models and more detailed discussion of the proposed mechanisms before it can be considered for publication.

We thank you and reviewer 1 for pointing out the narrow focus of the original discussion. Combining your suggestions, we have now broadened the scope of the discussion (see below).

Major comments:

1. The manuscript provides a well-integrated historical perspective on the sexual and asexual strains and presents new genomic evidence that refines their evolutionary timeline. Using high-quality genome assemblies, the authors estimate the divergence between the two lineages to be approximately 0.64 million years ago—substantially more recent than the previously inferred 8 million years. It would be valuable if the authors could include additional discussion or analysis addressing the biological implications of this revised divergence time, particularly in relation to possible geographic isolation or climatic factors that might have influenced the evolution of asexuality in S. mediterranea.

Response:

Thank you for this interesting suggestion. However, we are not paleo-climate experts and feel that amateur-speculations on basis of a superficial reading of the literature would not be helpful. To stimulate potential follow-up work on this topic by experts in the field, we included the following sentence in the discussion:

“While an examination of regional paleo-climate records may provide further context for this revised timeline, our data are currently consistent with two alternative population history scenarios.”

2. The generation of a haplotype-phased assembly represents a substantial advance, allowing detailed characterisation of chromosomal rearrangements beyond karyotype-level observations. The finding that the asexual strain CIW4 exhibits extensive haplotype divergence is striking, as it suggests that homologous recombination is effectively suppressed. The synteny analysis provides valuable insight into the structural complexity,

although the rearrangements may involve more intricate events than a simple translocation between chromosomes 1 and 3. These findings highlight the need for a clearer explanation of the structural basis of these rearrangements and their evolutionary implications. The authors are encouraged to reorganise Fig. 3 to make this point more readily interpretable, particularly Fig. 3a, which is somewhat difficult to follow in its current form. These considerations lead to the following specific questions.

Thank you- we address these points below.

3. While the authors interpret these rearrangements as interchromosomal translocations, could a stepwise process of chromosome fusion, inversion, and subsequent fission also explain the observed pattern? This alternative scenario might better account for the conservation of homologous sequences at chromosome ends, which would be less expected under classical translocation.

Response:

Thank you for pointing out that Figure 2 is unnecessarily complicated. In relation to the arrangement of the derived Chr1' and Chr3' we posit a regular reciprocal translocation. The preservation of homology at the chromosome ends is likely due to the preceding inversion of the central region of the chromosome. We have modified the Figure 2e legend and the text to make this clearer:

- The legend Fig. 2e now reads: "Schematic representation of the most parsimonious sequence of chromosome rearrangements. The reciprocal Chr1/3 translocation most likely involved an already inverted Chr1. Although the rearrangements involving Chr2 and Chr4 could, in principle, have occurred earlier, the fact that the Chr1 inversion is also present in the LabSex strain—which lacks the other rearrangements—suggests that the Chr1 inversion occurred first, with the remaining rearrangements accumulating subsequently in the asexual lineage (Supporting Information: Comparison of Hi-C signals on Chr1)."
- The text has been modified to now read: "Our data suggest the succession of chromosomal translocations and inversions depicted in Fig. 2e as the most parsimonious evolutionary explanation for the current asexual genome structure. Importantly, if the Chr1 inversion preceded the Chr1/3 translocation this explains the preservation of homology at chromosome ends with only two events, while possible alternative scenarios would require at least three interchromosomal translocations or even more complex sequences of fission, fusions, and inversions."

In Fig. 2b, for instance, the comparison between Chr2 and Chr2' appears counter-intuitive: rather than an inversion occurring at the chromosome ends, the pattern of conserved chromosome ends with an inverted central region would be more consistent with a single large inversion spanning the internal part of the chromosome. The current depiction

suggests that the light-green internal region remains uninverted while only the terminal region is inverted, which seems mechanistically less plausible. A similar situation is seen in the comparison between Chr4 and Chr4', where inversion of the darker pink region alone would parsimoniously explain the data. Mechanistically, a single inversion mediated by double-strand break repair would be more consistent with the observed synteny patterns than multiple independent end inversions.

Response:

The orientation of LabSex haplotype 2 as shown in Fig. 2b represent the orientation of the genome assembly. However, designation of + and – strand are conventions and the assembled chromosomes can be inverted if it is desired. Consequently, while Chr2 can be interpreted as showing two translocations, these are topologically equivalent to one central inversion. Based on the reviewer feedback we have now inverted Chr2 haplotype 2 to aid in the interpretation and make it explicit (as also stated in the text) that we assume one inversion not two independent translocations. We have chosen not to invert Chr4 since we found that the inverted configuration was not easier to interpret.

4. Following this reasoning, the authors might wish to explore whether a more parsimonious explanation of the structural differences could be proposed. For instance, the observed configuration of Chr1 and Chr1' might be consistent with a fusion event between chromosomes 1 and 3, followed by multiple inversions and a subsequent fission. Such an alternative reconstruction could involve the following steps (regions defined according to the colour codes in Fig. 2b). This scenario may provide a more parsimonious explanation for the structural variation observed between haplotypes.

Step 1: Fusion of Chr1 and Chr3, resulting in the combination of Region4 (orange), Region3 (dark crimson), Region2 (light peach), and Region1 (brick red) from Chr1 with Region6 (blue) and Region5 (dark cyan) from Chr3, according to the current presentation. This step does not require an inversion per se, as the orientation can be accounted for by the fusion itself.
Step 2: Inversion of the fused Chr1–Chr3 structure, involving Region3 (dark crimson), Region2 (light peach), Region1 (brick red), and Region6 (blue) situated between Region4 (orange) and Region5 (dark cyan). This would produce the arrangement: Region4 (orange), Region6 (blue), Region1 (brick red), Region2 (light peach), Region3 (dark crimson), Region5 (dark cyan).
Step 3: Inversion of Region6 (blue), Region1 (brick red), and Region2 (light peach).
Step 4: Inversion of Region1 (brick red) and Region6 (blue).
Step 5: Fission between Region1 (brick red) and Region6 (blue).

5. The authors are encouraged to discuss whether such alternative scenarios could also account for the observed haplotype divergence. Presenting alternative models side by side—especially if supported by synteny maps—could strengthen the interpretation of the chromosomal rearrangements.

Response:

The scenario outlined by us for the origin for Chr1 and Chr1’ requires two events:

1. Inversion of the central part of Chr1. This is the inversion already observed in the sexual strain.
2. A reciprocal translocation between Chr3 and Chr1

The alternative scenario that you outline is less parsimonious, because it would require that inversions occurred in both the sexual and asexual strain and independently at exactly the same break point in Chr1. Nevertheless, we have expanded our discussion of the results to make it clearer that more complex scenarios are possible.

- The text now reads: “Our data suggest the succession of chromosomal translocations and inversions depicted in Fig. 2e as the most parsimonious evolutionary explanation for the current asexual genome structure. Importantly, if the Chr1 inversion preceeded the Chr1/3 translocation this explains the preservation of homology at chromosome ends with only two events, while possible alternative scenarios would require at least three interchromosomal translocations or even more complex sequences of fission, fusions, and inversions.”

6. To further clarify the evolutionary scenario, it would be useful to include a direct synteny comparison between the sexual strain (S2F18, presumably representing the ancestral condition) and the asexual strain (CIW4).

Response:

We show the synteny between both strains in the supporting information and now refer to it from the main text:

“Despite these substantial large-scale rearrangements, synteny between LabAsex and LabSex is highly conserved and can be used to reconstruct the evolutionary history of structural changes (Supporting Information: Synteny between LabSex and LabAsex).”

Minor comments:

7. In Fig. 2b, please define the notations “der()”, “t()”, and “inv()” in the legend for clarity.

Done- we have added an explanation of the symbols to the figure legend.

8. There appears to be a bibliography formatting error preventing the proper display of references. This issue should be corrected to allow reviewers to verify key citations and assess the study in its broader context during revision.

We apologize for this error. The bibliography is now formatted correctly.

9. In the Data Availability section, placeholders (XXX) remain. These should be replaced with actual accession numbers or links to ensure transparency and reproducibility.

We have now added identifiers for the requested data. They will be made public with the publication of the paper.
